# Systematic review and meta-analysis of goal-directed haemodynamic therapy algorithms during surgery for the prevention of surgical site infection

**DOI:** 10.1016/j.eclinm.2024.102944

**Published:** 2024-11-22

**Authors:** Hasti Jalalzadeh, Rick H. Hulskes, Robert P. Weenink, Niels Wolfhagen, Ingeborg van Dusseldorp, Roald R. Schaad, Denise P. Veelo, Markus W. Hollmann, Marja A. Boermeester, Stijn W. de Jonge

**Affiliations:** a Department of Surgery, Amsterdam UMC, Location the University of Amsterdam, Amsterdam, the Netherlands; b Amsterdam Gastroenterology Endocrinology & Metabolism, Amsterdam, the Netherlands; c Dutch National Guideline Group for Prevention of Postoperative Surgical Site Infections; d Department of Anaesthesiology, Amsterdam UMC, Location the University of Amsterdam, Amsterdam, the Netherlands; e Knowledge Institute for the Federation of Medical Specialists, Utrecht, the Netherlands

**Keywords:** Systematic review, Meta-analysis, Goal-directed haemodynamic therapy, Surgical site infection, Wound infection, Surgery, Anaesthesiology

## Abstract

**Background:**

Surgical site infection (SSI) is the most common postoperative complication. Goal-directed haemodynamic therapy (GDHT) may help to prevent SSI, but recommendations for its use initially have been set at conditional because of low-certainty evidence at the time. An updated systematic review with SSI as the primary endpoint has not been performed since 2011, and important new evidence has emerged. We assessed the influence of GDHT on SSI and other postoperative outcomes.

**Methods:**

We searched Ovid/MEDLINE, Excerpta Medica Database (Embase.com), and Cochrane library from inception up to September 2024 for randomised controlled trials comparing the effect of any GDHT algorithm to conventional fluid therapy on SSI incidence in adult patients undergoing surgery and analysed eligible data using random effects. We conducted several subgroup analyses, including the risk of bias (RoB), and a trial sequential analysis (TSA). We evaluated the certainty of evidence using Grading of Recommendations, Assessment, Development, and Evaluations. This study is registered with PROSPERO, CRD42022277535.

**Findings:**

We found 75 studies that met the inclusion criteria with an incidence of 1,478 SSI among 13,010 patients (11.4%). The incidence of SSI was reduced from 13.3% in the conventional fluid therapy to 9.4% after GDHT (absolute risk reduction 3.9%); pooled relative risk 0.71 (95% CI 0.62–0.81). Subgroup analysis for the low RoB studies revealed comparable results. Meta-regression indicated no strong evidence for individual subgroup effects. In the TSA, the cumulative z-line crossed the boundary for effect.

**Interpretation:**

High-certainty evidence indicates that GDHT reduces the risk of SSI when compared to conventional fluid therapy in adults undergoing surgery. New studies are unlikely to change this outcome. These findings justify a stronger recommendation for the use of GDHT.

**Funding:**

Dutch Association for Quality Funds Medical Specialists.

**Research in context**

**Evidence before this study**

Surgical site infections (SSI) and other postoperative complications pose a significant challenge, particularly with the growing threat of antimicrobial resistance. Goal-directed haemodynamic therapy (GDHT) is a highly investigated intervention to prevent several postoperative complications, including SSI. Recommendations by international healthcare authorities for perioperative GDHT are conditional and based on low-certainty evidence, resulting in limited implementation. Currently, GDHT is primarily reserved for patients undergoing high-risk surgery, while it may also benefit other patients. A recent meta-analysis provided new evidence, but focussed on mortality, rather than SSI. Previous meta-analyses designed to assess SSI have become outdated.

Furthermore, data on GDHT are heterogeneous with a great variety of populations, algorithms and targets.^1^ To help define the appropriate indication and application of perioperative GDHT, a new framework - “5 T's” of GDHT - has been proposed: adequate target population, timing of the intervention, type of intervention, target variable, and target value. This framework should lead to less heterogeneous results.^2^ So far, no meta-analysis has incorporated this framework of GDHT.

We searched Ovid/MEDLINE, Excerpta Medica Database (Embase.com), and the Cochrane Library for randomised controlled trials (RCTs) up to September 12, 2024, comparing any GDHT with conventional fluid management during surgery with the outcome of SSI. We identified 36 additional RCTs reporting on SSI compared to the most recent systematic review.

**Added value of this study**

This systematic review and meta-analysis of 75 RCTs, comprising 13,010 patients with 1,478 SSI, found high-certainty of evidence that GDHT is effective in reducing SSI (pooled relative risk of SSI was 0.71 (95% CI 0.62–0.81). This result was substantiated by a subgroup analysis for the low risk of bias studies. We found no strong evidence for individual subgroup effects. Trial sequential analysis indicated that additional RCTs are unlikely to change the effect.

**Implications of all the available evidence**

High-certainty of evidence showed that goal-directed haemodynamic therapy effectively reduces the incidence of postoperative SSI compared to conventional fluid management. The increased certainty of evidence justifies stronger recommendations for the use of GDHT.

## Introduction

Surgical site infection (SSI) is one of the most common healthcare-associated infections and a highly relevant cause of postoperative morbidity and mortality.^3^ SSI occurs in up to 20% of the more than 300 million patients globally undergoing inpatient surgery every year, putting considerable strain on available resources.^4^, ^5^, ^6^ The growth in patients undergoing surgery and the increasing number of patients with comorbidities and a high risk of SSI, combined with the emergence of antimicrobial resistance, further raises the stakes for the prevention of SSI.^6^, ^7^, ^8^, ^9^, ^10^, ^11^

Adequate tissue oxygenation is essential for wound healing, and conversely, perioperative tissue hypoxia is related to poor patient outcomes.^12^ Because hyper- and hypovolemia lead to impaired tissue oxygenation, meticulous perioperative fluid management is crucial to achieve adequate tissue oxygenation.^13^^,^^14^ Conventional fluid management is done at the anaesthetist's discretion based on clinical assessment and haemodynamic measurements, which may not adequately capture changes in intravascular volume.^15^^,^^16^ Both hypovolemia and hypervolemia remain prevalent in surgical patients.^13^^,^^14^

Goal-directed haemodynamic therapy (GDHT) algorithms have been developed to standardise and optimise fluid management. Specific haemodynamic targets guide intravenous fluid, vasopressor, and inotropic drug administration. International healthcare authorities recommend using such algorithms for the prevention of SSI, but these recommendations are conditional and based on low-certainty of evidence at the time of issue.^17^ This leads to limited implementation of GDHT, which is currently reserved for surgery with a high risk of mortality, while it may also benefit other patients.^18^^,^^19^ A recent meta-analysis on GDHT indicated important new evidence is available on mortality but SSI as important outcome was not included.^20^ Previous meta-analyses designed to assess SSI have become outdated, including those undertaken to develop existing guideline recommendations.

Data on GDHT are heterogeneous, with various algorithms and targets, complicating comparability and clinical applicability.^1^ To overcome this heterogeneity and facilitate adoption, a framework was proposed to help define the appropriate indication and application of perioperative GDHT summarised as “The ‘5 Ts' of perioperative goal-directed haemodynamic therapy” by Saugel and colleagues: adequate target population, timing of the intervention, type of intervention, target variable, and target value.^1^ Accordingly, analyses stratified according to the 5 T's should lead to less heterogeneous results.^2^ So far, no meta-analysis has incorporated this framework in a meta-analysis of GDHT.

To assess if guideline recommendations on the use of GDHT require revision after updating previous evidence and incorporating recent data and insights in the field of GDHT, we conducted a systematic review with meta-analysis and trial sequential analysis (TSA) of randomised controlled trials (RCTs) comparing the effect of any GDHT algorithm to conventional fluid therapy on SSI. We explored heterogeneity with meta-regressions and subgroup analyses in line with the 5 T's framework.

## Methods

### Search strategy and selection criteria

This systematic review and meta-analysis is reported according to the Preferred Reporting Items for Systematic Reviews and Meta-analysis (PRISMA) statement^21^ and was prospectively registered in the PROSPERO database (CRD42022277535).

We included RCTs comparing the effect of any GDHT algorithm to conventional fluid therapy on the incidence of SSI in adults undergoing surgery. GDHT was defined as any protocol for administering intravenous fluids, inotropes and vasoactive medication designed to reach a predefined haemodynamic target. Conventional fluid therapy was defined as any fluid therapy that did not meet this GDHT definition. All types of surgery were included. Studies investigating GDHT exclusively after surgery were excluded. No restriction on language or the year of publication was applied.

A medical information specialist (IvD) systematically searched Ovid/MEDLINE, Excerpta Medica Database (Embase.com), and the Cochrane Library for relevant literature published from inception of the database up to September 12, 2024. The following terms were used (including synonyms and closely related words) as index terms or free-text words: surgical site infection, postoperative wound infection, haemodynamic monitoring, fluid therapy, normovolaemia, goal-directed fluid therapy. The complete search strategy is presented in Appendix 1. Additional studies were identified by backward and forward citation tracing of previously published meta-analyses and included studies.

Two reviewers (HJ and RHH) independently reviewed the title and abstract for eligibility and the full-text review of the potentially eligible studies. Disagreements between the two reviewers were resolved through discussion, and if necessary, the senior authors (MAB and MWH) were consulted.

### Data analysis

Data were independently extracted by two authors (HJ and RHH) using a pre-specified form including author, year of publication, type and tools of GDHT, control groups, type of surgery, number of patients, American Society of Anesthesiologists' (ASA) classification,^22^ definitions of SSI and contamination according to the Centers for Disease Control and Prevention (CDC) wound classification.^23^ Authors were contacted for additional information if data were incomplete or unclear.

The primary outcome was the effect of GDHT on SSI as defined by the authors of the original publication and stratified according to the CDC classification (superficial, deep and organ space SSI).^24^ Secondary outcomes were mortality, sepsis, pneumonia, urinary tract infection (UTI), acute kidney injury (AKI), paralytic ileus, number of reoperations, and number of patients with complications.

Summary relative risks (RR), corresponding 95% confidence intervals (CI) and standard errors were calculated for the primary and secondary outcomes using a random-effects model (Mantel-Haenszel). We assessed statistical heterogeneity with the χ^2^ test and expressed the proportion of total heterogeneity due to between-study heterogeneity using *I*^2^ statistics. The extent of heterogeneity was evaluated using τ^2^. We used the empirical distribution for non-pharmacological comparisons on subjective outcomes to characterise the amount of heterogeneity as low, moderate or high using the first and third quantiles of their distribution.^25^

The Grading of Recommendations, Assessment, Development and Evaluation (GRADE) methodology was used to assess the certainty of evidence.^26^ The GRADE assessment consists of five domains: risk of bias (RoB), inconsistency, indirectness, imprecision, and publication bias. The (RoB) within individual studies was assessed independently by two authors (HJ and RHH) using the Cochrane Risk of Bias-2 tool.^27^ Inconsistency was graded based on statistical and clinical heterogeneity between studies. For assessing imprecision, meaningful benefit and harm were defined as an RR (reduction or increase) of 25%, and the confidence interval approach was used.^28^ If the effect is sufficiently large (RR reduction >30%), the optimal information size was also calculated, assuming a type I error (α) of 0.05, a type II error (β) of 0.2, and an RR reduction of 25%.^28^ A comparison-adjusted, contour-enhanced funnel plot was used to judge small-study effects.^29^

We conducted a subgroup analysis based on the RoB results. To explore heterogeneity we performed random effects meta-regression and subgroup analyses according to “The ‘5 Ts' of perioperative goal-directed haemodynamic therapy”.^1^ We defined the high-risk target population as studies with ≥50% of patients classified as ASA classification 3 or higher, adequate timing as GDHT algorithms initiated before induction of anaesthesia, adequate type of GDHT algorithm as algorithms incorporating fluids, vasopressors, or inotropes, adequate target variables as variables reflecting blood flow (stroke volume, stroke volume index, cardiac output, cardiac index, or oxygen delivery) and adequate target values as personalised target values.^1^ We performed meta-regression and subgroup analyses for each T and the total number of T's adhered to, and calculated the proportion of heterogeneity variance explained for each subgroup (τ^2^ primary analysis - τ^2^ meta-regression)/τ^2^ primary analysis). We also performed subgroup analysis based on wound contamination according to the CDC classification (clean, clean-contaminated, contaminated and dirty) because of their differences in baseline event rate and type of surgery (gastrointestinal, cardiothoracic and other) because of the differences in haemodynamic consequences.^23^

We conducted a TSA to assess the reliability and robustness of the primary outcome.^30^ Diversity-adjusted information size and estimated trial sequential monitoring boundaries were based on a type I error of 5%, a power of 80%, a RR reduction of 25% and an SSI risk in the conventional group of 13.9%, as found in the meta-analysis. We carried out a TSA sensitivity analysis limited to low RoB studies, with an SSI risk of 16.7% in the conventional group. When the cumulative Z-curve crosses into the trial sequential monitoring boundary, further data collection is unlikely to change the effect estimate.

Statistical analyses were done using R version 4.0.3 (R Foundation for Statistical Computing, Vienna, Austria, package “meta”). TSA was performed using TSA program version 0.9 beta (Copenhagen Trial Unit, Centre for Clinical Intervention Research, Rigshospitalet, Copenhagen, Denmark).

### Role of the funding source

The study was funded by the Dutch Association for Quality Funds Medical Specialists. The funder had no role in study design, data collection, data analysis, data interpretation, or writing of the report.

## Results

### Search

**Fig. 1:**
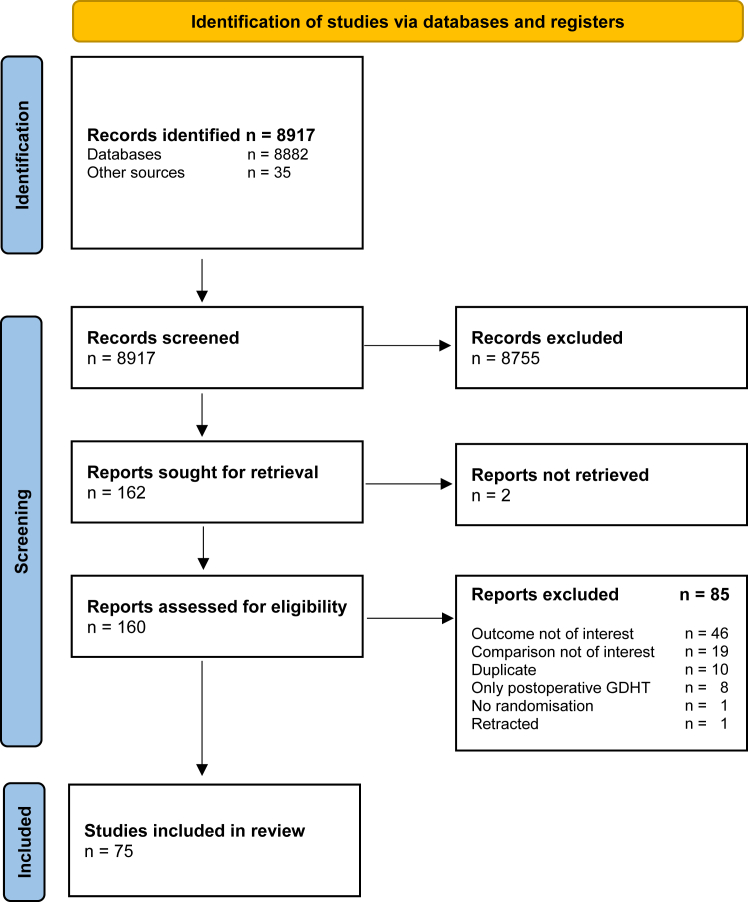
Study selection flow chart. GDHT: goal-directed haemodynamic therapy. Updated until September 12, 2024.

The database search resulted in 1,613 potential studies, and 35 additional studies were identified by backward and forward citation tracking. We assessed 160 full-text publications, of which 75 studies were included in the systematic review and meta-analyses. The selection process is shown in Fig. 1. The reason for exclusion after full-text review can be found in Appendix 2.

### Risk of bias

The detailed RoB assessment is presented in Appendix 3. Thirty-three RCTs were graded low RoB, 36 had some concerns regarding bias, and six were rated as high RoB.

### Study characteristics

The characteristics of the included studies can be found in Appendix 4. Multiple haemodynamic monitoring devices were used to perform GDHT, and the haemodynamic management goals and protocols varied greatly between studies. Most studies used one or more of the following variables: stroke volume, stroke volume variation, pulse pressure variation, cardiac index, and mean arterial pressure. Of the 75 included studies, 47 used a haemodynamic monitoring device connected to an arterial line to monitor the haemodynamic goals.^31^, ^32^, ^33^, ^34^, ^35^, ^36^, ^37^, ^38^, ^39^, ^40^, ^41^, ^42^, ^43^, ^44^, ^45^, ^46^, ^47^, ^48^, ^49^, ^50^, ^51^, ^52^, ^53^, ^54^, ^55^, ^56^, ^57^, ^58^, ^59^, ^60^^,^^61^, ^62^, ^63^, ^64^, ^65^, ^66^, ^67^, ^68^, ^69^, ^70^, ^71^, ^72^, ^73^, ^74^, ^75^, ^76^, ^77^ Fourteen studies used an oesophageal Doppler,^48^^,^^78^, ^79^, ^80^, ^81^, ^82^, ^83^, ^84^, ^85^, ^86^, ^87^, ^88^, ^89^, ^90^, ^91^ seven studies used a non-invasive haemodynamic monitoring sensor,^92^, ^93^, ^94^, ^95^, ^96^, ^97^, ^98^ one study used a device connected to a central venous line,^99^ one used blood sampling,^100^ one used the pulse variability index,^101^ and one study used the cardiopulmonary bypass device.^102^ Ten of the studies started with GDHT before the induction of anaesthesia.^32^^,^^34^^,^^36^^,^^45^^,^^50^^,^^63^^,^^69^^,^^76^^,^^92^^,^^96^ In 58 studies, GDHT was ceased at the end of the surgery,^31^^,^^33^, ^34^, ^35^^,^^38^, ^39^, ^40^^,^^42^^,^^43^^,^^45^^,^^46^^,^^49^, ^50^, ^51^, ^52^, ^53^, ^54^^,^^56^^,^^57^^,^^60^, ^61^, ^62^^,^^65^, ^66^, ^67^, ^68^^,^^70^, ^71^, ^72^, ^73^, ^74^, ^75^, ^76^^,^^78^, ^79^, ^80^, ^81^, ^82^, ^83^, ^84^, ^85^^,^^88^, ^89^, ^90^, ^91^, ^92^, ^93^, ^94^, ^95^, ^96^^,^^98^^,^^100^, ^101^, ^102^, ^103^, ^104^, ^105^ and in the remaining 17 studies GDHT was continued between one and 24 h postoperatively.^32^^,^^36^^,^^37^^,^^41^^,^^44^^,^^47^^,^^48^^,^^55^^,^^58^^,^^59^^,^^63^^,^^64^^,^^69^^,^^86^^,^^87^^,^^97^^,^^99^

### Primary outcome

**Fig. 2:**
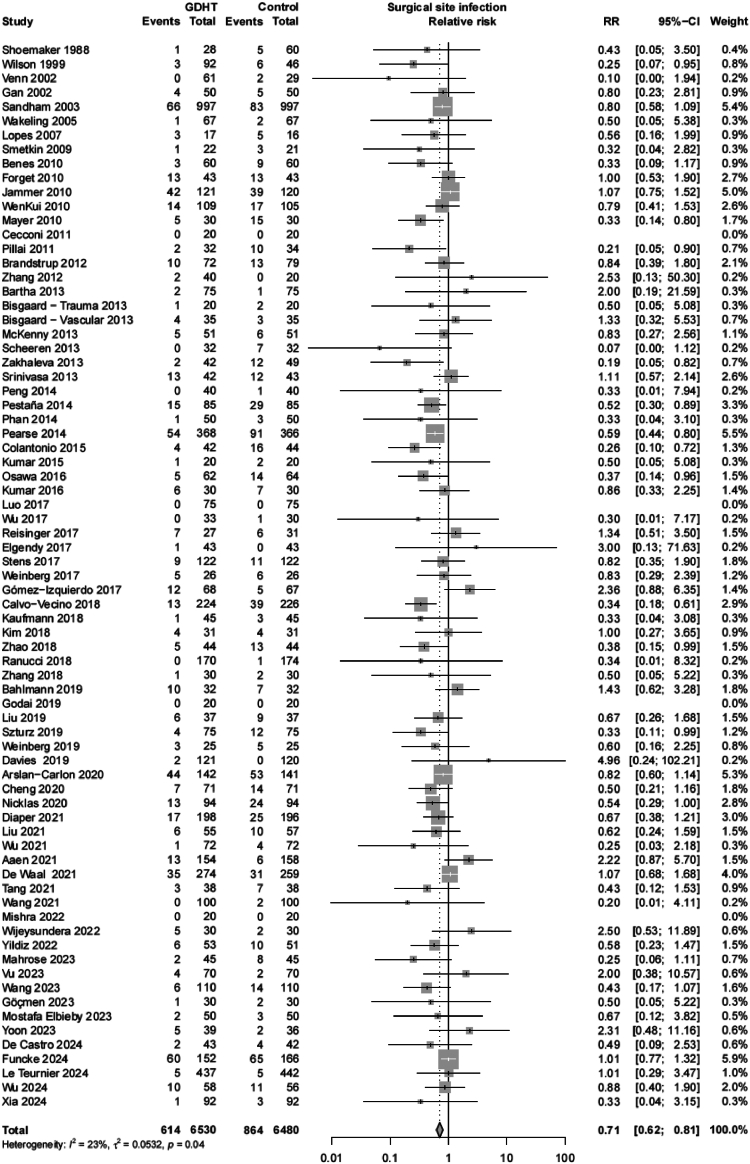
Primary analysis forest plot. Relative risk (RR) of surgical site infection (SSI) with goal-directed haemodynamic therapy (GDHT) versus no specific haemodynamic management. The figure shows the pooled RR. The squares and horizontal lines represent point estimates and corresponding 95% confidence intervals (CI) of the individual studies.

A total of 13,010 patients were included in the systematic review. Meta-analysis indicated that the incidence of SSI was reduced from 13.3% in the conventional fluid management group to 9.4% in the GDHT group (absolute risk reduction 3.9%), resulting in an pooled RR of 0.71 (95% CI 0.62–0.81). Heterogeneity between studies was low (τ^2^ = 0.053, *p* = 0.043, *I*^2^ = 23.5%). The result of the primary outcome is shown in Table 1 and Fig. 2.

**Table 1:** Meta-analysis of primary, secondary and subgroup analyses of the incidence of surgical site infection associated with goal-directed haemodynamic therapy.

	Number of studies	Surgical site infections/total patients in GDHT group	Surgical site infections/total patients in conventional group	Relative risk (95% confidence interval)	τ^2^ (meta-analysis)	τ^2^ (meta-regression)	p-value for subgroup differences	Percentage of heterogeneity variance explained
Surgical site infection	75	614/6530 (9.4%)	864/6480 (13.3%)	**0.71 (0.62–0.81)**	0.053			
T1 Target population: ASA classification 3 or higher						0.053	0.666	0.0
≥50% (High risk)	23	249/2734 (9.1%)	354/2723 (13.0%)	**0.62 (0.56–0.84)**	0.053			
<50% (Low risk)	52	365/3796 (9.6%)	510/3757 (13.6%)	**0.73 (0.61–0.86)**	0.070			
T2 Timing: start before induction						0.056	0.390	0.0
Yes	10	75/661 (11.3%)	94/646 (14.6%)	0.79 (0.61–1.03)	0.000			
No	65	539/5869 (9.2%)	770/5834 (13.2%)	**0.70 (0.60–0.80)**	0.072			
T3 Type of intervention: combination of fluids and inotropes						0.054	0.960	0.0
Yes	49	471/4844 (9.7%)	674/4883 (13.8%)	**0.70 (0.61–0.81)**	0.047			
No	26	143/1686 (8.5%)	190/1597 (11.9%)	**0.71 (0.54–0.93)**	0.095			
T3 Type of intervention: combination of fluids and vasopressors						0.054	0.231	0.0
Yes	51	419/4620 (9.1%)	571/4674 (12.2%)	**0.74 (0.64–0.86)**	0.045			
No	24	195/1910 (10.2%)	293/1806 (16.2%)	**0.62 (0.48–0.80)**	0.085			
T3 Type of intervention: combination of fluids, vasopressors, and inotropes						0.056	0.686	0.0
Yes	42	249/2564 (9.7%)	352/2462 (14.3%)	**0.68 (0.55–0.85)**	0.092			
No	33	365/3966 (9.2%)	512/4018 (12.7%)	**0.72 (0.62–0.85)**	0.037			
T4 Target variable: reflecting blood flow						0.054	0.248	0.0
Yes	57	497/5123 (9.7%)	715/5131 (13.9%)	**0.69 (0.59–0.81)**	0.077			
No	18	117/1407 (8.3%)	123/1349 (9.1%)	0.81 (0.65–1.01)	0.000			
T5 Target value: personalised						0.053	0.142	0.0
Yes	33	348/3219 (10.8%)	460/3205 (14.4%)	**0.66 (0.65–0.94)**	0.070			
No	42	266/3311 (8.0%)	404/3275 (12.3%)	**0.66 (0.55–0.78)**	0.036			
Adherence to all 5 T's	2	48/177 (27.1%)	56/176 (31.8%)	0.84 (0.62–1.16)	0.000	0.057	0.123	0.0
Type of surgery						0.051	0.198	0.5
Gastrointestinal	33	322/2608 (12.3%)	435/2585 (16.8%)	**0.75 (0.63–0.89)**	0.058			
Cardiothoracic	4	13/325 (4.0%)	32/330 (9.7%)	**0.42 (0.23–0.77)**	0.000			
Other	38	279/3597 (7.8%)	397/3565 (11.1%)	**0.69 (0.56–0.85)**	0.066			
Risk of bias						0.052	0.233	0.0
Low risk of bias	31	307/3119 (9.8%)	441/3129 (14.1%)	**0.70 (0.57–0.87)**	0.107			
CDC Wound classification						0.055	0.252	2.3
I	19	33/1484 (2.2%)	62/1412 (4.4%)	**0.51 (0.34–0.77)**	0.000			
II–III	44	420/3113 (13.5%)	578/3148 (18.3%)	**0.74 (0.63–0.87)**	0.055			
Mixed I–IV	12	161/1933 (8.3%)	224/1920 (11.7%)	**0.70 (0.50–0.97)**	0.131			
Tool used						0.053	0.640	0.0
Non-invasive	24	139/2079 (6.7%)	221/2102 (10.5%)	**0.68 (0.53–0.88)**	0.11			
Invasive	51	475/4451 (10.7%)	643/4378 (14.7%)	**0.73 (0.63–0.84)**	0.04			

Values in bold indicate results that are statistically significant.

τ^2^ (meta-analysis); heterogeneity. τ^2^ (meta-regression): residual heterogeneity after adjusting for subgroup characteristics. ASA: American Society of Anaesthesiologists. CDC: Centres for Disease Control and Prevention. GDHT: goal-directed haemodynamic therapy.

### Secondary outcomes

The results of the secondary outcomes reported in these RCTs can be found in Table 2 and Appendix 5. For three outcomes besides SSI, we found a benefit for GDHT: pneumonia (RR 0.77; 95% CI 0.66–0.90) and patients with at least one complication (RR 0.82; 95% CI 0.75–0.89). The outcomes of mortality, sepsis, AKI, UTI, paralytic ileus, and reoperation were too imprecise to draw conclusions. We found no evidence of harm.

**Table 2:** Secondary outcomes.

	Number of studies	Incidence/total patients in goal-directed haemodynamic therapy group	Incidence/total patients in conventional group	Relative risk (95% confidence interval)	τ^2^ (meta-analysis)
Mortality	41	209/5709 (3.7%)	245/5665 (4.3%)	0.89 (0.74–1.07)	0.000
Sepsis	25	137/3052 (4.5%)	159/3040 (5.2%)	0.95 (0.75–1.19)	0.000
Pneumonia	47	278/5080 (5.5%)	360/4981 (7.2%)	**0.77 (0.66–0.90)**	0.000
Urinary tract infection	28	130/3087 (4.2%)	168/3037 (5.5%)	0.83 (0.67–1.03)	0.018
Acute kidney injury	50	453/5670 (8.0%)	521/5627 (9.3%)	0.86 (0.72–1.02)	0.087
Paralytic ileus	28	224/2558 (8.8%)	257/2546 (10.1%)	0.88 (0.71–1.08)	0.054
Reoperation	30	166/2642 (6.3%)	169/2655 (6.4%)	1.05 (0.86–1.28)	0.016
Number of patients ≥1 complication	52	1563/4333 (36.1%)	1796/4328 (41.5%)	**0.82 (0.75–0.89)**	0.050

Values in bold indicate results that are statistically significant.

### Subgroup analyses of the primary outcome

The subgroup analysis for the 31 studies with low RoB showed a pooled RR of 0.70 (95% CI 0.57–0.87; Table 1, Appendix 6). In the subgroup analysis for adherence to the 5 T's of Saugel and colleagues, the RR increased from a RR of 0.53 with one T present to a RR 0.74 for four T's of appropriate perioperative GDHT (Appendix 7H). Only two studies complied with all five components of the proposed 5 T's framework with an RR of 0.84.^32^^,^^36^ Meta-regression analyses indicated no strong evidence of effect modification by the number of T's complied to or any of the components individually. A bubble plot of the analyses can be found in Appendix 7I. All subgroup analyses and meta-regression are shown in Appendix 7 and Table 1.

Similarly, no strong evidence for an association between the type of surgery and effect size (Appendix 8) or wound contamination and effect size was observed (Appendix 9). TSA showed that the cumulative Z-curve crossed the trial sequential monitoring boundary for benefit (Appendix 10A), indicating that sufficient evidence exists for an at least 25% RR reduction in SSI. This was substantiated in a sensitivity analysis for low RoB studies (Appendix 10B).

### GRADE assessment

The results of the GRADE assessment are shown in Table 3. The starting certainty of the evidence was high as all included studies were RCTs. Overall, no serious RoB was present. Although some RoB was found, the sensitivity analysis results were consistent with the primary analysis (Appendix 6), and we decided not to downgrade for RoB. There was no indirectness as all included studies investigated the same patients (patients undergoing surgery), intervention (GDHT), comparison (no specific fluid management) and outcome (SSI). No inconsistency with τ^2^ = 0.053 and *I*^2^ = 23.5% was found. There was no imprecision as appreciable harm was excluded from the 95% CI, and the optimal information size was met (1388 patients per arm). The contour-enhanced comparison-adjusted funnel plot showed no asymmetry, indicating no publication bias, and resulted in a high-certainty of evidence for the observed effect (Appendix 11).

**Table 3:** Grading of recommendations, assessment, development and evaluation assessment.

Certainty assessment							Number of patients		Effect		Certainty
Number of studies	Study design	Risk of bias^a^	Indirectness	Inconsistency	Imprecision	Other	Surgical site infections /total patients in GDHT group	Surgical site infections /total patients in conventional group	Relative risk (95% confidence interval)	Absolute risk (95% confidence interval)	
75	Randomised controlled trials	Not serious	Not serious	Not serious (*I*^2^ = 23%)	Not serious (OIS 1388 per arm)	None	614/6530 (9.4%)	864/6480 (13.3%)	**0.71** (0.62–0.81)	**39 fewer per 1.000** (from 51 fewer to 25 fewer)	⨁⨁⨁⨁ High

Values in bold indicate results that are statistically significant.

a Appendix 3. Elaborate risk of bias assessment. GDHT: goal-directed haemodynamic therapy. OIS: optimal information size.

## Discussion

This meta-analysis of 75 RCTS, including 13,010 patients, found high-certainty of evidence that GDHT algorithm reduces the risk of SSI after surgery compared to conventional fluid therapy. These results were consistent across subgroup analyses for RoB and patient, procedure, and intervention characteristics. TSA indicated that more RCTs are unlikely to change the direction of effect. Data on other outcomes reported in these RCTs showed that GDHT also helps reduce other postoperative complications without evidence of harm.

Optimising tissue perfusion is essential in perioperative management but challenging to achieve. Hypovolaemia and hypervolaemia are prevalent and associated with postoperative complications.^13^^,^^14^ Algorithms for GDHT have been developed to standardise and optimise fluid management. Several systematic reviews have been conducted on the effect of GDHT on a range of clinical outcomes.^20^^,^^106^, ^107^, ^108^ SSI was identified early on as an important potential benefit of GDHT, but the certainty of evidence was low and led to conditional recommendations.^17^^,^^108^ Since then, the evidence has expanded, with several large RCTs completed in the last few years.^31^^,^^32^^,^^41^^,^^109^ Despite SSI early prominence in GDHT literature, more recent studies focussed on mortality and reported SSI merely as a secondary outcome.^20^^,^^106^ While the limited evidence for the effect on mortality was highlighted, relevant data on SSI were overlooked, and the certainty of evidence on this outcome was underemphasised.^20^^,^^106^ The present systematic review was designed specifically to evaluate the effect of GDHT on SSI. We identified 36 additional RCTs reporting on SSI compared to the most recent systematic review.^20^ Our findings align with earlier reviews and recommendations, but the certainty of this estimate increased markedly.^108^^,^^110^ While some RoB remains, subgroup analysis without potentially biased data indicates that the impact of potential bias on the summary effect is small. Previous concerns about imprecision have been resolved as the increasingly large body of evidence now surpasses the optimal information size. TSA confirmed that additional RCTs are unlikely to change the effect estimate.

Although the wide variation in GDHT algorithms that achieved beneficial results suggests that many different approaches are feasible, few studies failed to achieve a benefit that may result from heterogeneity in patient population and treatment algorithms as described under the 5 T's framework that emphasises adequate target population, timing of intervention, type of intervention, target variable and target value.^1^^,^^2^ We have explored this theory for the first time through meta-regression analyses but did not find strong statistical support for any of the individual components or for every adhered T. Yet, having an algorithm alone does not suffice to obtain improvements in clinical outcomes. Knowledge and training are essential for adequate application of the algorithm.

Notably, the current study did not support the prior notion that GDHT is not effective in low-risk populations according to ASA classification or CDC wound classification; positive effects on SSI incidence were seen across these subgroups. Similarly, evidence that GDHT effects were limited to gastrointestinal surgery or cardiothoracic surgery is also lacking. Additionally, in contrast to previous research,^111^ no difference in mortality rate was observed between GDHT and conventional fluid therapy. This discrepancy may be attributed to the focus of the current meta-analysis on SSI, thus excluding studies that did not report SSI rates. Another potential reason could be the overall decline in mortality rates over the years.

Several limitations to this work should be addressed. The categorisation of high-risk target population was based on the ASA classification. Ideally, we would select high-risk patients based on patient and surgery characteristics. However, the definition of high-risk varied between the studies and were not comparable. Individual patient data would be necessary, but it was not available. The best available alternative is the ASA classification. While imperfect, the ASA classification has been proven to help predict perioperative risks and is a reliable predictor of postoperative complications, irrespective of the surgical procedure.^112^ Admittedly, this does not capture high-risk procedures as they are identified in daily practice. The subgroup analyses presented are explorative and should be interpreted with caution. Although more outcomes have been associated with GDHT, we have focussed this systematic review on SSI. Inference on the estimates of other outcomes is therefore limited. However, the incidence of SSI and corresponding statistical properties ensure that a clear picture of potential effect emerges long before sufficient data on rare events such as mortality are available. Calls for more RCTs to prove other benefits should be weighed cautiously against the potential harms of random allocation of a treatment that already proved effective to help prevent SSI. The major strength of this systematic review and meta-analysis is the robust methodologic quality encompassing the largest data set of patients with SSI included. We implemented standardised frameworks for a comprehensive overview. Extensive analysis provides a complete overview of the updated literature. Previous concerns about imprecision are resolved and likely not to change, while the certainty of the evidence is maximised.

We found high-certainty evidence that goal-directed haemodynamic therapy reduces the risk of SSI when compared to conventional fluid therapy in adults undergoing surgery, and justifies a stronger recommendation for the use of GDHT.

## Contributors

All authors were involved in the conceptualisation of the project, critically reviewed the draft manuscript for important intellectual content, approved the final version and agreed to accountability for all aspects of the work. Funding acquisition was done by MB. HJ and RH accessed and verified the underlying data. Project administration, methodology, data curation, investigation, visualisation, formal analysis and writing of the original draft were done by HJ and RH under the supervision of SJ, MB and MH.

## Data sharing statement

All data is published in this manuscript, the cited manuscripts, or the supplementary appendix. Data can be provided upon request to corresponding authors, and in agreement of terms. No individual participant data was used; we used raw data presented in the cited manuscripts.

## Declaration of interests

RH reports receiving a institutional grant from Amsterdams Universiteitsfonds. MH reports receiving institutional grants, fees, royalties, or payments from ZonMW, European Society of Anaesthesiology and Intensive Care, PAION AG, IDD Pharma, Medical Developments, and Medirisk, and leadership or fiduciary role with Anesthesia & Analgesia, the Journal of Clinical Medicine, and Frontiers in Physiology. MB reports receiving institutional grants, payments, or honoraria from Dutch Association for Quality Funds Medical Specialists, Johnson & Johnson, Solventum, BD, Gore, Angiodynamics, KCI/3 M, TELA Bio, Medtronic, and Smith & Nephew. All other authors declare no competing interests.
