# Supplementary material for: Systematic review and meta-analysis of goal-directed haemodynamic therapy algorithms during surgery for the prevention of surgical site infection
Source: eClinicalMedicine. 2024 Nov 22;78:102944. doi: 10.1016/j.eclinm.2024.102944 (PMC11647171; doi:10.1016/j.eclinm.2024.102944)

**Supplementary appendix - Systematic review and meta-analysis of goal-directed haemodynamic therapy algorithms during surgery for the prevention of surgical site infection**

[**Appendix 1. Search strategy** 2](#_Toc179478471)

[**Appendix 2. Full-text decisions** 5](#_Toc179478472)

[**Appendix 3. Elaborate risk of bias assessment** 11](#_Toc179478473)

[**Appendix 4. Study characteristics** 13](#_Toc179478474)

[**Appendix 5. Forest plots of secondary outcomes** 25](#_Toc179478475)

[5A. Mortality 25](#_Toc179478476)

[5B. Sepsis 26](#_Toc179478477)

[5C. Pneumonia 27](#_Toc179478478)

[5D. Urinary tract infection 28](#_Toc179478479)

[5E. Acute kidney injury 29](#_Toc179478480)

[5F. Paralytic ileus 30](#_Toc179478481)

[5G. Reoperation 31](#_Toc179478482)

[5H. Number of patients with ≥1 complication 32](#_Toc179478483)

[**Appendix 6. Subgroup analysis for the risk of bias: low versus some concerns versus high risk of bias** 33](#_Toc179478484)

[**Appendix 7. Subgroup analyses for the adherence to the 5 T's of Saugel and colleagues** 34](#_Toc179478485)

[7A. Target population 34](#_Toc179478486)

[7B. Timing of intervention 35](#_Toc179478487)

[7C. Type of intervention: fluids and inotropes 36](#_Toc179478488)

[7D. Type of intervention: fluids and vasopressors 37](#_Toc179478489)

[7E. Type of intervention: fluids, inotropes, and vasopressors 38](#_Toc179478490)

[7F. Target variable 39](#_Toc179478491)

[7G. Target value 40](#_Toc179478492)

[7H. Sum of 5T’s of Saugel and colleagues 41](#_Toc179478493)

[7I. Meta-regression bubble plot of the sum of all the 5Ts of Saugel and colleagues 42](#_Toc179478494)

[**Appendix 8. Subgroup analysis for the type of surgery** 43](#_Toc179478495)

[**Appendix 9. Subgroup analysis for the level of wound contamination** 44](#_Toc179478496)

[**Appendix 10. Trial sequential analysis for the primary outcome surgical site infection** 45](#_Toc179478497)

[**Appendix 11. Comparison-adjusted funnel plot** 47](#_Toc179478498)

# **Appendix 1. Search strategy**

**Excerpta Medica Database (Embase.com)**

| No. | Query | Results |
| --- | --- | --- |
| #8 | #4 AND #6 🡪 Randomized Controlled Trials | 1.082 |
| #7 | #4 AND #5 🡪 Systematic Reviews | 267 |
| #6 | 'clinical trial'/exp OR 'randomisation'/exp OR 'single blind procedure'/exp OR 'double blind procedure'/exp OR 'crossover procedure'/exp OR 'placebo'/exp OR 'prospective study'/exp OR rct:ab,ti OR random*:ab,ti OR 'single blind':ab,ti OR 'randomised controlled trial':ab,ti OR 'randomised controlled trial'/exp OR placebo*:ab,ti | 3.319.411 |
| #5 | 'meta analysis'/exp OR 'meta analysis (topic)'/exp OR metaanaly*:ti,ab OR 'meta analy*':ti,ab OR metanaly*:ti,ab OR 'systematic review'/de OR 'cochrane database of systematic reviews'/jt OR prisma:ti,ab OR prospero:ti,ab OR (((systemati* OR scoping OR umbrella OR 'structured literature') NEAR/3 (review* OR overview*)):ti,ab) OR ((systemic* NEAR/1 review*):ti,ab) OR (((systemati* OR literature OR database* OR 'data base*') NEAR/10 search*):ti,ab) OR (((structured OR comprehensive* OR systemic*) NEAR/3 search*):ti,ab) OR (((literature NEAR/3 review*):ti,ab) AND (search*:ti,ab OR database*:ti,ab OR 'data base*':ti,ab)) OR (('data extraction':ti,ab OR 'data source*':ti,ab) AND 'study selection':ti,ab) OR ('search strategy':ti,ab AND 'selection criteria':ti,ab) OR ('data source*':ti,ab AND 'data synthesis':ti,ab) OR medline:ab OR pubmed:ab OR embase:ab OR cochrane:ab OR (((critical OR rapid) NEAR/2 (review* OR overview* OR synthes*)):ti) OR ((((critical* OR rapid*) NEAR/3 (review* OR overview* OR synthes*)):ab) AND (search*:ab OR database*:ab OR 'data base*':ab)) OR metasynthes*:ti,ab OR 'meta synthes*':ti,ab | 733.409 |
| #4 | #3 NOT ('conference abstract'/it OR 'editorial'/it OR 'letter'/it OR 'note'/it) NOT (('animal experiment'/exp OR 'animal model'/exp OR 'nonhuman'/exp) NOT 'human'/exp) AND (dutch:la OR french:la OR german:la OR english:la) | 3250 |
| #3 | #1 AND #2 | 9.887 |
| #2 | 'postoperative infection'/exp OR 'surgical infection'/exp OR ((('post operative' OR postoperative OR surgical) NEAR/3 (infectio* OR complication*)):ti,ab,kw) OR ssi:ti,ab,kw OR ssis:ti,ab,kw | 269.756 |
| #1 | 'haemodynamic monitoring'/exp/mj OR 'lidco':ti,ab,kw OR 'haemodynamic monitoring':ti,ab,kw OR 'haemodynamic monitoring':ti,ab,kw OR 'heart stroke volume'/exp/mj OR 'stroke volume':ti,ab,kw OR 'oxygen delivery'/exp/mj OR 'dobutamine'/exp/mj OR 'butamine':ti,ab,kw OR 'cardiject':ti,ab,kw OR 'dobuject':ti,ab,kw OR 'dobumine':ti,ab,kw OR 'dobutamide':ti,ab,kw OR 'dobutamin*':ti,ab,kw OR 'dobutrex':ti,ab,kw OR 'dobutrexmylan':ti,ab,kw OR 'inotres':ti,ab,kw OR 'inotrex':ti,ab,kw OR 'inotrop':ti,ab,kw OR 'levdobutamine':ti,ab,kw OR 'levo dobutamine':ti,ab,kw OR 'ly 81929':ti,ab,kw OR 'ly81929':ti,ab,kw OR 'oxiken':ti,ab,kw OR 'dopexamine'/exp/mj OR 'dopacard':ti,ab,kw OR 'dopachard':ti,ab,kw OR 'dopexamine':ti,ab,kw OR 'fpl 60278':ti,ab,kw OR 'fpl60278':ti,ab,kw OR 'fluid therapy'/exp/mj OR 'fluid therapy':ti,ab,kw OR 'fluid loading'/exp/mj OR 'oxygen consumption'/exp OR 'o2 consumption':ti,ab,kw OR 'o2 uptake':ti,ab,kw OR 'oxygen consumption':ti,ab,kw OR 'oxygen demand':ti,ab,kw OR 'oxygen intake':ti,ab,kw OR 'oxygen requirement':ti,ab,kw OR 'oxygen uptake':ti,ab,kw OR 'oxygen utilization':ti,ab,kw OR 'cardiac index'/exp/mj OR 'cardiac index':ti,ab,kw OR 'heart index':ti,ab,kw OR 'heart output'/exp/mj OR 'cardiac minute volume':ti,ab,kw OR 'cardiac output':ti,ab,kw OR 'cardiac ventricle output':ti,ab,kw OR 'decreased cardiac output':ti,ab,kw OR 'heart minute volume':ti,ab,kw OR 'heart output':ti,ab,kw OR 'heart ventricle output':ti,ab,kw OR 'blood volume'/exp/mj OR 'blood volume':ti,ab,kw OR 'normovolaemia':ti,ab,kw OR 'normovolemia':ti,ab,kw OR 'goal directed therapy'/exp OR 'goal directed fluid therapy'/exp OR 'heart volume'/exp/mj OR 'cardiac volume':ti,ab,kw OR 'heart volume':ti,ab,kw OR 'heart function'/exp/mj OR 'cardiac function':ti,ab,kw OR 'heart function':ti,ab,kw OR 'heart muscle function':ti,ab,kw OR 'myocardial function':ti,ab,kw OR 'myocardium function':ti,ab,kw OR ((fluid NEAR/3 (load* OR administration OR management OR challenge)):ti,ab,kw) OR 'oxygen delivery':ti,ab,kw OR supranormal:ti,ab,kw OR supronormal:ti,ab,kw OR (((haemodynamic OR haemodynamic) NEAR/3 (intervention OR therapy)):ti,ab,kw) OR ((goal NEAR/3 (directed OR target* OR oriented)):ti,ab,kw) | 1.029.599 |

**Ovid/MEDLINE**

| **#** | **Searches** | **Results** |
| --- | --- | --- |
| 8 | 4 and 6 🡪 Randomized Controlled Trials | 779 |
| 7 | 4 and 5 🡪 Systematic Reviews | 243 |
| 6 | (exp clinical trial/ or randomised controlled trial/ or exp clinical trials as topic/ or randomised controlled trials as topic/ or Random Allocation/ or Double-Blind Method/ or Single-Blind Method/ or (clinical trial, phase i or clinical trial, phase ii or clinical trial, phase iii or clinical trial, phase iv or controlled clinical trial or randomised controlled trial or multicenter study or clinical trial).pt. or random*.ti,ab. or (clinic* adj trial*).tw. or ((singl* or doubl* or treb* or tripl*) adj (blind$3 or mask$3)).tw. or Placebos/ or placebo*.tw.) not (animals/ not humans/) | 2.567.439 |
| 5 | (meta-analysis/ or meta-analysis as topic/ or (metaanaly* or meta-analy* or metanaly*).ti,ab,kf. or systematic review/ or cochrane.jw. or (prisma or prospero).ti,ab,kf. or ((systemati* or scoping or umbrella or "structured literature") adj3 (review* or overview*)).ti,ab,kf. or (systemic* adj1 review*).ti,ab,kf. or ((systemati* or literature or database* or data-base*) adj10 search*).ti,ab,kf. or ((structured or comprehensive* or systemic*) adj3 search*).ti,ab,kf. or ((literature adj3 review*) and (search* or database* or data-base*)).ti,ab,kf. or (("data extraction" or "data source*") and "study selection").ti,ab,kf. or ("search strategy" and "selection criteria").ti,ab,kf. or ("data source*" and "data synthesis").ti,ab,kf. or (medline or pubmed or embase or cochrane).ab. or ((critical or rapid) adj2 (review* or overview* or synthes*)).ti. or (((critical* or rapid*) adj3 (review* or overview* or synthes*)) and (search* or database* or data-base*)).ab. or (metasynthes* or meta-synthes*).ti,ab,kf.) not (comment/ or editorial/ or letter/ or ((exp animals/ or exp models, animal/) not humans/)) | 45.8429 |
| 4 | 3 not ((exp animals/ or exp models, animal/) not humans/) not (letter/ or comment/ or editorial/) | 3.120 |
| 3 | 1 and 2 | 3.239 |
| 2 | Haemodynamic Monitoring/ or lidco.ti,ab,kf. or haemodynamic monitoring.ti,ab,kf. or haemodynamic monitoring.ti,ab,kf. or Cardiac Output/ or stroke volume.ti,ab,kf. or Dobutamine/ or butamine.ti,ab,kf. or cardiject.ti,ab,kf. or dobuject.ti,ab,kf. or dobumine.ti,ab,kf. or dobutamide.ti,ab,kf. or dobutamin*.ti,ab,kf. or dobutrex.ti,ab,kf. or dobutrexmylan.ti,ab,kf. or inotres.ti,ab,kf. or inotrex.ti,ab,kf. or inotrop.ti,ab,kf. or levdobutamine.ti,ab,kf. or levo dobutamine.ti,ab,kf. or ly 81929.ti,ab,kf. or ly81929.ti,ab,kf. or oxiken.ti,ab,kf. or dopacard.ti,ab,kf. or dopachard.ti,ab,kf. or dopexamine.ti,ab,kf. or fpl 60278.ti,ab,kf. or fpl60278.ti,ab,kf. or exp Fluid Therapy/ or fluid therapy.ti,ab,kf. or Oxygen Consumption/ or o2 consumption.ti,ab,kf. or o2 uptake.ti,ab,kf. or oxygen consumption.ti,ab,kf. or oxygen demand.ti,ab,kf. or oxygen intake.ti,ab,kf. or oxygen requirement.ti,ab,kf. or oxygen uptake.ti,ab,kf. or oxygen utilisation.ti,ab,kf. or cardiac index.ti,ab,kf. or heart index.ti,ab,kf. or cardiac minute volume.ti,ab,kf. or cardiac output.ti,ab,kf. or cardiac ventricle output.ti,ab,kf. or decreased cardiac output.ti,ab,kf. or heart minute volume.ti,ab,kf. or heart output.ti,ab,kf. or heart ventricle output.ti,ab,kf. or exp Blood Volume/ or blood volume.ti,ab,kf. or normovolaemia.ti,ab,kf. or normovolemia.ti,ab,kf. or exp Cardiac Volume/ or cardiac volume.ti,ab,kf. or heart volume.ti,ab,kf. or cardiac function.ti,ab,kf. or heart function.ti,ab,kf. or heart muscle function.ti,ab,kf. or myocardial function.ti,ab,kf. or myocardium function.ti,ab,kf. or (fluid adj3 (load* or administration or management or challenge)).ti,ab,kf. or oxygen delivery.ti,ab,kf. or supranormal.ti,ab,kf. or supronormal.ti,ab,kf. or ((haemodynamic or haemodynamic) adj3 (intervention or therapy)).ti,ab,kf. or (goal adj3 (directed or target* or oriented)).ti,ab,kf. | 389850 |
| 1 | exp surgical wound infection/ or (('post operative' or postoperative or surg*) adj5 (infection* or complication*)).ti,ab,kf. or ssi.ti,ab,kf. or ssis.ti,ab,kf. | 238.368 |

**Cochrane Library**

| #1 | MeSH deschriptor: [Hemondynaming Monitoring] explode all trees | 29 |
| --- | --- | --- |
| #2 | MeSH deschriptor: [Cardiac Output] explode all trees | 7498 |
| #3 | MeSH deschriptor: [Dobutamine] explode all trees | 643 |
| #4 | MeSH deschriptor:[Oxygen Consumption] explode all trees | 8444 |
| #5 | MeSH deschriptor:[Blood Volume] explode all trees | 1381 |
| #6 | MeSH deschriptor:[Cardiac Volume] explode all trees | 205 |
| #7 | 'lidco':ti,ab,kw OR 'haemodynamic monitoring':ti,ab,kw OR 'hemodynamic monitoring':ti,ab,kw OR 'stroke volume':ti,ab,kw OR 'butamine':ti,ab,kw OR 'cardiject':ti,ab,kw OR 'dobuject':ti,ab,kw OR 'dobumine':ti,ab,kw OR 'dobutamide':ti,ab,kw OR 'dobutamin*':ti,ab,kw OR 'dobutrex':ti,ab,kw OR 'dobutrexmylan':ti,ab,kw OR 'inotres':ti,ab,kw OR 'inotrex':ti,ab,kw OR 'inotrop':ti,ab,kw OR 'levdobutamine':ti,ab,kw OR 'levo dobutamine':ti,ab,kw OR 'ly 81929':ti,ab,kw OR 'ly81929':ti,ab,kw OR 'oxiken':ti,ab,kw OR 'dopacard':ti,ab,kw OR 'dopachard':ti,ab,kw OR 'dopexamine':ti,ab,kw OR 'fpl 60278':ti,ab,kw OR 'fpl60278':ti,ab,kw OR 'fluid therapy':ti,ab,kw OR 'o2 consumption':ti,ab,kw OR 'o2 uptake':ti,ab,kw OR 'oxygen consumption':ti,ab,kw OR 'oxygen demand':ti,ab,kw OR 'oxygen intake':ti,ab,kw OR 'oxygen requirement':ti,ab,kw OR 'oxygen uptake':ti,ab,kw OR 'oxygen utilization':ti,ab,kw OR 'cardiac index':ti,ab,kw OR 'heart index':ti,ab,kw OR 'cardiac minute volume':ti,ab,kw OR 'cardiac output':ti,ab,kw OR 'cardiac ventricle output':ti,ab,kw OR 'decreased cardiac output':ti,ab,kw OR 'heart minute volume':ti,ab,kw OR 'heart output':ti,ab,kw OR 'heart ventricle output':ti,ab,kw OR 'blood volume':ti,ab,kw OR 'normovolaemia':ti,ab,kw OR 'normovolemia':ti,ab,kw OR 'cardiac volume':ti,ab,kw OR 'heart volume':ti,ab,kw OR 'cardiac function':ti,ab,kw OR 'heart function':ti,ab,kw OR 'heart muscle function':ti,ab,kw OR 'myocardial function':ti,ab,kw OR 'myocardium function':ti,ab,kw OR ((fluid NEAR/3 (load* OR administration OR management OR challenge)):ti,ab,kw) OR 'oxygen delivery':ti,ab,kw OR supranormal:ti,ab,kw OR supronormal:ti,ab,kw OR (((hemodynamic OR haemodynamic) NEAR/3 (intervention OR therapy)):ti,ab,kw) OR ((goal NEAR/3 (directed OR target* OR oriented)):ti,ab,kw) |  |
| #8 | #1 OR #2 OR #3 OR #4 OR #5 OR #6 OR #7 | 147152 |
| #9 | MeSH descriptor: [Surgical Wound Infection] explode all trees | 4623 |
| #10 | ((('post operative' OR postoperative OR surgical) NEAR/3 (infectio* OR complication*)):ti,ab,kw) OR ssi:ti,ab,kw OR ssis:ti,ab,kw | 66528 |
| #11 | #9 OR #10 | 66528 |
| #12 | #8 AND #11 | 7021 |

The search was conducted from inception of the databases up to September 12, 2024

# **Appendix 2. Full-text decisions**

|  | **Author, Year** | **Reason for exclusion** |
| --- | --- | --- |
| **1** | Feng 2023^1^ | Outcome not of interest |
| **2** | Hrdy 2023^2^ | Outcome not of interest |
| **3** | Ji 2023^3^ | Postoperative GDHT |
| **4** | Ma 2023^4^ | Outcome not of interest |
| **5** | Schaller 2023^5^ | Outcome not of interest |
| **6** | Chui 2022^6^ | Outcome not of interest |
| **7** | Froghi 2022^7^ | Postoperative GDHT |
| **8** | Hokenek 2022^8^ | Outcome not of interest |
| **9** | Hokenek 2022^8^ | Duplicate |
| **10** | Mishra 2022^9^ | Duplicate |
| **11** | Turkut 2022^10^ | Outcome not of interest |
| **12** | Wongtangman 2022^11^ | Outcome not of interest |
| **13** | Wongtangman 2021^11^ | Duplicate |
| **14** | Bloria 2021^12^ | Outcome not of interest |
| **15** | Cho 2021^13^ | Outcome not of interest |
| **16** | Li 2021^14^ | Outcome not of interest |
| **17** | Liu 2021^15^ | Comparison not of interest |
| **18** | Omar 2021^16^ | Outcome not of interest |
| **19** | Taylor 2021^17^ | Outcome not of interest |
| **20** | Tribuddharat 2021^18^ | Outcome not of interest |
| **21** | Wang 2021^19^ | Outcome not of interest |
| **22** | Chui 2020^20^ | Outcome not of interest |
| **23** | Fischer 2020^21^ | Outcome not of interest |
| **24** | Martin 2020^22^ | Postoperative GDHT |
| **25** | Cesur 2019^23^ | Outcome not of interest |
| **26** | Coeckelenbergh 2019^24^ | Comparison not of interest |
| **27** | Bahlmann 2018^25^ | Comparison not of interest |
| **28** | Calvo-Vecino 2018^26^ | Duplicate |
| **29** | Gerent 2018^27^ | Postoperative GDHT |
| **30** | Liu 2018^28^ | Outcome not of interest |
| **31** | Myles 2018^29^ | Comparison not of interest |
| **32** | Yin 2018^30^ | Outcome not of interest |
| **33** | Kaufmann 2017^31^ | Outcome not of interest |
| **34** | Liang 2017^32^ | Outcome not of interest |
| **35** | Xu 2017^33^ | Outcome not of interest |
| **36** | Broch 2016^34^ | Outcome not of interest |
| **37** | Deschamps 2016^35^ | Comparison not of interest |
| **38** | Kassim 2016^36^ | Comparison not of interest |
| **39** | Li 2016^37^ | Not available through library |
| **40** | Schmid 2016^38^ | Outcome not of interest |
| **41** | Ackland 2015^39^ | Outcome not of interest |
| **42** | Benes 2015^40^ | Comparison not of interest |
| **43** | Correa-Gallego 2015^41^ | Comparison not of interest |
| **44** | Funk 2015^42^ | Outcome not of interest |
| **45** | Jammer 2015^43^ | Outcome not of interest |
| **46** | Lai 2015^44^ | Outcome not of interest |
| **47** | Mikor 2015^45^ | Outcome not of interest |
| **48** | Cowie 2014^46^ | Comparison not of interest |
| **49** | Zeng 2014^47^ | Retracted |
| **50** | Chattopadhyay 2013^48^ | No randomisation |
| **51** | Goepfert 2013^49^ | Outcome not of interest |
| **52** | Jones 2013^50^ | Postoperative GDHT |
| **53** | Salzwedel 2013^51^ | Outcome not of interest |
| **54** | Challand 2012^52^ | Outcome not of interest |
| **55** | Cohn 2010^53^ | Comparison not of interest |
| **56** | Rhodes 2010^54^ | Outcome not of interest |
| **57** | Van Der Linden 2010^55^ | Outcome not of interest |
| **58** | Senagore 2009^56^ | Comparison not of interest |
| **59** | Harten 2008^57^ | Outcome not of interest |
| **60** | Chytra 2007^58^ | Postoperative GDHT |
| **61** | Donati 2007^59^ | Outcome not of interest |
| **62** | Murkin 2007^60^ | Comparison not of interest |
| **63** | Lobo 2006^61^ | Comparison not of interest |
| **64** | Noblett 2006^62^ | Outcome not of interest |
| **65** | Pearse 2005^63^ | Outcome not of interest |
| **66** | Pearse 2005^63^ | Duplicate |
| **67** | McKendry 2004^64^ | Postoperative GDHT |
| **68** | Conway 2002^65^ | Outcome not of interest |
| **69** | Ueno 1998^66^ | Postoperative GDHT |
| **70** | Bender 1997^67^ | Comparison not of interest |
| **71** | Sincleair 1997^68^ | Postoperative GDHT |
| **72** | Boyd 1993^69^ | Comparison not of interest |
| **73** | Joyce 1990^70^ | Comparison not of interest |
| **74** | Bar 2024^71^ | Outcome not of interest |
| **75** | Jenko 2024^72^ | Outcome not of interest |
| **76** | Li 2024^73^ | Comparison not of interest |
| **77** | Wu 2024^74^ | Outcome not of interest |
| **78** | Chaimala 2023^75^ | Not available through library |
| **79** | Feng 2023^76^ | Outcome not of interest |
| **80** | Göçmen 2023^77^ | Duplicate |
| **81** | Hrdy 2023^78^ | Outcome not of interest |
| **82** | Ji 2023^79^ | Comparison not of interest |
| **83** | Ma 2023^80^ | Outcome not of interest |
| **84** | Mahrose 2023^81^ | Duplicate |
| **85** | Sea-Phua 2023^82^ | Outcome not of interest |
| **86** | Vu 2023^83^ | Duplicate |
| **87** | Wang 2023^84^ | Duplicate |
| 1. Feng A, Lu P, Yang Y, Liu Y, Ma L, Lv J. Effect of goal-directed fluid therapy based on plasma colloid osmotic pressure on the postoperative pulmonary complications of older patients undergoing major abdominal surgery. World J Surg Oncol. 2023;21(1):67. Published 2023 Feb 28. doi:10.1186/s12957-023-02955-5.  2. Hrdy O, Duba M, Dolezelova A, et al. Effects of goal-directed fluid management guided by a non-invasive device on the incidence of postoperative complications in neurosurgery: a pilot and feasibility randomised controlled trial. Perioper Med (Lond). 2023;12(1):32. Published 2023 Jul 5. doi:10.1186/s13741-023-00321-3.  3. Ji J, Ma Q, Tian Y, et al. Effect of inferior vena cava respiratory variability-guided fluid therapy after laparoscopic hepatectomy: a randomised controlled clinical trial. Chin Med J (Engl). 2023;136(13):1566-1572. Published 2023 Jul 5. doi:10.1097/CM9.0000000000002484.  4. Ma H, Li X, Wang Z, et al. The effect of intraoperative goal-directed fluid therapy combined with enhanced recovery after surgery program on postoperative complications in elderly patients undergoing thoracoscopic pulmonary resection: a prospective randomised controlled study. Perioper Med (Lond). 2023;12(1):33. Published 2023 Jul 10. doi:10.1186/s13741-023-00327-x.  5. Schaller SJ, Fuest K, Ulm B, et al. Goal-directed Perioperative Albumin Substitution Versus Standard of Care to Reduce Postoperative Complications - A Randomised Clinical Trial (SuperAdd Trial) [published online ahead of print, 2023 Jul 21]. Ann Surg. 2023;10.1097/SLA.0000000000006030. doi:10.1097/SLA.0000000000006030.  6. Chui J, Craen R, Dy-Valdez C, et al. Early Goal-directed Therapy During Endovascular Coiling Procedures Following Aneurysmal Subarachnoid Hemorrhage: A Pilot Prospective Randomized Controlled Study. J Neurosurg Anesthesiol. 2022;34(1):35-43. doi:10.1097/ANA.0000000000000700.  7. Froghi F, Gopalan V, Anastasiou Z, Koti R, Gurusamy K, Eastgate C, et al. Effect of postoperative goal-directed fluid therapy (GDHT) on organ function after orthotopic liver transplantation: Secondary outcome analysis of the COLT randomised control trial. Int J Surg. 2022;99:106265.  8. Hokenek UD, Gurler HK, Saracoglu A, Kale A, Saracoglu KT. Pleth Variability Index Guided Volume Optimisation in Major Gynaecologic Surgery. J Coll Physicians Surg Pak. 2022;32(8):980-6.  9. Mishra N, Rath GP, Bithal PK, Chaturvedi A, Chandra PS, Borkar SA. Effect of Goal-Directed Intraoperative Fluid Therapy on Duration of Hospital Stay and Postoperative Complications in Patients Undergoing Excision of Large Supratentorial Tumors. Neurol India. 2022;70(1):108-114. doi:10.4103/0028-3886.336329.  10. Turkut N, Altun D, Canbolat N, Uzuntürk C, Şen C, Çamcı AE. Comparison of Stroke Volume Variation-based goal-directed Therapy Versus Standard Fluid Therapy in Patients Undergoing Head and Neck Surgery: A Randomized Controlled Study. Balkan Med J. 2022;39(5):351-357. doi:10.4274/balkanmedj.galenos.2022.2022-1-88.  11. Wongtangman K, Wilartratsami S, Hemtanon N, Tiviraj S, Raksakietisak M. Goal-Directed Fluid Therapy Based on Pulse-Pressure Variation Compared with Standard Fluid Therapy in Patients Undergoing Complex Spine Surgery: A Randomized Controlled Trial. Asian Spine J. 2022;16(3):352-60.  12. Bloria SD, Panda NB, Jangra K, Bhagat H, Mandal B, Kataria K, et al. Goal-directed Fluid Therapy Versus Conventional Fluid Therapy During Craniotomy and Clipping of Cerebral Aneurysm: A Prospective Randomized Controlled Trial. J Neurosurg Anesthesiol. 2022;34(4):407-14.  13. Cho HJ, Huang YH, Poon KS, Chen KB, Liao KH. Perioperative haemodynamic optimisation in laparoscopic sleeve gastrectomy using stroke volume variation to reduce postoperative nausea and vomiting. Surg Obes Relat Dis. 2021;17(9):1549-57.  14. Li M, Peng M. Prospective comparison of the effects of intraoperative goal-directed fluid therapy and restrictive fluid therapy on complications in thoracoscopic lobectomy. J Int Med Res. 2021;49(12):3000605211062787.  15. Liu Y, Chen G, Gao J, Chi M, Mao M, Shi Y, et al. Effect of different levels of stroke volume variation on the endothelial glycocalyx of patients undergoing colorectal surgery: A randomised clinical trial. Exp Physiol. 2021;106(10):2124-32.  16. Omar IH, Okasha AS, Ahmed AM, Saleh RS. Goal Directed Fluid Therapy based on Stroke Volume Variation and Oxygen Delivery Index using Electrical Cardiometry in patients undergoing Scoliosis Surgery. Egyptian Journal of Anaesthesia. 2021;37(1):241-7.  17. Taylor RJ, Patel R, Wolf BJ, Stoll WD, Hornig JD, Skoner JM, et al. Intraoperative vasopressors in head and neck free flap reconstruction. Microsurgery. 2021;41(1):5-13.  18. Tribuddharat S, Sathitkarnmanee T, Ngamsangsirisup K, Nongnuang K. Efficacy of Intraoperative Haemodynamic Optimization Using FloTrac/EV1000 Platform for Early Goal-Directed Therapy to Improve Postoperative Outcomes in Patients Undergoing Coronary Artery Bypass Graft with Cardiopulmonary Bypass: A Randomized Controlled Trial. Med Devices (Auckl). 2021;14:201-9.  19. Wang X, Duan Y, Gao Z, Gu J. Effect of Goal-directed Fluid Therapy on the Shedding of the Glycocalyx Layer in Retroperitoneal Tumour Resection. J Coll Physicians Surg Pak. 2021;31(10):1179-85.  20. Chui J, Craen R, Dy-Valdez C, Alamri R, Boulton M, Pandey S, et al. Early Goal-directed Therapy During Endovascular Coiling Procedures Following Aneurysmal Subarachnoid Hemorrhage: A Pilot Prospective Randomized Controlled Study. J Neurosurg Anesthesiol. 2022;34(1):35-43.  21. Fischer MO, Lemoine S, Tavernier B, Bouchakour CE, Colas V, Houard M, et al. Individualised Fluid Management Using the Pleth Variability Index: A Randomised Clinical Trial. Anesthesiology. 2020;133(1):31-40.  22. Martin D, Koti R, Gurusamy K, Longworth L, Singh J, Froghi F, et al. The cardiac output optimisation following liver transplant (COLT) trial: a feasibility randomised controlled trial. HPB (Oxford). 2020;22(8):1112-20.  23. Cesur S, Cardakozu T, Kus A, Turkyilmaz N, Yavuz O. Comparison of conventional fluid management with PVI-based goal-directed fluid management in elective colorectal surgery. J Clin Monit Comput. 2019;33(2):249-57.  24. Coeckelenbergh S, Delaporte A, Ghoundiwal D, Bidgoli J, Fils JF, Schmartz D, et al. Pleth variability index versus pulse pressure variation for intraoperative goal-directed fluid therapy in patients undergoing low-to-moderate risk abdominal surgery: a randomised controlled trial. BMC Anesthesiol. 2019;19(1):34.  25. Bahlmann H, Hahn RG, Nilsson L. Pleth variability index or stroke volume optimisation during open abdominal surgery: a randomised controlled trial. BMC Anesthesiol. 2018;18(1):115.  26. Calvo-Vecino JM, Ripolles-Melchor J, Mythen MG, Casans-Frances R, Balik A, Artacho JP, et al. Effect of goal-directed haemodynamic therapy on postoperative complications in low-moderate risk surgical patients: a multicentre randomised controlled trial (FEDORA trial). Br J Anaesth. 2018;120(4):734-44.  27. Gerent ARM, Almeida JP, Fominskiy E, Landoni G, de Oliveira GQ, Rizk SI, et al. Effect of postoperative goal-directed therapy in cancer patients undergoing high-risk surgery: a randomised clinical trial and meta-analysis. Crit Care. 2018;22(1):133.  28. Liu TJ, Zhang JC, Gao XZ, Tan ZB, Wang JJ, Zhang PP, et al. Clinical research of goal-directed fluid therapy in elderly patients with radical resection of bladder cancer. J Cancer Res Ther. 2018;14(Supplement):S173-S9.  29. Myles PS, Bellomo R, Corcoran T, Forbes A, Peyton P, Story D, et al. Restrictive versus Liberal Fluid Therapy for Major Abdominal Surgery. N Engl J Med. 2018;378(24):2263-74.  30. Yin K, Ding J, Wu Y, Peng M. Goal-directed fluid therapy based on non-invasive cardiac output monitor reduces postoperative complications in elderly patients after gastrointestinal surgery: A randomised controlled trial. Pak J Med Sci. 2018;34(6):1320-5.  31. Kaufmann KB, Stein L, Bogatyreva L, Ulbrich F, Kaifi JT, Hauschke D, et al. Oesophageal Doppler guided goal-directed haemodynamic therapy in thoracic surgery - a single centre randomised parallel-arm trial. Br J Anaesth. 2017;118(6):852-61.  32. Liang M, Li Y, Lin L, Lin X, Wu X, Gao Y, et al. Effect of goal-directed fluid therapy on the prognosis of elderly patients with hypertension receiving plasmakinetic energy transurethral resection of prostate. Int J Clin Exp Med. 2017;10(1):1290-6.  33. Xu H, Shu SH, Wang D, Chai XQ, Xie YH, Zhou WD. Goal-directed fluid restriction using stroke volume variation and cardiac index during one-lung ventilation: a randomised controlled trial. J Thorac Dis. 2017;9(9):2992-3004.  34. Broch O, Carstens A, Gruenewald M, Nischelsky E, Vellmer L, Bein B, et al. Non-invasive haemodynamic optimisation in major abdominal surgery: a feasibility study. Minerva Anestesiol. 2016;82(11):1158-69.  35. Deschamps A, Hall R, Grocott H, Mazer CD, Choi PT, Turgeon AF, et al. Cerebral Oximetry Monitoring to Maintain Normal Cerebral Oxygen Saturation during High-risk Cardiac Surgery: A Randomized Controlled Feasibility Trial. Anesthesiology. 2016;124(4):826-36.  36. Kassim DY, Esmat IM. Goal directed fluid therapy reduces major complications in elective surgery for abdominal aortic aneurysm: Liberal versus restrictive strategies. Egyptian Journal of Anaesthesia. 2016;32(2):167-73.  37. Li S, Ma Q, Yang Y, Lu J, Zhang Z, Jin M, et al. Novel Goal-Directed Haemodynamic Optimization Therapy Based on Major Vasopressor during Corrective Cardiac Surgery in Patients with Severe Pulmonary Arterial Hypertension: A Pilot Study. Heart Surg Forum. 2016;19(6):E297-E302.  38. Schmid S, Kapfer B, Heim M, Bogdanski R, Anetsberger A, Blobner M, et al. Algorithm-guided goal-directed haemodynamic therapy does not improve renal function after major abdominal surgery compared to good standard clinical care: a prospective randomised trial. Crit Care. 2016;20:50.  39. Ackland GL, Iqbal S, Paredes LG, Toner A, Lyness C, Jenkins N, et al. Individualised oxygen delivery targeted haemodynamic therapy in high-risk surgical patients: a multicentre, randomised, double-blind, controlled, mechanistic trial. Lancet Respir Med. 2015;3(1):33-41.  40. Benes J, Haidingerova L, Pouska J, Stepanik J, Stenglova A, Zatloukal J, et al. Fluid management guided by a continuous non-invasive arterial pressure device is associated with decreased postoperative morbidity after total knee and hip replacement. BMC Anesthesiol. 2015;15:148.  41. Correa-Gallego C, Tan KS, Arslan-Carlon V, Gonen M, Denis SC, Langdon-Embry L, et al. Goal-Directed Fluid Therapy Using Stroke Volume Variation for Resuscitation after Low Central Venous Pressure-Assisted Liver Resection: A Randomised Clinical Trial. J Am Coll Surg. 2015;221(2):591-601.  42. Funk DJ, HayGlass KT, Koulack J, Harding G, Boyd A, Brinkman R. A randomised controlled trial on the effects of goal-directed therapy on the inflammatory response open abdominal aortic aneurysm repair. Crit Care. 2015;19(1):247.  43. Jammer I, Tuovila M, Ulvik A. Stroke volume variation to guide fluid therapy: is it suitable for high-risk surgical patients? A terminated randomised controlled trial. Perioper Med (Lond). 2015;4:6.  44. Lai CW, Starkie T, Creanor S, Struthers RA, Portch D, Erasmus PD, et al. Randomised controlled trial of stroke volume optimisation during elective major abdominal surgery in patients stratified by aerobic fitness. Br J Anaesth. 2015;115(4):578-89.  45. Mikor A, Trasy D, Nemeth MF, Osztroluczki A, Kocsi S, Kovacs I, et al. Continuous central venous oxygen saturation assisted intraoperative haemodynamic management during major abdominal surgery: a randomised, controlled trial. BMC Anesthesiol. 2015;15:82.  46. Cowie DA, Nazareth J, Story DA. Cerebral oximetry to reduce perioperative morbidity. Anaesth Intensive Care. 2014;42(3):310-4.  47. Zeng K, Li Y, Liang M, Gao Y, Cai H, Lin C. The influence of goal-directed fluid therapy on the prognosis of elderly patients with hypertension and gastric cancer surgery. Drug Des Devel Ther. 2014;8:2113-9.  48. Chattopadhyay S, Mittal S, Christian S, Terblanche AL, Patel A, Biliatis I, et al. The role of intraoperative fluid optimisation using the esophageal Doppler in advanced gynecological cancer: early postoperative recovery and fitness for discharge. Int J Gynecol Cancer. 2013;23(1):199-207.  49. Goepfert MS, Richter HP, Zu Eulenburg C, Gruetzmacher J, Rafflenbeul E, Roeher K, et al. Individually optimised haemodynamic therapy reduces complications and length of stay in the intensive care unit: a prospective, randomised controlled trial. Anesthesiology. 2013;119(4):824-36.  50. Jones C, Kelliher L, Dickinson M, Riga A, Worthington T, Scott MJ, et al. Randomised clinical trial on enhanced recovery versus standard care following open liver resection. Br J Surg. 2013;100(8):1015-24.  51. Salzwedel C, Puig J, Carstens A, Bein B, Molnar Z, Kiss K, et al. Perioperative goal-directed haemodynamic therapy based on radial arterial pulse pressure variation and continuous cardiac index trending reduces postoperative complications after major abdominal surgery: a multi-center, prospective, randomised study. Crit Care. 2013;17(5):R191.  52. Challand C, Struthers R, Sneyd JR, Erasmus PD, Mellor N, Hosie KB, et al. Randomised controlled trial of intraoperative goal-directed fluid therapy in aerobically fit and unfit patients having major colorectal surgery. Br J Anaesth. 2012;108(1):53-62.  53. Cohn SM, Pearl RG, Acosta SM, Nowlin MU, Hernandez A, Guta C, et al. A prospective randomised pilot study of near-infrared spectroscopy-directed restricted fluid therapy versus standard fluid therapy in patients undergoing elective colorectal surgery. Am Surg. 2010;76(12):1384-92.  54. Rhodes A, Cecconi M, Hamilton M, Poloniecki J, Woods J, Boyd O, et al. Goal-directed therapy in high-risk surgical patients: a 15-year follow-up study. Intensive Care Med. 2010;36(8):1327-32.  55. Van der Linden PJ, Dierick A, Wilmin S, Bellens B, De Hert SG. A randomised controlled trial comparing an intraoperative goal-directed strategy with routine clinical practice in patients undergoing peripheral arterial surgery. Eur J Anaesthesiol. 2010;27(9):788-93.  56. Senagore AJ, Emery T, Luchtefeld M, Kim D, Dujovny N, Hoedema R. Fluid management for laparoscopic colectomy: a prospective, randomised assessment of goal-directed administration of balanced salt solution or hetastarch coupled with an enhanced recovery program. Dis Colon Rectum. 2009;52(12):1935-40.  57. Harten J, Crozier JE, McCreath B, Hay A, McMillan DC, McArdle CS, et al. Effect of intraoperative fluid optimisation on renal function in patients undergoing emergency abdominal surgery: a randomised controlled pilot study (ISRCTN 11799696). Int J Surg. 2008;6(3):197-204.  58. Chytra I, Pradl R, Bosman R, Pelnar P, Kasal E, Zidkova A. Esophageal Doppler-guided fluid management decreases blood lactate levels in multiple-trauma patients: a randomised controlled trial. Crit Care. 2007;11(1):R24.  59. Donati A, Loggi S, Preiser JC, Orsetti G, Munch C, Gabbanelli V, et al. Goal-directed intraoperative therapy reduces morbidity and length of hospital stay in high-risk surgical patients. Chest. 2007;132(6):1817-24.  60. Murkin J, Adams S. novick RJ, Quantz M, Bainbridge D, iglesias i, Cleland A, Schaefer B, irwin B, Fox S: Monitoring brain oxygen saturation during coronary bypass surgery: A randomised, prospective study. Anesth Analg. 2007;104:51-8.  61. Lobo SM, Lobo FR, Polachini CA, Patini DS, Yamamoto AE, de Oliveira NE, et al. Prospective, randomised trial comparing fluids and dobutamine optimisation of oxygen delivery in high-risk surgical patients [ISRCTN42445141]. Crit Care. 2006;10(3):R72.  62. Noblett SE, Snowden CP, Shenton BK, Horgan AF. Randomised clinical trial assessing the effect of Doppler-optimised fluid management on outcome after elective colorectal resection. Br J Surg. 2006;93(9):1069-76.  63. Pearse R, Dawson D, Fawcett J, Rhodes A, Grounds RM, Bennett ED. Early goal-directed therapy after major surgery reduces complications and duration of hospital stay. A randomised, controlled trial [ISRCTN38797445]. Crit Care. 2005;9(6):R687-93.  64. McKendry M, McGloin H, Saberi D, Caudwell L, Brady AR, Singer M. Randomised controlled trial assessing the impact of a nurse delivered, flow monitored protocol for optimisation of circulatory status after cardiac surgery. BMJ. 2004;329(7460):258.  65. Conway DH, Mayall R, Abdul-Latif MS, Gilligan S, Tackaberry C. Randomised controlled trial investigating the influence of intravenous fluid titration using oesophageal Doppler monitoring during bowel surgery. Anaesthesia. 2002;57(9):845-9.  66. Ueno S, Tanabe G, Yamada H, Kusano C, Yoshidome S, Nuruki K, et al. Response of patients with cirrhosis who have undergone partial hepatectomy to treatment aimed at achieving supranormal oxygen delivery and consumption. Surgery. 1998;123(3):278-86.  67. Bender JS, Smith-Meek MA, Jones CE. Routine pulmonary artery catheterisation does not reduce morbidity and mortality of elective vascular surgery: results of a prospective, randomised trial. Ann Surg. 1997;226(3):229-36; discussion 36-7.  68. Sinclair S, James S, Singer M. Intraoperative intravascular volume optimisation and length of hospital stay after repair of proximal femoral fracture: randomised controlled trial. BMJ. 1997;315(7113):909-12.  69. Boyd O, Grounds RM, Bennett ED. A randomised clinical trial of the effect of deliberate perioperative increase of oxygen delivery on mortality in high-risk surgical patients. JAMA. 1993;270(22):2699-707.  70. Joyce WP, Provan JL, Ameli FM, McEwan MM, Jelenich S, Jones DP. The role of central haemodynamic monitoring in abdominal aortic surgery. A prospective randomised study. Eur J Vasc Surg. 1990;4(6):633-6.  71. Bar S, Moussa MD, Descamps R, et al. Determinants of postoperative complications in high-risk noncardiac surgery patients optimized with hemodynamic treatment strategies: A post-hoc analysis of a randomized multicenter clinical trial. J Clin Anesth. 2024;93:111325.  72. Jenko M, Mencin K, Novak-Jankovic V, Spindler-Vesel A. Influence of different intraoperative fluid management on postoperative outcome after abdominal tumours resection. Radiol Oncol. 2024;58(2):279-288.  73. Li S, Yin Y, Wang P, Jiang L, Yan H, Cang J. Goal-directed fluid therapy during post-resection phase in low central venous pressure assisted laparoscopic hepatectomy: a randomized controlled superiority trial. J Anesth. 2024;38(1):77-85.  74. Wu B, Guo Y, Min S, Xiong Q, Zou L. Postoperative cognitive dysfunction in elderly patients with colorectal cancer: A randomized controlled study comparing goal-directed and conventional fluid therapy. Open Med (Wars). 2024;19(1):20240930.  75. Chaimala N, Tribuddharat S, Sathitkarnmanee T, Sinkueakunkit A. Early Goal-Directed Therapy Using FloTrac/EV1000 Platform for Hemodynamic Optimization to Improve Perioperative Outcomes in Patients Undergoing Major Abdominal Surgery: A Randomized Controlled Trial. Journal of the Medical Association of Thailand. 2023 Oct 1;106(10).  76. Feng A, Lu P, Yang Y, Liu Y, Ma L, Lv J. Effect of goal-directed fluid therapy based on plasma colloid osmotic pressure on the postoperative pulmonary complications of older patients undergoing major abdominal surgery. World J Surg Oncol. 2023;21(1):67.  77. Göçmen D, Köksal C, Abitağaoğlu S, Yildirim Ar A. Comparison of the effects of intraoperative goal directed and conventional fluid management on the inferior vena cava collapsibility index and postoperative complications in geriatric patients operated from proximal femoral nail surgery. Turkish Journal of Geriatrics/Türk Geriatri Dergisi. 2023 Jan 1;26(1).  78. Hrdy O, Duba M, Dolezelova A, et al. Effects of goal-directed fluid management guided by a non-invasive device on the incidence of postoperative complications in neurosurgery: a pilot and feasibility randomized controlled trial. Perioper Med (Lond). 2023;12(1):32.  79. Ji J, Ma Q, Tian Y, et al. Effect of inferior vena cava respiratory variability-guided fluid therapy after laparoscopic hepatectomy: a randomized controlled clinical trial. Chin Med J (Engl). 2023;136(13):1566-1572.  80. Ma H, Li X, Wang Z, et al. The effect of intraoperative goal-directed fluid therapy combined with enhanced recovery after surgery program on postoperative complications in elderly patients undergoing thoracoscopic pulmonary resection: a prospective randomized controlled study. Perioper Med (Lond). 2023;12(1):33.  81. Mahrose R, Kasem AA. Pulse Pressure Variation-Based Intraoperative Fluid Management Versus Traditional Fluid Management for Colon Cancer Patients Undergoing Open Mass Resection and Anastomosis: A Randomized Controlled Trial. Anesth Pain Med. 2023;13(4):e135659.  82. Sae-Phua V, Tanasittiboon S, Sangtongjaraskul S. The Effect of Goal-directed Fluid Management based on Stroke Volume Variation on ICU Length of Stay in Elderly Patients Undergoing Elective Craniotomy: A Randomized Controlled Trial. Indian J Crit Care Med. 2023;27(10):709-716.  83. Vu PH, Duong ND, Tran VD, Tran TH, Luu XV, Truong QT, Nguyen HT, Ochiai R. Effectiveness of goal-directed fluid therapy guided by estimated continuous cardiac output (esCCO) in major gastrointestinal surgeries: a randomized controlled trial. Anaesthesia, Pain & Intensive Care. 2023 Jun 7;27(3):371-8.  84. Vu PH, Duong ND, Tran VD, Tran TH, Luu XV, Truong QT, Nguyen HT, Ochiai R. Effectiveness of goal-directed fluid therapy guided by estimated continuous cardiac output (esCCO) in major gastrointestinal surgeries: a randomized controlled trial. Anaesthesia, Pain & Intensive Care. 2023 Jun 7;27(3):371-8. | | |

# **Appendix 3. Elaborate risk of bias assessment**

**
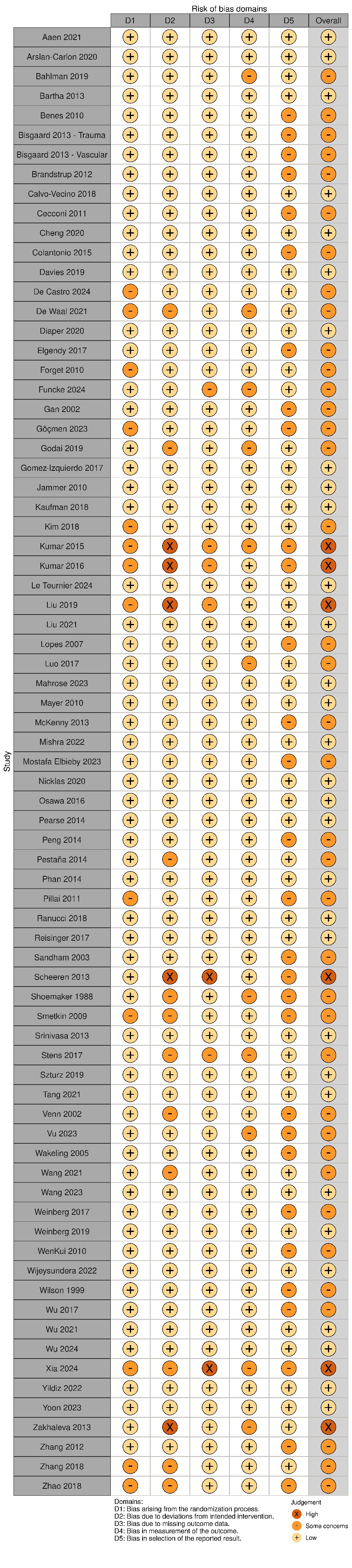
**


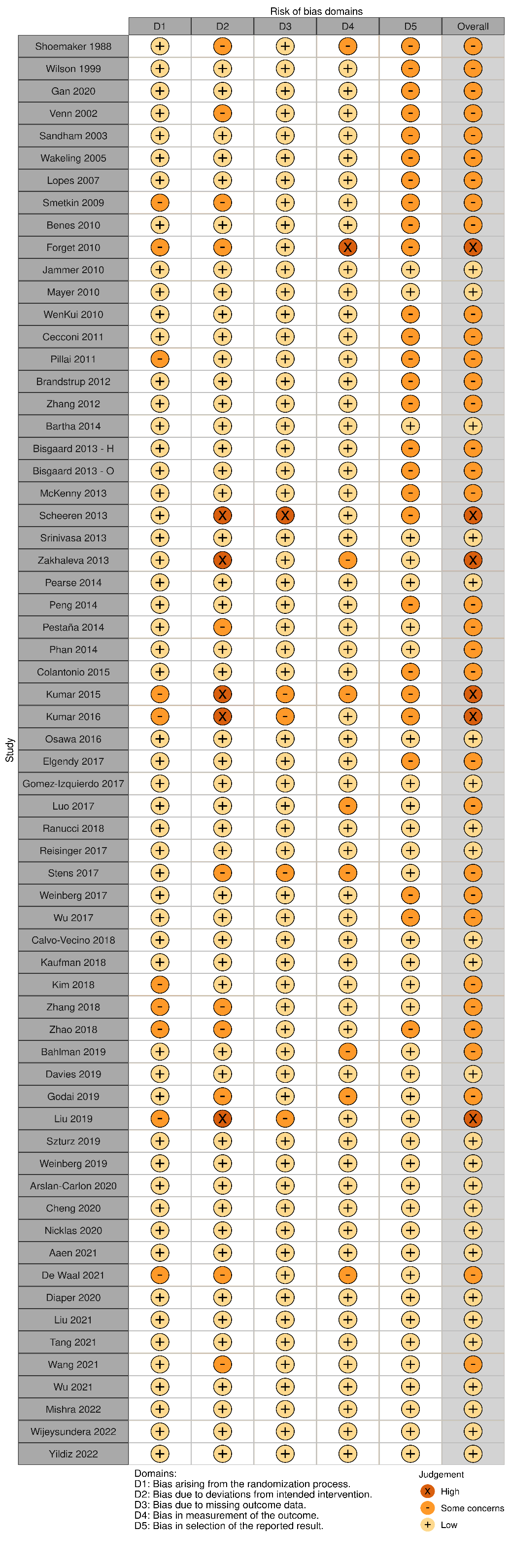
 High risk of bias

Some concerns

Low risk of bias

**
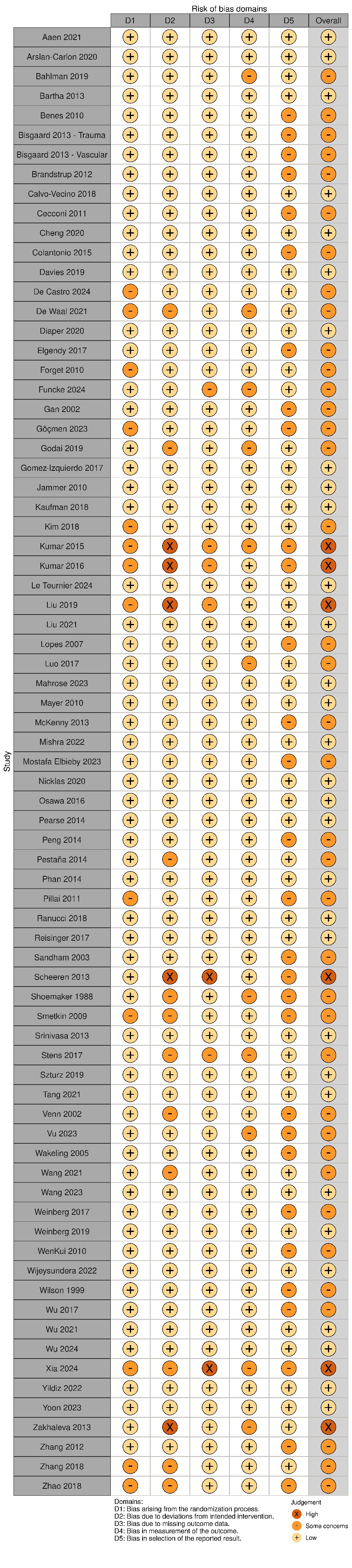
**

Domains:

D1 Bias arising from the randomisation process

D2 Bias due to deviations from the intended intervention

D3 Bias due to missing outcome data

D4 Bias in the measurement of the outcome

D5 Bias in the selection of the reported result

This risk of bias plot was created using the ROBVIS tool

McGuinness, LA, Higgins, JPT. Risk-of-bias VISualization (robvis): An R package and Shiny web app for visualizing risk-of-bias assessments. Res Syn Meth. 2020; 1- 7. https://doi.org/10.1002/jrsm.1411

# **Appendix 4. Study characteristics**

| **Study** | **Type of surgery** | **Goal-directed haemodynamic therapy** | | **Conventional fluid therapy** | | **Risk of Bias** | **Definition SSI** |
| --- | --- | --- | --- | --- | --- | --- | --- |
|  |  | **Tools and goals** | **Intervention** | **Tools and goals** | **Intervention** |  |  |
| Aaen 2021^1^ | Emergency surgery for obstructive bowel disease or GI perforation | Arterial line (FloTrac/EV1000) | Colloids | - | Crystalloids, colloids, vasopressors | Low | Superficial wound infection: Wound rupture, surgical revision or medical treatment |
|  |  | SV increase <10%, MAP >65 mmHg | Vasopressors | MAP >65 mmHg, UO >0.5 ml/kg/h | Vasopressors |  |  |
| Arslan-Carlon 2020^2^ | Open radical cystectomy | Arterial line (FloTrac/Vigileo) | Crystalloids, colloids | - | Colloids, RBC | Low | NI |
|  |  | SV increase ≤10%, SVV <8% MAP ≥60 mmHg, CI 2.5 | Inotropes, vasoconstrictors | Hb >7 mg/dl | Ephedrine, phenylephrine |  |  |
| Bahlmann 2019^3^ | Open thoracotomy | Arterial line (FloTrac/Vigileo) | Crystalloids, colloids, fresh frozen plasma, RBC | - | Colloids | SC | Superficial SSI: Local inflammatory signs and specific antibiotic treatment |
|  |  | SV <10%, CI ≥2.5 l/min/m^2^, MAP >65 mmHg | Dobutamine, phenylephrine, norepinephrine | Discretion anaesthetist  (Hb >90/100 g/l) | Dobutamine, phenylephrine, norepinephrine |  |  |
| Bartha 2014^4^ | Proximal femoral fracture surgery | Arterial line (LiDCO) | Colloids | - | Crystalloids | Low | Deep and superficial with purulent exudate and treated by antibiotic |
|  |  | SV <10% increase, DO_2_I ≥600 ml/min, SBP decrease <30% | Dobutamine | SBP decrease <30% | Vasoactive substances |  |  |
| Benes 2010^5^ | Major abdominal surgery >120 min (colorectal or pancreatic resections, intra-abdominal vascular surgery) | Arterial line (FloTrac/Vigileo) | Colloid | - | Crystalloids, colloids | SC | CDC |
|  |  | SVV <10% , CVP >15 mmHg, CI 2.5-4.0 l/min/m^2^, SAP >90 mmHg, MAP >65 mmHg | Dobutamine, ephedrine, norepinephrine | MAP >65 mmHg, HR<100 bpm, CVP 8-15 mmHg, UO>0.5 ml/kg/h | Vasoactive substances |  |  |
| Bisgaard 2013 – Trauma^6^ | Open elective lower limb arterial surgery | Arterial line (LiDCO) | Colloids | Arterial line | Fluid therapy | SC | NI |
|  |  | SVI increase >10%, DO_2_I <600 ml/min/m^2^, HR <100 or <20% above baseline, acute hypotension | Phenylephrine, ephedrine, | MAP 60–100 mmHg, SaO_2_ > 94%, Hb ≥ 9.3 g/l, Temp > 36.5°C, HR <100 or <20% above baseline | Phenylephrine, ephedrine |  |  |
| Bisgaard 2013 – Vascular^7^ | Open elective abdominal aortic surgery | Arterial line (LiDCOplus) | Colloids | Arterial line | Colloids, RBCs | SC | NI |
|  |  | SVI increase >10%, DO_2_I <600 ml/min/m^2^, MAP >65 mmHg or >70% of baseline | Phenylephrine, ephedrine | MAP >65 mmHg or >70%  of baseline | Phenylephrine, ephedrine, dobutamine |  |  |
| Brandstrup 2012^8^ | Colorectal resection, open and laparoscopic | Oesophageal Doppler (CardioQ) | Colloids | - | Colloids | SC | NI |
|  |  | SV increase <10% | Phenylephrine, ephedrine | MAP >60 mmHg | Phenylephrine, ephedrine |  |  |
| Calvo-Vecino 2018^9^ | Abdominal, urological, gynaecological, orthopaedic surgery  (estimated duration ≥2h,  blood loss >15%, or ≥2 packs RBC) | Oesophageal Doppler (CardioQ) | Crystalloids, colloids | - | Discretion anaesthetist: crystalloids, colloids | Low | *Presumably CDC* |
|  |  | SV <10% increase, MAP >65 mmHg, CI >2.5 l/min/m^2^ | Vasopressors, inotropes | Avoid extremes of clinical practice misalignment | Discretion anaesthetist: dobutamine, norepinephrine |  |  |
| Cecconi 2011^10^ | Total hip arthroplasty | Arterial line (FloTrac/Vigileo) | Crystalloids, colloids | - | Colloids | SC | ¶ |
|  |  | SV increase <10%, DO2I ≥600ml/min/m^2^ | Dobutamine | Discretion anaesthetist  MAP >65 mmHg | Ephedrine |  |  |
| Cheng 2020^11^ | Cardiac surgery (valvular surgery and coronary artery bypass grafting with CPB) | Arterial line (LiDCOrapid), INVOS, BIS | Crystalloids, RBCs | INVOS, BIS | Crystalloids, RBCs | Low | NI |
|  |  | rScO_2_ decline <20%, MAP <20% decline, CI ≥2 l/min/m^2^, BIS 40-45/45-60 | Norepinephrine, dobutamine, propofol | HR 60-100 bpm,  ScVO2 >70%, lactate <3 mmol/L, Ht >28%, UO >0.5 mL/kg/h, MAP = 65mmHg | Norepinephrine, dobutamine |  |  |
| Colantonio 2015^12^ | Open peritonectomy and HIPEC | Arterial line (FloTrac/Vigileo) | Colloids | CVC, arterial line | Colloids | SC | NI |
|  |  | CI ≥2.5 l/min/m^2^, SVI ≥35ml/m^2^, SVV <15% | Dobutamine | MAP 65-90 mmHg or >70% pre-induction, CVP >15 mmHg, diuresis >1 ml/kg/h | Dopamine |  |  |
| Davies 2019^13^ | Emergency proximal femoral fracture surgery | Finger cuff (Clearsight system) | Colloids | - | Crystalloids | Low | Wound infection:  Deep and/or superficial with purulent exudate and treated by antibiotics |
|  |  | SVV <10%, MAP within 30% of baseline | Phenylephrine, metaraminol | Discretion anaesthetist | Inotropes, vasopressors |  |  |
| De Castro 2024 ^14^ | Elective medium or major open abdominal surgeries (sigmoidectomy, colec- tomy, hemicolectomy, splenectomy, recto-sigmoidectomy, pancreatectomy, intestinal transit recon-struction, abdomino-perineal amputation, biliary bypass, gastric-tomy, exploratory laparotomy, partial hepatectomy, gastro-pancreatoduodenectomy | NI, *non-invasive* | Crystalloids, colloids | NI, *non-invasive* | Discretion anaesthetist |  | NI |
|  |  | MAP 65−80 mmHg, dPP ≤12% | Vasoactive drugs | MAP 65−80 mmHg, dPP ≤12% | Discretion anaesthetist |  |  |

| De Waal 2021^15^ | High-risk abdominal surgery  (esophagectomy, pancreaticoduode- nectomy, open abdominal aortic aneurysm repair, major abdominal resections for soft tissue malignancy) | Arterial line (FloTrac/Vigileo) | Crystalloids, colloids | Arterial and central venous line | Crystalloids, colloids | SC | NI |
| --- | --- | --- | --- | --- | --- | --- | --- |
|  |  | CI ≥ 2.8/2.6/2.4 or <10% increase l min/m^2^, SVV <12% | Inotropes | HR <100 min or ≤25% above individual baseline MAP  ≥ 60 mmHg or ≤25% below individual baseline, saturation ≥ 95% | Vasopressors |  |  |
| Diaper 2021^16^ | Major abdominal, urological, or vascular surgery via open laparotomy (≥2h) | Arterial line (LiDCO) | Crystalloids, colloids | Arterial line (LiDCO) | Crystalloids, colloids | Low | CD ≥2 |
|  |  | SVI <10%, MAP decrease <20% or >65-70 mmHg, PPV <10% | Ephedrine, phenylephrine, norepinephrine | MAP >65-70 mmHg, fluid losses, hypovolemia, metabolic acidosis | Ephedrine, phenylephrine, noradrenaline |  |  |
| Elgendy 2017^17^ | Major abdominal surgery (duration >120 min or blood loss >20%) | Arterial line (FloTrac/Vigileo) | Colloids | - | Colloids | SC | NI |
|  |  | SVV <12%, CI >2.5, MAP >65mmHg | Dobutamine, norepinephrine | 60 and 90 mmHg with CVP in range of 8–12 mmHg  and UO of >0.5 ml/kg/h | Dobutamine, norepinephrine |  |  |
| Forget 2010^18^ | Gastrointestinal hepato-biliary surgery | Pulse oximeter (Masimo) | Colloids | - | Colloids | SC | NI |
|  |  | PVI <13%, MAP >65 mmHg | Norepinephrine | Blood loss <50 ml, MAP >65 mmHg, CVP >6 mmHg | Norepinephrine |  |  |
| Funcke 2024^19^ | Elective major  open abdominal (i.e. visceral, urological, gynaecological;  duration >2h, >2L IV fluids, >10% risk of postoperative  complications | Arterial line (ProAQT) | Crystalloids, colloids | Discretion anaesthetist:  Arterial line, CVL | Discretion anaesthetist:  Fluids |  | Wound infection: divided in superficial, deep, organ/space without further definition |
|  |  | PPV < 12%, CI 2.5 l/min/m^2^  HR <100 bpm, MAP >65 mmHg, Temp > 36C | Vasopressors, dobutamine | HR <100 bpm, MAP >65 mmHg, SpO2 > 94%, core temp > 36C | Discretion anaesthetist: Vasopressors, catecholamines |  |  |
| Gan 2002^20^ | Major elective general, urologic, and gynaecologic surgery (blood loss >500 ml) | Oesophageal Doppler | Colloids | - | Fluids | SC | NI |
|  |  | FTc >350 ms, SV increase <10% | - | UO >0.5 ml/kg/h, HR <120% baseline or <110 bpm, SBP decrease <20% baseline or >90 mmHg, CVP decrease <20% of baseline | - |  |  |
| Göçmen 2023^21^ | Proximal femoral nail surgery | Arterial line (MostcarTM monitor) | Colloids, crystalloids | - | Crystalloids, colloids | SC | NI |
|  |  | SVV<13%, PPV<10%, CI>2.5L/min/m^2^, MAP>65 mmHg | Norepinephrine | According to ‘4-2-1 formula’ | - |  |  |
| Godai 2019^22^ | Elective open gynaecological surgery | Arterial line (FloTrac/Vigileo), Life Scope J | Colloids | Life Scope J | Discretion anaesthetist | SC | Postop complications, including infection of organs and leakage at the anastomosis site |
|  |  | MAP <60 mmHg, PPV ≤13%, pulse index increasing in 15 min | Phenylephrine, dobutamine | MAP >60 mmHg | Discretion anaesthetist |  |  |
| Gómez-Izquierdo 2017^23^ | Laparoscopic colorectal surgery | Oesophageal Doppler | Colloids | - | Discretion anaesthetist | Low | CDC |
|  |  | SV <10% | Discretion anaesthetist | Discretion anaesthetist | Discretion anaesthetist |  |  |
| Jammer 2010^24^ | Elective open colorectal and lower intestinal  surgery | Central venous line | Colloids | - | Crystalloids | Low | ¤ |
|  |  | ScvO2 >75%, MAP >60 mmHg | - | - | - |  |  |
| Kaufmann 2018^25^ | Hip arthroplasty | Oesophageal Doppler (Deltex) | Crystalloids, RBC | - | Crystalloids | Low | Wound healing disorder: drainage of pus from the surgical wound |
|  |  | MAP >70 mmHg, SV increase <10%, CI >2.5 l/min/m^2^ | Norepinephrine, ephedrine | MAP >60 mmHg or <20% decrease from baseline, UO >0.5 ml/kg/h, HR <20% increase from baseline | Norepinephrine, dobutamine, epinephrine, ephedrine |  |  |
| Kim 2018^26^ | Free flap reconstruction | Arterial line (FloTrac) | Colloids | - | Crystalloids | SC | NI |
|  |  | SVV <12%, CI ≥2.5 l/min/m^2^, MAP ≥65 mmHg, HR <100 bpm | Dobutamine, ephedrine, norepinephrine | MAP >65 mmHg, UO >0.5ml/kg/h | Ephedrine, norepinephrine |  |  |
| Kumar 2016^27^ | Major abdominal surgery (pancreaticoduode-nectomy, low anterior resection, retroperitoneal tumour resection, gastrectomy) | Arterial line (FloTrac/Vigileo) | Crystalloids, colloids | - | Discretion anaesthetist | High | NI |
|  |  | SVV <10% | - | CVP 10-12 mmHg | - |  |  |
| Kumar 2015^28^ | ‘High-risk’ (patient or surgery-related factors) abdominal surgery | Arterial line (FloTrac/Vigileo) | Crystalloids, colloids | - | Crystalloids, colloids | High | NI |
|  |  | CI ≥2.5 L/min, O_2_ER ≤ 27%, MAP 65 mmHg, SVV <10% | Dopamine, dobutamine, norepinephrine | MAP ≥65 mmHg, CVP ≥8–10 mmHg, UO ≥0.5 mL/kg/h, ScVO_2_ ≥70% | Dobutamine, noradrenaline |  |  |
| Le Teurnier 2024^29^ | Carotid endarterectomy | Near-infrared spectroscopy (INVOS 5100C cerebral/somatic oximeter), Arterial line | Colloids | Arterial line | Fluids | Low | NI |
|  |  | rSO_2_ >85%, SBP > 20% baseline, MAP <110 mmHg | Ephedrine, phenylephrine | SBP >10%–20% baseline | Ephedrine, phenylephrine, norepinephrine |  |  |
| Liu 2021^30^ | Elective open gastrointestinal surgery (duration >2h) | Arterial line (Vigileo/FloTrac) | Colloids | - | Discretion anaesthetist: crystalloids, colloids | Low | SSI: clinical diagnosis  Intra-abdominal infection: CT-confirmed |
|  |  | SVV <12%, CI ≥2.5 l/min/m^2^,  MAP >65 mmHg | Inotropes, vasoactive agents | HR <100 bpm, MAP >65 mmHg, UO >0.5ml/kg/h | Discretion anaesthetist: vasoactive agents |  |  |
| Liu 2019^31^ | Laparoscopic colorectal surgery | Arterial line (FloTrac/Vigileo) | Colloids | - | Crystalloids, colloids | High | NI |
|  |  | SVV <13%, CI >2.5 l/min/m^2^ | Dobutamine | UO > 5-10 ml/kg/h, SBP and HR variation <20% | Dobutamine, norepinephrine, nitroglycerin |  |  |
| Lopes 2007^32^ | ‘High-risk’ surgery (gastrointestinal, hepatobiliary, urology) | IBPplus/DX 2020 | Colloids | - | - | SC | NI |
|  |  | ΔPP ≤10% | - | Discretion of anaesthetist | - |  |  |
| Luo 2017^33^ | Elective craniotomy (duration >2h) | Arterial line (FloTrac/Vigileo) | Colloids | - | Discretion anaesthetist and intensivist | SC | NI |
|  |  | MAP ≥65 mmHg, CI ≥2.5 l/min/m^2^, SVV <15% | Vasopressors | Discretion anaesthetist and intensivist | Discretion anaesthetist and intensivist |  |  |
| Mahrose 2023^34^ | Elective open resection colon cancer mass with anastomosis | Arterial line (CARESCAPETM B650) | Colloids | Central venous catheter (Certofix) | Crystalloids, colloids | Low | NI |
|  |  | PPV <12%, MAP >65 mmHg | Ephedrine | MAP <0-30% baseline or >65 mmHg | Ephedrine |  |  |
| Mayer 2010^35^ | Open major abdominal surgery (pancreaticoduo-denectomy, intestine, gastric liver, or oesophageal resection) | Arterial line (FloTrac/Vigileo) | Crystalloids, Colloids | - | Crystalloids, colloids | Low | Wound infection: clinical diagnosis. Abdominal infection: Abdominal CT |
|  |  | CI >2.5L/min/m^2^, MAP >65mmHg, SVI >35mL/m^2^ | Norepinephrine, dobutamine | MAP 65-90 mmHg, CVP 8-12 mmHg, UO >0.5 mL/kg/h | - |  |  |
| McKenny 2013^36^ | Open excision of malignancy of the uterus and adnexa | Oesophageal Doppler | Colloids | Arterial line | Discretion anaesthetist | SC | Infection: on antibiotics or Temperature >38 °C in last 24h |
|  |  | SV increase <10% | - | UO >0.5ml/kg/h, HR, SBP, CVP, blood loss | Discretion anaesthetist |  |  |
| Mishra 2022^37^ | Neurosurgery (excision supratentorial tumours ≥4 cm) | Arterial line (FloTrac/Vigileo) | Crystalloids, colloids | - | Colloids | Low | NI |
|  |  | SVV <12%, CI > 2.5L/min/m^2^, MAP >60 mmHg, | Dobutamine, ephedrine | MAP 65-90 mmHg, CVP 8-12 mmHg, UO >0.5 ml/kg/h | Vasoactive agents |  |  |
| Mostafa Elbieby 2023^38^ | Colorectal surgery | Non-invasive electrical  cardiometry | Crystalloids, colloids | - | Crystalloids, colloids, RBC’s | SC | NI |
|  |  | SVV 8-10%, MAP >65 mmHg | Vasoactive agents | MAP >80% baseline or >65 mmHg, Hb 10 g/dl | Vasoactive agents |  |  |
| Nicklas 2020^39^ | Major abdominal surgery (≥90 min or blood loss >1000 ml) | Arterial line (ProAQT) | Colloids | Arterial line | Discretion anaesthetist: crystalloids, colloids | Low | NI |
|  |  | CI ≥ personal baseline, MAP 65-90 mmHg, HR <120 BPM | Dobutamine, phenylephrine, norepinephrine | MAP >65 mmHg | Discretion anaesthetist: norepinephrine |  |  |
| Osawa 2016^40^ | Cardiac surgery (coronary artery bypass grafting (CABG) and valvular surgery) | Arterial line (LiDCOrapid) | Crystalloids, RBCs | - | Crystalloids, RBCs | Low | CDC |
|  |  | CI >3L/min/m^2^, SVI >35ml/m^2^, Ht >28%, CVP increase >4% | Dobutamine | HR 70-100 bpm, Svco2 >70%, lactate <3 mmol/L, Ht >28%, UO >0.5 mL/kg/h | Dobutamine |  |  |
| Pearse 2014^41^ | Major gastrointestinal surgery (>90 min, high-risk patient criteria) | Arterial line (LiDCOrapid) | Colloids | CVC | - | Low | CDC |
|  |  | SV <10% increase, HR <120% baseline or <100 bpm | Dopexamine | ‘Usual perioperative care’ (dynamic central venous pressure target) | - |  |  |
| Peng 2014^42^ | Elective major orthopaedic surgery (blood loss >800 ml) | Arterial line (FloTrac/Vigileo) | Colloid, RBCs | CVC, arterial line | Crystalloids, colloids, RBCs | SC | NI |
|  |  | SVV increase <10/14%, MAP >65 mmHg, Hb >10 g/l | Ephedrine | MAP >65 mmHg, HR <100 bpm, CVP 8–14 mmHg, UO >0.5 ml/kg/h, Hb >10 g/l | Ephedrine |  |  |
| Pestaña 2014^43^ | Open colorectal surgery, gastrectomy, small bowel resection | NICOM | Colloids |  | Discretion anaesthetist and ICU specialist | SC | Pus expressed from the incision or aspired from mass within the wound |
|  |  | MAP ≥65 mmHg, CI ≥2.5 L/min/m^2^ | Dobutamine, norepinephrine | ‘Standard of care’ | Discretion anaesthetist and ICU specialist |  |  |
| Phan 2014^44^ | Elective open or laparoscopic major colorectal surgery | Oesophageal Doppler | Colloids | - | Crystalloids | Low | NI |
|  |  | SVI >35ms/m2, %, FTc <360 msec | - | Replace blood loss or hypotension, not  responsive to vasopressor | Vasopressors |  |  |
| Pillai 2011^45^ | Open radical cystectomy | Oesophageal Doppler | Colloids | - | Fluids | SC | NI |
|  |  | SV increase <10%, FTc >350ms | - | Discretion anaesthetist | - |  |  |
| Ranucci 2018^46^ | Cardiopulmonary bypass (≥90 min) | NI | RBCs, increasing pump flow | - | - | Low | NI |
|  |  | DO_2_ >280 mL/min/m^2^, SvO_2_ >68%, oxygen extraction rate <40% | - | Arterial pump flow 2.4 L/min/m^2^ at normothermia.  Local standards | - |  |  |
| Reisinger 2017^47^ | Elective open or laparoscopic colorectal surgery | Oesophageal Doppler | Crystalloids, colloids | - | Crystalloids, colloids | Low | NI |
|  |  | SVI increase <10% | Vasoactive drugs, inotropic drugs | Standard haemodynamic and clinical parameters | Vasoactive drugs, inotropic drugs |  |  |
| Sandham 2003^48^ | Major surgery (abdominal, thoracic, vascular, orthopaedic) | Pulmonary-artery catheter | Fluids, RBCs | Central venous catheter | - | SC | CDC |
|  |  | DO_2_I 550-600 ml/min/m^2^ body-surface area, CI 3.5-4.5 L/m^2^, MAP >70 mmHg, PCWP 18 mmHg, HR <120 bpm, Ht >27% | Inotropes, vasodilator therapy, vasopressors | Central venous pressure | - |  |  |
| Scheeren 2013^49^ | ‘High-risk’ surgery with postoperative ICU care | Arterial line (FloTrac/Vigileo) | Colloids | - | - | High | NI |
|  |  | SVV <10%, SV decrease <10% | - | Standardised protocol | - |  |  |
| Shoemaker 1988^50^ | Mixed surgery in high-risk patients | Pulmonary artery catheter (CO computer Model 9520) | Crystalloids, colloids, RBCs | Pulmonary artery catheter or CVP | Crystalloids, colloids, RBCs | SC | NI |
|  |  | CO >4.5 L/min/m^2^, DO_2_ >600 ml/min/m^2^, VO_2_ >170 ml/min/m^2^ | Inotropes, vasodilators, vasopressors | Normative standards of pulmonary arterial pressures, HR, MAP, CVP, pulmonary capillary wedge pressure | Inotropes, vasodilators, vasopressors |  |  |
| Smetkin 2009^51^ | Coronary surgery (off-Pump Coronary Artery Bypass Grafting) | Arterial line (PiCCOplus/CeVOX) | Colloids, RBCs | Arterial line | Colloids | SC | NI |
|  |  | ITBVI 850-1000 ml/m_2_, CI >2L/min/m^2^, HR <90 bpm, MAP 60-90 mmHg, ScvO_2_ >60% | Nitroglycerine, furosemide, verapamil, phenylephrine, ephedrine, dobutamine | CVP 6-14 mmHg, HR <90 bpm, MAP 60-100 mmHg | Nitroglycerine, furosemide, dobutamine, verapamil, phenylephrine, ephedrine |  |  |
| Srinivasa 2013^52^ | Elective open or laparoscopic colectomy | Oesophageal Doppler | Colloids | Arterial line (optional) | Crystalloids, colloids | Low | NI |
|  |  | FTc >350ms, SV increase <10% | Discretion anaesthetist:  vasopressor | HR, BP, UO | Discretion anaesthetist:  vasopressor |  |  |
| Stens 2017^53^ | Moderate-risk abdominal surgery (grade-2 risk Modified Johns Hopkins Surgical Criteria) | Finger arterial blood pressure (ccNexfin device) | Crystalloids, colloids | - | Discretion anaesthetist | SC | † |
|  |  | MAP >70 mmHg, CI >2.5 l/min/m^2^ PPV <12% | Dobutamine, norepinephrine | MAP >70 mmHg | Discretion anaesthetist: vasopressors, inotropes |  |  |
| Szturz 2019^54^ | Open gastrointestinal surgery (>120 min or blood loss >15%) | Oesophageal Doppler (CardioQ) | Crystalloids |  | Discretion anaesthetist | Low | NI |
|  |  | CI 2.5-3.8 l/min/m^2^, FTc <330ms, PV <70/m, systemic vascular resistance index1000-18000 cdyn.s.cm^-5^.m^-2^ | Dobutamine, norepinephrine, isosorbide dinitrate | Based on HR, MAP, CVP,  diuresis, clinical findings | Discretion anaesthetist |  |  |
| Tang 2021^55^ | Minimally invasive oesophagectomy | Arterial line (PiCCO, Pulsion) | Colloids |  | Crystalloids, colloids | Low | NI |
|  |  | SVV <9% | - | According to ‘4-2-1 formula’ |  |  |  |
| Venn 2002^56^ | Hip fracture surgery | Oesophageal Doppler | Crystalloids, colloids | CVC | Colloids | SC | NI |
|  |  | Flow time <0.4s, SV increase <10% |  | CVP >14 mmHg | - |  |  |
| Vu 2023^57^ | Major gastrointestinal surgery | esCCO monitor | Crystalloids | - | Crystalloids | SC | NI |
|  |  | esSVI ≥10%, esCCI >2.5 l/min/m2, | Dobutamine, ephedrine, phenylephrine | SBP > 90 mmHg or decrease < 20% of baseline, CV <5 cm cmH2O | Ephedrine or phenylephrine |  |  |
| Wakeling 2005^58^ | (Semi-)elective large bowel surgery | Oesophageal Doppler | Colloids | Routine cardiovascular monitoring | Routine fluid management | SC | Ω |
|  |  | SV <10%, CVP <3 mmHg | - | CVP 12-15 mmHg | - |  |  |
| Wang 2021^59^ | Spine surgery | Arterial line (InfinityC700) | Crystalloids | - | Crystalloids | SC | NI |
|  |  | PPV <14%, SV increase <10%, MAP >60 mmHg | Ephedrine, norepinephrine | MAP >60 mmHg or SBP >90 mmHg, | Ephedrine, norepinephrine |  |  |
| Wang 2023^60^ | Major gastrointestinal surgery (gastrectomy,  small bowel resection, colorectal surgery) | Finger sensor (Rainbow R2-25a) | Crystalloids, colloids | CVC | Crystalloids, colloids | Low | *Presumably CDC* |
|  |  | MAP >65 mmHg, PVI <13% | Phenylephrine, ephedrine, norepinephrine | MAP >65 mmHg, CVP >5 mmHg | Phenylephrine, ephedrine, norepinephrine |  |  |
| Weinberg 2019^61^ | Hepatic resection | Arterial line (FloTrac/EV1000) | Fluids |  | Discretion of the anaesthetist | Low | CDC |
|  |  | SVV <15/20%, MAP within 20% of baseline, CI >2.2 L/min/m^2^ | Venoconstrictor, inotropes, inodilator, vasoconstrictor, antihypertensive, beta-blocker, diuretic | CVP <8 mmHg | Discretion of the anaesthetist |  |  |
| Weinberg 2017^62^ | Pancreaticoduode-  nectomy | Arterial line (FloTrac/EV1000) | Crystalloids | - | Discretion anaesthetist | SC | CDC |
|  |  | SVV <20%, MAP within 20% of baseline, CI >2.0L/min/ m^2^ | Inotropes, vasoconstrictors, inodilators, arterial-dilator, beta-blocker, antihypertensive | Discretion anaesthetist | Vasoactive medication  Discretion anaesthetist |  |  |
| WenKui 2010^63^ | Elective gastrointestinal neoplasm surgery | Serum lactate | Colloids | - | Fluids | SC | Surgical evacuation of pus |
|  |  | Serum lactate <1.6 mmol/L | - | HR, BP, UO, CVP, changed body weight | - |  |  |
| Wijeysundera 2022^64^ | Elective non-cardiac surgery (postoperative stay >48h | Arterial line (Flotrac /EV1000) | Crystalloids, colloids |  | Discretion anaesthetist | Low | NI |
|  |  | PPV <9%, SVV <5%, SV increase <10%, MAP >60 mmHg, SBP >90 mmHg | Ephedrine, phenylephrine, norepinephrine | MAP >60 mmHg, SBP >90 mmHg | Discretion anaesthetist |  |  |
| Wilson 1999^65^ | Major surgery (urology, general and vascular surgery) | Arterial line | - | - | Discretion anaesthetist | SC | NI |
|  |  | DO_2_I ≥600 ml/min | Adrenaline or dopexamine | - | Discretion anaesthetist |  |  |
| Wu 2024 ^66^ | Laparoscopic radical resection of colorectal cancer | Arterial line | Crystalloids, colloids, RBC’s | Arterial line | Crystalloids, colloids, RBC’s | Low | π |
|  |  | PPV ≤13%, MAP 20% of baseline, HR 55-100 bpm, UO >0.5 ml/kgz | Norepinephrine, phenylephrine,  ephedrine | MAP 20% of baseline, HR  55-100 bpm, UO >0.5 ml/kg/h, Hb >70 g/L | Norepinephrine, phenylephrine,  ephedrine |  |  |
| Wu 2021^67^ | Elective open or laparoscopic partial nephrectomy | Arterial line (LiDCO) | Crystalloids | - | Crystalloids | Low | § |
|  |  | SVV <6%, CI 3.0-4.0 l/min/  m2, MAP >95 mmHg | Dobutamine, norepinephrine | BP <20% baseline, UO >0.5 ml/kg/h | Ephedrine |  |  |
| Wu 2017^68^ | Neurosurgery (supratentorial neoplasm surgery) | Arterial line (FloTrac/Vigileo), CVC | Colloids | CVC | Colloids | SC | Clinical diagnosis |
|  |  | SVV ≤12%, MAP >70 mmHg, CI ≥2.5 l/min/ m^2^ | Ephedrine, phenylephrine, dobutamine | CVP ≥8 mmHg, MAP ≥70 mmHg or >80% of baseline | Ephedrine, phenylephrine |  |  |
| Xia 2024^69^ | Radical resection rectal cancer | Arterial line | Crystalloids, colloids, RBC’s | Arterial line | Crystalloids, colloids, RBC’s | High | NI |
|  |  | PPV <13%; SBP >80% baseline, MAP >60 mmHg | Norepinephrine | SBP >80% baseline, Hb <100 g/L, MAP >60 mmHg | Norepinephrine, ephedrine |  |  |

| Yildiz 2022^70^ | Open major gynaecologic oncologic surgery (duration >2h) | Finger sensor | Crystalloids | - | Crystalloids | Low | NI |  |
| --- | --- | --- | --- | --- | --- | --- | --- | --- |
|  |  | PVI <13%, MAP >65 mmHg | Norepinephrine | MAP >65 mmHg | Norepinephrine |  |  |  |
| Yoon 2023^71^ | Open radical cystectomy | Arterial line (FloTrac/EV1000), CVL | Crystalloids | Arterial line (FloTrac/EV1000), CVL | Discretion anaesthetist | Low |  |  |
|  |  | SVI increase <10%, MAP reduction >20% from baseline or >60 mmHg, CI >2.5 l/min/m^2^, Hb >8 g/dl | Dobutamine, norepinephrine, ephedrine, phenylephrine | Discretion anaesthetist | Discretion anaesthetist |  |  |  |
| Zakhaleva 2013^72^ | Elective open or laparoscopic bowel resection with primary anastomosis | Oesophageal Doppler (CardioQ) | Colloids | - | Crystalloids | High | NI |  |
|  |  | SV increase <10%, FTc >350ms | - | Based on loss | - |  |  |  |
| Zhang 2018^73^ | Spinal stenosis surgery | Arterial line (FloTrac/Vigileo) | Colloids | - | Crystalloids, colloids | SC | NI |  |
|  |  | SVV <14%, SV increase <10% | - | According to ‘4-2-1 formula’ | - |  |  |  |
| Zhang 2012^74^ | Elective open gastrointestinal surgery (blood loss <500 ml) | Arterial line (Datex Ohmeda S/5 Monitor) | Crystalloids, colloids | - | Crystalloids | SC | NI |  |
|  |  | PPV ≤11% | - | UO >0.5 ml/kg/h, CVP ≥4 mmHg | - |  |  |  |
| Zhao 2018^75^ | Radical operation of gastrointestinal cancers (stomach, colon, rectum) | Arterial line (FloTrac/Vigileo) | Colloids, *Wan Wen (Unclear)*, RBCs | - | Crystalloids, colloids, RBC | SC | NI |  |
|  |  | SVV <13%, SV <10% increase, Hb ≥ 80 g/l | Vasoactive agents | MAP ≥65 mmHg or +/-30% of baseline, HR 60-90 bpm | - |  |  |  |
| **Abbreviations**  BIS = bispectral index score; Bpm = beats per minute; CD = Clavien-Dindo; CDC: Centers for Disease Control and Prevention; CI = cardiac index; CPB = cardiopulmonary bypass; CVC = central venous catheter; CVL = central venous line; CVP = central venous pressure; CRC = colorectal cancer; dPP = delta pulse pressure; DO_2_ = oxygen delivery; DO_2_I = oxygen delivery index; esSVI = estimated stroke volume index; esCCO = estimated continuous cardiac output; esCCI = estimated continuous cardiac index; FTc = flow time corrected; Hb = haemoglobin; HR = heart rate; Ht = haematocrit; GI = gastrointestinal; ICU = intensive care unit; INVOS = near-infrared spectroscopy sensors; IV = intravenous; ITBVI = intrathoracic blood volume index; MAP = mean arterial pressure; NI = no information; O_2_ER= oxygen extraction ratio; PCWP = pulmonary-capillary wedge pressure; PP = pulse pressure; PPV = pulse pressure variation; PVI = pulse pressure index; RBC = red blood cell; ROB = risk of bias; rScO_2_ = regional cerebral oxygen saturation; rSO_2_ = regional cerebral oxygen saturation; SAP = systolic arterial pressure; SaO_2_ = arterial oxygen saturation; SBP = systolic blood pressure; SC = some concerns; ScVO_2_ = central venous oxygen saturation; SV = stroke volume; SVI = stroke volume index; SVV = stroke volume variation; VO2 = UO = urine output  **Definition SSI**  §: Fever > 38 °C, increased white blood cell count (> 12 × 109/L) and elevated inflammatory biomarkers necessitating upgrading antibiotic treatment  ¶ Major complication: Infection (proven Infection, i.e. respiratory, urinary, abdominal, wound with signs of SEPSIS). Minor complication: Uncomplicated infections (not requiring intravenous antibiotic therapy and with no signs of sepsis, i.e. uncomplicated urinary tract infections).  ¤ Wound infection: phlegmon + antibiotics or drainage. Intra-abdominal infection: computed tomography + antibiotics  Ω Wound complication: wound dehiscence requiring surgical exploration or drainage of pus from the operation wound with or without isolation of organisms.  † Postoperative complications were diagnosed by a combination of high clinical suspicion, radiograph or ultrasound evidence (pneumonia, hypervolemia, anastomotic leakage), or laboratory testing (wound infection, pneumonia, urinary tract infection), according to generally applicable guidelines and as reported by a physician in the patient’s medical file.  π Incision infection: The incision exhibited erythema, edema, increased temperature, and tenderness with purulent discharge, and the pathogen of pus was positive. Anastomotic leak: The presence of abdominal pain, abdominal distention, purulent secretions drainage, and confirmed imaging findings was observed. | | | | | | | | |
| 1. Aaen AA, Voldby AW, Storm N, et al. Goal-directed fluid therapy in emergency abdominal surgery: a randomised multicentre trial. *Br J Anaesth* 2021; **127**(4): 521-31.  2. Arslan-Carlon VT, Kay See: Dalbagni, Guido: Pedoto, Alessia C.: Herr, Harry W.: Bochner, Bernard H.: Cha, Eugene K.: Donahue, Timothy F.: Fischer, Mary: Donat, S. Machele. Goal-directed versus Standard Fluid Therapy to Decrease Ileus after Open Radical Cystectomy: A Prospective Randomized Controlled Trial. *Anesthesiology* 2020; **133**(2): 293-303.  3. Bahlmann HH, I.: Nilsson, L. Goal-directed therapy during transthoracic oesophageal resection does not improve outcome: Randomised controlled trial. *European Journal of Anaesthesiology* 2019; **36**(2): 153-61.  4. Bartha EA, C.: Imnell, A.: Fernlund, M. E.: Andersson, L. E.: Kalman, S. Randomized controlled trial of goal-directed haemodynamic treatment in patients with proximal femoral fracture. *British Journal of Anaesthesia* 2013; **110**(4): 545-53.  5. Benes JC, I.: Altmann, P.: Hluchy, M.: Kasal, E.: Svitak, R.: Pradl, R.: Stepan, M. Intraoperative fluid optimization using stroke volume variation in high risk surgical patients: Results of prospective randomized study. *Critical Care* 2010; **14**(3): R118.  6. Bisgaard JG, T.: RØnholm, E.: Toft, P. Haemodynamic optimisation in lower limb arterial surgery: Room for improvement? *Acta Anaesthesiologica Scandinavica* 2013; **57**(2): 189-98.  7. Bisgaard JG, T.: RØnholm, E.: Toft, P. Optimising stroke volume and oxygen delivery in abdominal aortic surgery: A randomised controlled trial. *Acta Anaesthesiologica Scandinavica* 2013; **57**(2): 178-88.  8. Brandstrup BS, P. E.: Rasmussen, M.: Belhage, B.: Rodt, S. Å: Hansen, B.: Moller, D. R.: Lundbech, L. B.: Andersen, N.: Berg, V.: Thomassen, N.: Andersen, S. T.: Simonsen, L. Which goal for fluid therapy during colorectal surgery is followed by the best outcome: Near-maximal stroke volume or zero fluid balance? *British Journal of Anaesthesia* 2012; **109**(2): 191-9.  9. Calvo-Vecino JMR-M, J.: Mythen, M. G.: Casans-Francés, R.: Balik, A.: Artacho, J. P.: Martínez-Hurtado, E.: Serrano Romero, A.: Fernández Pérez, C.: Asuero de Lis, S.: Errazquin, A. T.: Gil Lapetra, C.: Motos, A. A.: Reche, E. G.: Medraño Viñas, C.: Villaba, R.: Cobeta, P.: Ureta, E.: Montiel, M.: Mané, N.: Martínez Castro, N.: Horno, G. A.: Salas, R. A.: Bona García, C.: Ferrer Ferrer, M. L.: Franco Abad, M.: García Lecina, A. C.: Antón, J. G.: Gascón, G. H.: Peligro Deza, J.: Pascual, L. P.: Ruiz Garcés, T.: Roberto Alcácer, A. T.: Badura, M.: Terrer Galera, E.: Fernández Casares, A.: Martínez Fernández, M. C.: Espinosa, Á: Abad-Gurumeta, A.: Feldheiser, A.: López Timoneda, F.: Zuleta-Alarcón, A.: Bergese, S. Effect of goal-directed haemodynamic therapy on postoperative complications in low–moderate risk surgical patients: a multicentre randomised controlled trial (FEDORA trial). *British Journal of Anaesthesia* 2018; **120**(4): 734-44.  10. Cecconi MF, N.: Langiano, N.: Divella, M.: Costa, M. G.: Rhodes, A.: Rocca, G. D. Goal-directed haemodynamic therapy during elective total hip arthroplasty under regional anaesthesia. *Critical Care* 2011; **15**(3): R132.  11. Cheng XQZ, J. Y.: Wu, H.: Zuo, Y. M.: Tang, L. L.: Zhao, Q.: Gu, E. W. Outcomes of individualized goal-directed therapy based on cerebral oxygen balance in high-risk patients undergoing cardiac surgery: A randomized controlled trial. *Journal of Clinical Anesthesia* 2020; **67**: 110032.  12. Colantonio LC, C.: Fabrizi, L.: Marcelli, M. E.: Sofra, M.: Giannarelli, D.: Garofalo, A.: Forastiere, E. A randomized trial of goal directed vs. standard fluid therapy in cytoreductive surgery with hyperthermic intraperitoneal chemotherapy. *Journal of gastrointestinal surgery : official journal of the Society for Surgery of the Alimentary Tract* 2015; **19**(4): 722-9.  13. Davies SJ, Yates DR, Wilson RJT, et al. A randomised trial of non-invasive cardiac output monitoring to guide haemodynamic optimisation in high risk patients undergoing urgent surgical repair of proximal femoral fractures (ClearNOF trial NCT02382185). *Perioper Med (Lond)* 2019; **8**: 8.  14. Castro GIP, Castro R, Lima RME, Santos BND, Navarro ELLH. Fluid therapy and pulmonary complications in abdominal surgeries: randomized controlled trial. *Braz J Anesthesiol* 2024; **74**(4): 844500.  15. de Waal EEC, Frank M, Scheeren TWL, et al. Perioperative goal-directed therapy in high-risk abdominal surgery. A multicenter randomized controlled superiority trial. *J Clin Anesth* 2021; **75**: 110506.  16. Diaper JS, E.: Barcelos, G. K.: Luise, S.: Schorer, R.: Ellenberger, C.: Licker, M. Goal-directed hemodynamic therapy versus restrictive normovolemic therapy in major open abdominal surgery: A randomized controlled trial. *Surgery (United States)* 2021; **169**(5): 1164-74.  17. Elgendy MA, Esmat IM, Kassim DY. Outcome of intraoperative goal-directed therapy using Vigileo/FloTrac in high-risk patients scheduled for major abdominal surgeries: a prospective randomized trial. *Egyptian Journal of Anaesthesia* 2017; **33**(3): 263-9.  18. Forget PL, F.: De Kock, M. Goal-directed fluid management based on the pulse oximeter-derived pleth variability index reduces lactate levels and improves fluid management. *Anesthesia and Analgesia* 2010; **111**(4): 910-4.  19. Funcke S, Schmidt G, Bergholz A, et al. Cardiac index-guided therapy to maintain optimised postinduction cardiac index in high-risk patients having major open abdominal surgery: the multicentre randomised iPEGASUS trial. *Br J Anaesth* 2024; **133**(2): 277-87.  20. Gan TJ, Soppitt A, Maroof M, et al. Goal-directed intraoperative fluid administration reduces length of hospital stay after major surgery. *Anesthesiology* 2002; **97**(4): 820-6.  21. Göçmen D, Köksal C, Abitağaoğlu S, Yıldırım Ar A. Comparison of the effects of intraoperative goal directed and conventional fluid management on the inferior vena cava collapsibility index and postoperative complications in geriatric patients operated from proximal femoral nail surgery. *Türk Geriatri Dergisi* 2023; **26**(1): 37-47.  22. Godai KM, A.: Kanmura, Y. The effects of hemodynamic management using the trend of the perfusion index and pulse pressure variation on tissue perfusion: a randomized pilot study. *Ja Clin Rep* 2019; **5**(1): 72.  23. Gomez-Izquierdo JC, Trainito A, Mirzakandov D, et al. Goal-directed Fluid Therapy Does Not Reduce Primary Postoperative Ileus after Elective Laparoscopic Colorectal Surgery: A Randomized Controlled Trial. *Anesthesiology* 2017; **127**(1): 36-49.  24. Jammer IU, A.: Erichsen, C.: Lødemel, O.: Østgaard, G. Does central venous oxygen saturation-directed fluid therapy affect postoperative morbidity after colorectal surgery?: A randomized assessor-blinded controlled trial. *Anesthesiology* 2010; **113**(5): 1072-80.  25. Kaufmann KBB, W.: Rexer, J.: Loeffler, T.: Heinrich, S.: Konstantinidis, L.: Buerkle, H.: Goebel, U. Evaluation of hemodynamic goal-directed therapy to reduce the incidence of bone cement implantation syndrome in patients undergoing cemented hip arthroplasty - a randomized parallel-arm trial. *BMC Anesthesiology* 2018; **18**(1): 63.  26. Kim HJK, E. J.: Lee, H. J.: Min, J. Y.: Kim, T. W.: Choi, E. C.: Kim, W. S.: Koo, B. N. Effect of goal-directed haemodynamic therapy in free flap reconstruction for head and neck cancer. *Acta Anaesthesiologica Scandinavica* 2018; **62**(7): 903-14.  27. Kumar L, Rajan S, Baalachandran R. Outcomes associated with stroke volume variation versus central venous pressure guided fluid replacements during major abdominal surgery. *J Anaesthesiol Clin Pharmacol* 2016; **32**(2): 182-6.  28. Kumar L, Kanneganti YS, Rajan S. Outcomes of implementation of enhanced goal directed therapy in high-risk patients undergoing abdominal surgery. *Indian J Anaesth* 2015; **59**(4): 228-33.  29. Le Teurnier Y, Rozec B, Degryse C, et al. Optimization of cerebral oxygenation based on regional cerebral oxygen saturation monitoring during carotid endarterectomy: a Phase III multicenter, double-blind randomized controlled trial. *Anaesth Crit Care Pain Med* 2024; **43**(4): 101388.  30. Liu XZ, P.: Liu, M. X.: Ma, J. L.: Wei, X. C.: Fan, D. Preoperative carbohydrate loading and intraoperative goal-directed fluid therapy for elderly patients undergoing open gastrointestinal surgery: a prospective randomized controlled trial. *BMC Anesthesiology* 2021; **21**(1): 157.  31. Liu FL, Jing: Zhang, Weixia: Liu, Zhongkai: Dong, Ling: Wang, Yuelan. Randomized controlled trial of regional tissue oxygenation following goal-directed fluid therapy during laparoscopic colorectal surgery. *International journal of clinical and experimental pathology* 2019; **12**(12): 4390-9.  32. Lopes MRO, M. A.: Pereira, V. O. S.: Lemos, I. P. B.: Auler Jr, J. O. C.: Michard, F. Goal-directed fluid management based on pulse pressure variation monitoring during high-risk surgery: A pilot randomized controlled trial. *Critical Care* 2007; **11**(5): R100.  33. Luo JX, J.: Liu, J.: Liu, B.: Liu, L.: Chen, G. Goal-directed fluid restriction during brain surgery: a prospective randomized controlled trial. *Annals of Intensive Care* 2017; **7**(1): 16.  34. Mahrose R, Kasem AA. Pulse Pressure Variation-Based Intraoperative Fluid Management Versus Traditional Fluid Management for Colon Cancer Patients Undergoing Open Mass Resection and Anastomosis: A Randomized Controlled Trial. *Anesth Pain Med* 2023; **13**(4): e135659.  35. Mayer J, Boldt J, Mengistu AM, Rohm KD, Suttner S. Goal-directed intraoperative therapy based on autocalibrated arterial pressure waveform analysis reduces hospital stay in high-risk surgical patients: a randomized, controlled trial. *Crit Care* 2010; **14**(1): R18.  36. McKenny MC, P.: Wong, A.: Farren, M.: Gleeson, N.: Walsh, C.: O'Malley, C.: Dowd, N. A randomised prospective trial of intra-operative oesophageal Doppler-guided fluid administration in major gynaecological surgery. *Anaesthesia* 2013; **68**(12): 1224-31.  37. Mishra N, Rath GP, Bithal PK, Chaturvedi A, Chandra PS, Borkar SA. Effect of Goal-Directed Intraoperative Fluid Therapy on Duration of Hospital Stay and Postoperative Complications in Patients Undergoing Excision of Large Supratentorial Tumors. *Neurol India* 2022; **70**(1): 108-14.  38. Mostafa Elebieby MG, Abdelkhalek M, Eldadamony ZEM, Mohammed MN. Goal-directed fluid therapy compared to liberal fluid therapy in patients subjected to colorectal surgery. *Egyptian Journal of Anaesthesia* 2023; **39**(1): 687-95.  39. Nicklas JYD, O.: Leistenschneider, M.: Sellhorn, C.: Schön, G.: Winkler, M.: Daum, G.: Schwedhelm, E.: Schröder, J.: Fisch, M.: Schmalfeldt, B.: Izbicki, J. R.: Bauer, M.: Coldewey, S. M.: Reuter, D. A.: Saugel, B. Personalised haemodynamic management targeting baseline cardiac index in high-risk patients undergoing major abdominal surgery: a randomised single-centre clinical trial. *British Journal of Anaesthesia* 2020; **125**(2): 122-32.  40. Osawa EA, Rhodes A, Landoni G, et al. Effect of Perioperative Goal-Directed Hemodynamic Resuscitation Therapy on Outcomes Following Cardiac Surgery: A Randomized Clinical Trial and Systematic Review. *Crit Care Med* 2016; **44**(4): 724-33.  41. Pearse RM, Harrison DA, MacDonald N, et al. Effect of a perioperative, cardiac output-guided hemodynamic therapy algorithm on outcomes following major gastrointestinal surgery: a randomized clinical trial and systematic review. *JAMA* 2014; **311**(21): 2181-90.  42. Peng KL, J.: Cheng, H.: Ji, F. H. Goal-directed fluid therapy based on stroke volume variations improves fluid management and gastrointestinal perfusion in patients undergoing major orthopedic surgery. *Medical Principles and Practice* 2014; **23**(5): 413-20.  43. Pestaña DE, E.: Eden, A.: Nájera, D.: Collar, L.: Aldecoa, C.: Higuera, E.: Escribano, S.: Bystritski, D.: Pascual, J.: Fernández-Garijo, P.: De Prada, B.: Muriel, A.: Pizov, R. Perioperative goal-directed hemodynamic optimization using noninvasive cardiac output monitoring in major abdominal surgery: A prospective, randomized, multicenter, pragmatic trial: POEMAS study (PeriOperative goal-directed thErapy in Major Abdominal Surgery). *Anesthesia and Analgesia* 2014; **119**(3): 579-87.  44. Phan TDDS, B.: Rattray, M. J.: Johnston, M. J.: Cowie, B. S. A randomised controlled trial of fluid restriction compared to oesophageal Doppler-guided goal-directed fluid therapy in elective major colorectal surgery within an Enhanced Recovery After Surgery program. *Anaesth Intens Care* 2014; **42**(6): 752-60.  45. Pillai PM, I.: Gaughan, M.: Snowden, C.: Nesbitt, I.: Durkan, G.: Johnson, M.: Cosgrove, J.: Thorpe, A. A double-blind randomized controlled clinical trial to assess the effect of doppler optimized intraoperative fluid management on outcome following radical cystectomy. *Journal of Urology* 2011; **186**(6): 2201-6.  46. Ranucci M, Johnson I, Willcox T, et al. Goal-directed perfusion to reduce acute kidney injury: A randomized trial. *J Thorac Cardiovasc Surg* 2018; **156**(5): 1918-27 e2.  47. Reisinger KW, Willigers HM, Jansen J, et al. Doppler-guided goal-directed fluid therapy does not affect intestinal cell damage but increases global gastrointestinal perfusion in colorectal surgery: a randomized controlled trial. *Colorectal Dis* 2017; **19**(12): 1081-91.  48. Sandham JD, Hull RD, Brant RF, et al. A randomized, controlled trial of the use of pulmonary-artery catheters in high-risk surgical patients. *N Engl J Med* 2003; **348**(1): 5-14.  49. Scheeren TWLW, C.: Gerlach, H.: Marx, G. Goal-directed intraoperative fluid therapy guided by stroke volume and its variation in high-risk surgical patients: A prospective randomized multicentre study. *Journal of Clinical Monitoring and Computing* 2013; **27**(3): 225-33.  50. Shoemaker WC, Appel PL, Kram HB, Waxman K, Lee TS. Prospective trial of supranormal values of survivors as therapeutic goals in high-risk surgical patients. *Chest* 1988; **94**(6): 1176-86.  51. Smetkin AA, Kirov MY, Kuzkov VV, et al. Single transpulmonary thermodilution and continuous monitoring of central venous oxygen saturation during off-pump coronary surgery. *Acta Anaesthesiol Scand* 2009; **53**(4): 505-14.  52. Srinivasa S, Taylor MH, Singh PP, Yu TC, Soop M, Hill AG. Randomized clinical trial of goal-directed fluid therapy within an enhanced recovery protocol for elective colectomy. *Br J Surg* 2013; **100**(1): 66-74.  53. Stens J, Hering JP, van der Hoeven CWP, et al. The added value of cardiac index and pulse pressure variation monitoring to mean arterial pressure-guided volume therapy in moderate-risk abdominal surgery (COGUIDE): a pragmatic multicentre randomised controlled trial. *Anaesthesia* 2017; **72**(9): 1078-87.  54. Szturz PF, P.: Kula, R.: Neiser, J.: Ševčík, P.: Benes, J. Multi-parametric functional hemodynamic optimization improves postsurgical outcome after intermediate risk open gastrointestinal surgery: A randomized controlled trial. *Minerva Anestesiologica* 2019; **85**(3): 244-54.  55. Tang W, Qiu Y, Lu H, Xu M, Wu J. Stroke Volume Variation-Guided Goal-Directed Fluid Therapy Did Not Significantly Reduce the Incidence of Early Postoperative Complications in Elderly Patients Undergoing Minimally Invasive Esophagectomy: A Randomized Controlled Trial. *Front Surg* 2021; **8**: 794272.  56. Venn RS, A.: Richardson, P.: Poloniecki, J.: Grounds, M.: Newman, P. Randomized controlled trial to investigate influence of the fluid challenge on duration of hospital stay and perioperative morbidity in patients with hip fractures. *British Journal of Anaesthesia* 2002; **88**(1): 65-71.  57. Vu P, Tran V, Tran T, et al. Effectiveness of goal-directed fluid therapy guided by estimated continuous cardiac output (esCCO) in major gastrointestinal surgeries: a randomized controlled trial. 2023: 371-8.  58. Wakeling HGM, M. R.: Jenkins, C. S.: Woods, W. G. A.: Miles, W. F. A.: Barclay, G. R.: Fleming, S. C. Intraoperative oesophageal Doppler guided fluid management shortens postoperative hospital stay after major bowel surgery. *British Journal of Anaesthesia* 2005; **95**(5): 634-42.  59. Wang DD, Li Y, Hu XW, Zhang MC, Xu XM, Tang J. Comparison of restrictive fluid therapy with goal-directed fluid therapy for postoperative delirium in patients undergoing spine surgery: a randomized controlled trial. *Perioper Med (Lond)* 2021; **10**(1): 48.  60. Wang Y, Zhang Y, Zheng J, et al. Intraoperative pleth variability index-based fluid management therapy and gastrointestinal surgical outcomes in elderly patients: a randomised controlled trial. *Perioper Med (Lond)* 2023; **12**(1): 16.  61. Weinberg L, Ianno D, Churilov L, et al. Goal directed fluid therapy for major liver resection: A multicentre randomized controlled trial. *Ann Med Surg (Lond)* 2019; **45**: 45-53.  62. Weinberg L, Ianno D, Churilov L, et al. Restrictive intraoperative fluid optimisation algorithm improves outcomes in patients undergoing pancreaticoduodenectomy: A prospective multicentre randomized controlled trial. *PLoS One* 2017; **12**(9): e0183313.  63. WenKui YN, L.: JianFeng, G.: WeiQin, L.: ShaoQiu, T.: Zhihui, T.: Tao, G.: JuanJuan, Z.: FengChan, X.: Hui, S.: WeiMing, Z.: Jie-Shou, L. Restricted peri-operative fluid administration adjusted by serum lactate level improved outcome after major elective surgery for gastrointestinal malignancy. *Surgery* 2010; **147**(4): 542-52.  64. Wijeysundera D, Duncan D, Moreno Garijo J, et al. A randomised controlled feasibility trial of a clinical protocol to manage hypotension during major non‐cardiac surgery. *Anaesthesia* 2022.  65. Wilson JW, I.: Fawcett, J.: Whall, R.: Dibb, W.: Morris, C.: McManus, E. Reducing the risk of major elective surgery: Randomised controlled trial of preoperative optimisation of oxygen delivery. *British Medical Journal* 1999; **318**(7191): 1099-103.  66. Wu QR, Zhao ZZ, Fan KM, Cheng HT, Wang B. Pulse pressure variation guided goal-direct fluid therapy decreases postoperative complications in elderly patients undergoing laparoscopic radical resection of colorectal cancer: a randomized controlled trial. *Int J Colorectal Dis* 2024; **39**(1): 33.  67. Wu QFK, H.: Xu, Z. Z.: Li, H. J.: Mu, D. L.: Wang, D. X. Impact of goal-directed hemodynamic management on the incidence of acute kidney injury in patients undergoing partial nephrectomy: a pilot randomized controlled trial. *BMC Anesthesiology* 2021; **21**(1): 67.  68. Wu JM, Y.: Wang, T.: Xu, G.: Fan, L.: Zhang, Y. Goal-directed fluid management based on the auto-calibrated arterial pressure-derived stroke volume variation in patients undergoing supratentorial neoplasms surgery. *Int J Clin Exp Med* 2017; **10**(2): 3106-14.  69. Xia LC, Zhang K, Wang CW. Effects of fluid therapy combined with a preoperative glucose load regimen on postoperative recovery in patients with rectal cancer. *World J Gastrointest Surg* 2024; **16**(8): 2662-70.  70. Yildiz GO, Hergunsel GO, Sertcakacilar G, Akyol D, Karakas S, Cukurova Z. Perioperative goal-directed fluid management using noninvasive hemodynamic monitoring in gynecologic oncology. *Braz J Anesthesiol* 2022; **72**(3): 322-30.  71. Yoon HK, Hur M, Kim DH, Ku JH, Kim JT. The effect of goal-directed hemodynamic therapy on clinical outcomes in patients undergoing radical cystectomy: a randomized controlled trial. *BMC Anesthesiol* 2023; **23**(1): 339.  72. Zakhaleva J, Tam J, Denoya PI, Bishawi M, Bergamaschi R. The impact of intravenous fluid administration on complication rates in bowel surgery within an enhanced recovery protocol: a randomized controlled trial. *Colorectal Dis* 2013; **15**(7): 892-9.  73. Zhang N, Liang M, Zhang DD, et al. Effect of goal-directed fluid therapy on early cognitive function in elderly patients with spinal stenosis: A Case-Control Study. *Int J Surg* 2018; **54**(Pt A): 201-5.  74. Zhang JQ, H.: He, Z.: Wang, Y.: Che, X.: Liang, W. Intraoperative fluid management in open gastrointestinal surgery: Goal-directed versus restrictive. *Clinics* 2012; **67**(10): 1149-55.  75. Zhao GP, P.: Zhou, Y.: Li, J.: Jiang, H.: Shao, J. The accuracy and effectiveness of goal directed fluid therapy in plateau-elderly gastrointestinal cancer patients: a prospective randomized controlled trial. *Int J Clin Exp Med* 2018; **11**(8): 8516-22. | | | | | | | | |
|  | | | | | | | | |

# **Appendix 5.** **Forest plots of secondary outcomes**

## 5A. Mortality

Relative risk (RR) of mortality in studies with goal-directed haemodynamic therapy (GDHT) versus no specific fluid management (control). The figure shows the pooled RR. The squares and horizontal lines represent point estimates and corresponding 95% confidence intervals (CI) of the individual studies.


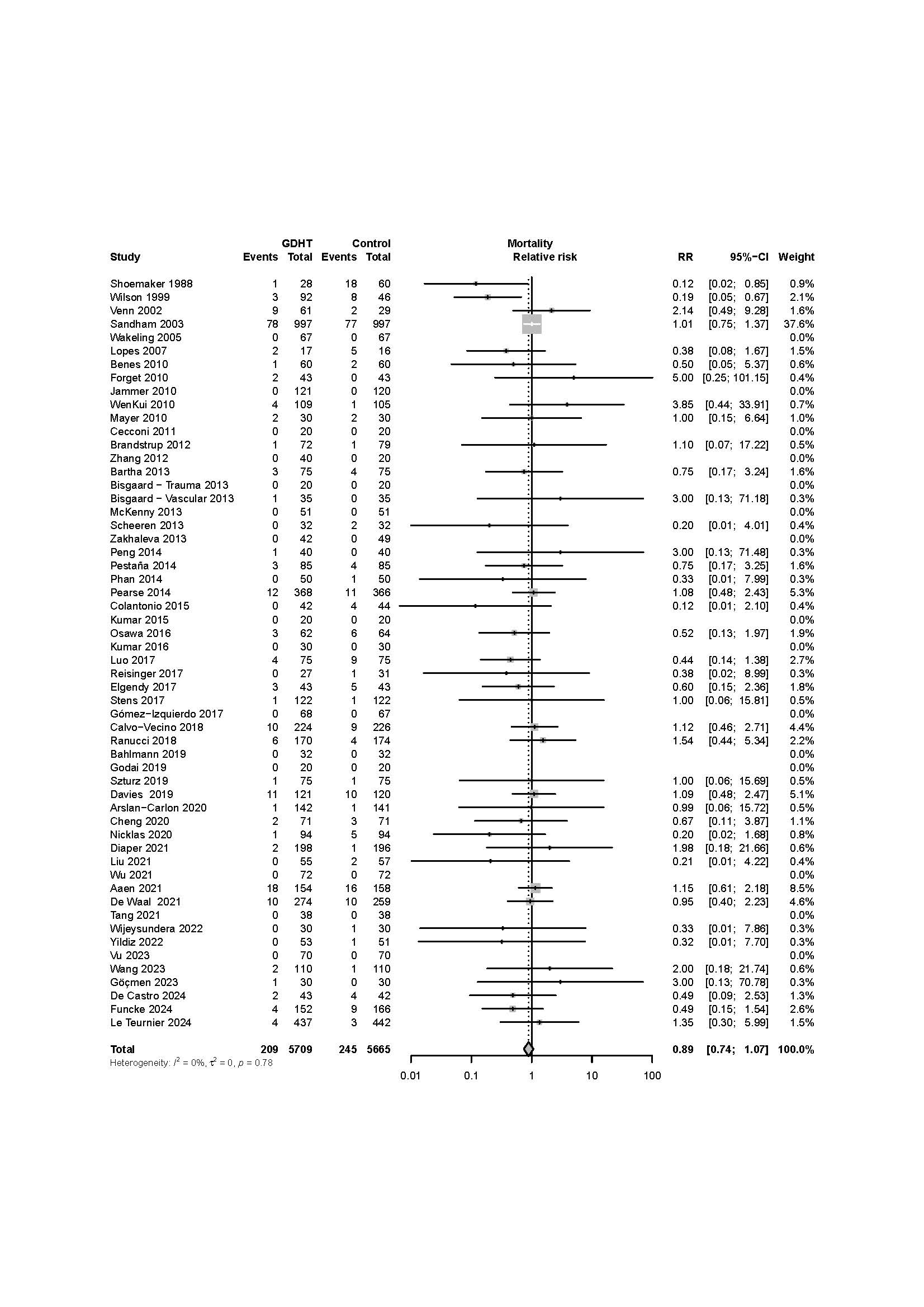


## 5B. Sepsis

Relative risk (RR) of the incidence of sepsis in studies with goal-directed haemodynamic therapy (GDHT) versus no specific fluid management (control). The figure shows the pooled RR. The squares and horizontal lines represent point estimates and corresponding 95% confidence intervals (CI) of the individual studies.


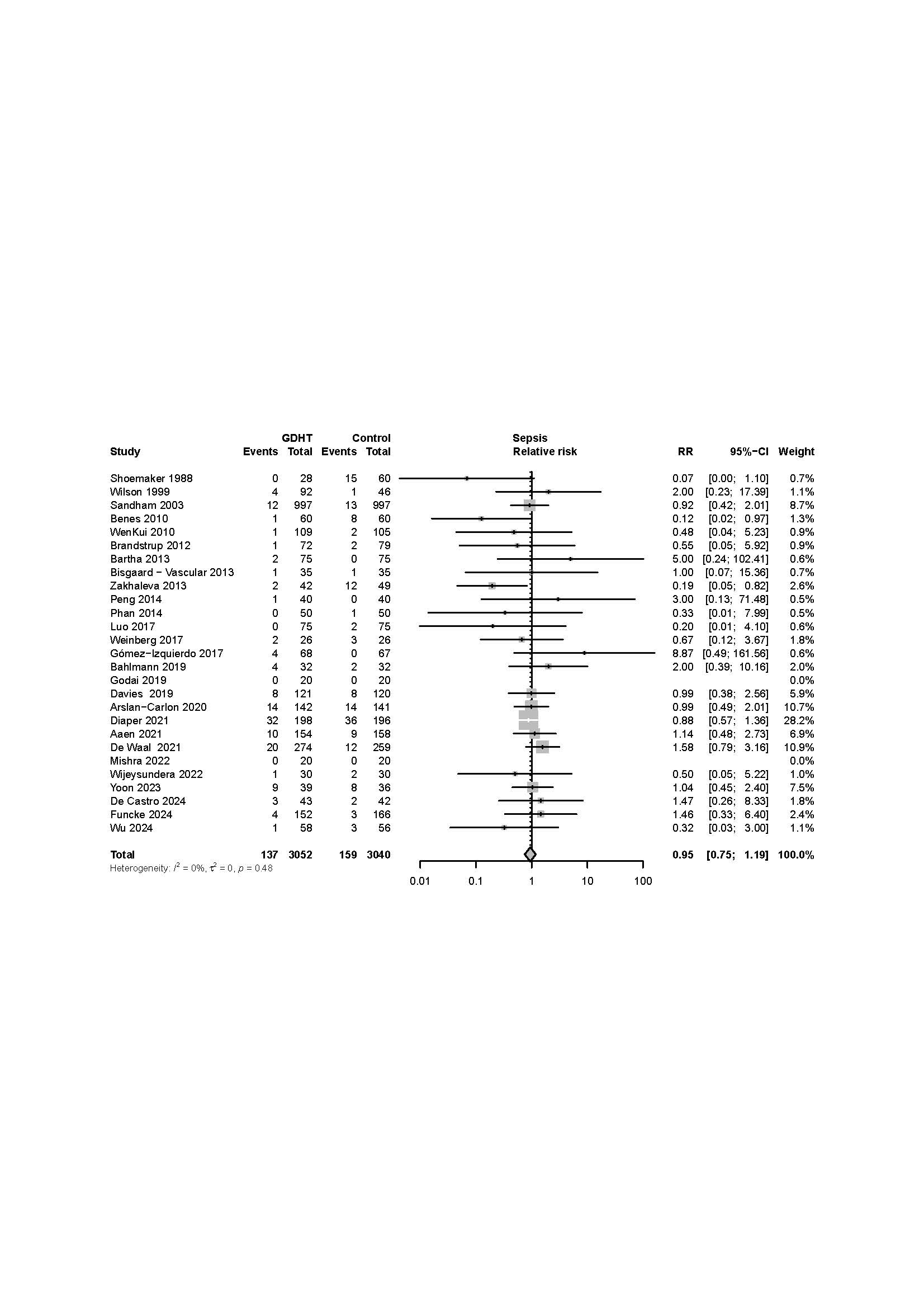


## 5C. Pneumonia

Relative risk (RR) of the incidence of pneumonia in studies with goal-directed haemodynamic therapy (GDHT) versus no specific fluid management (control). The figure shows the pooled RR. The squares and horizontal lines represent point estimates and corresponding 95% confidence intervals (CI) of the individual studies.


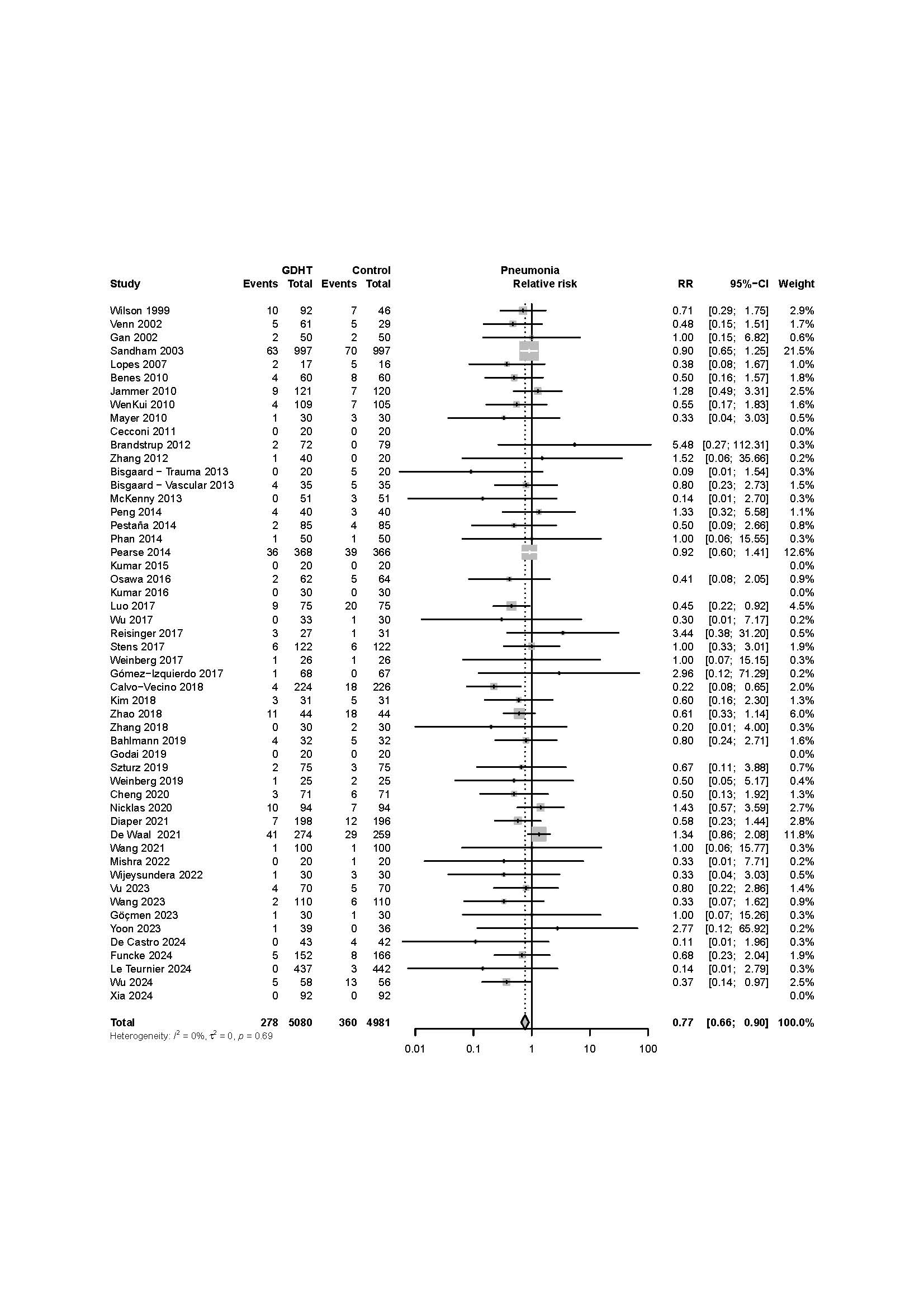


## 5D. Urinary tract infection

Relative risk (RR) of the incidence of urinary tract infection in studies with goal-directed haemodynamic therapy (GDHT) versus no specific fluid management (control). The figure shows the pooled RR. The squares and horizontal lines represent point estimates and corresponding 95% confidence intervals (CI) of the individual studies.


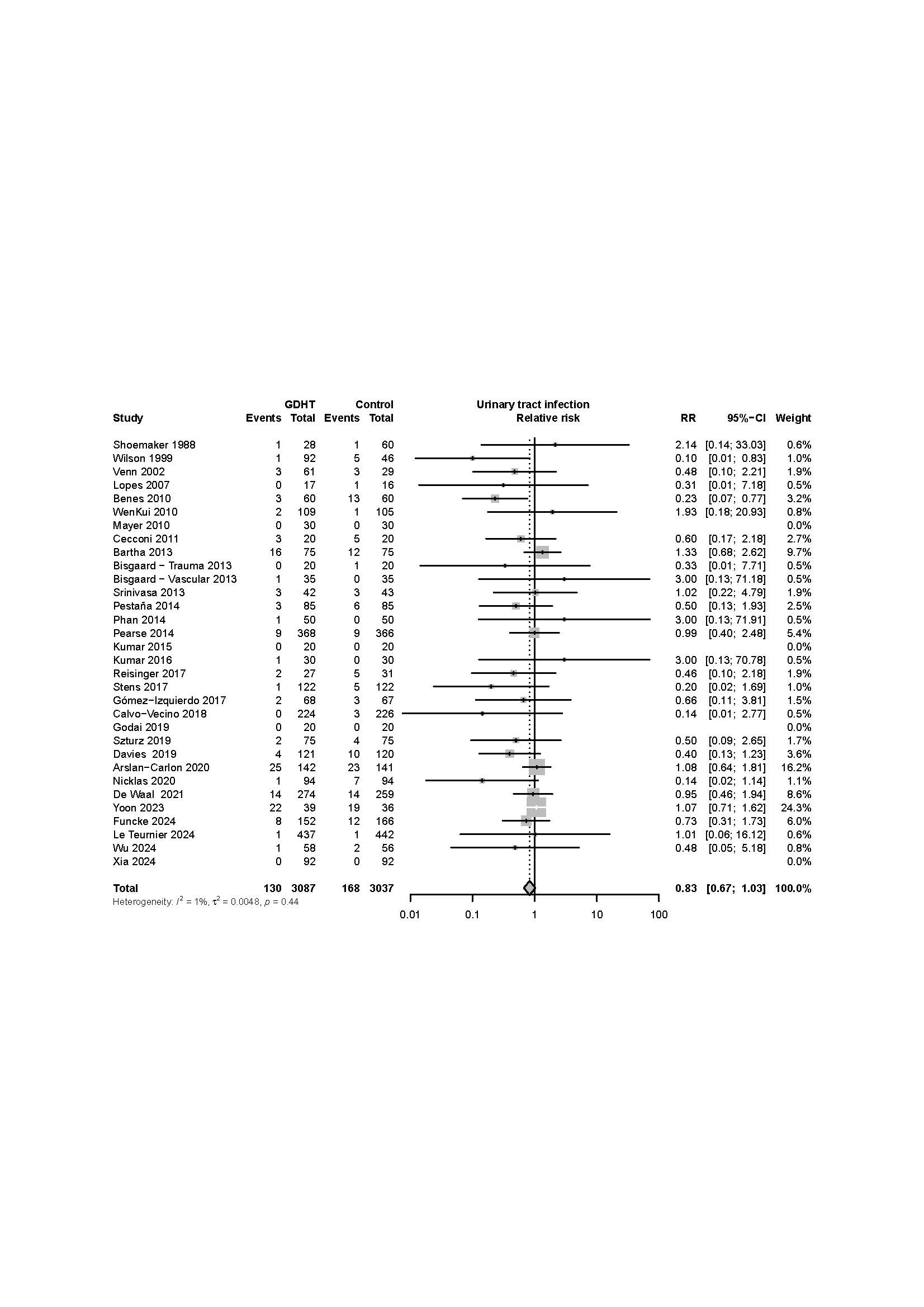


## 5E. Acute kidney injury

Relative risk of the incidence of acute kidney injury in studies with goal-directed haemodynamic therapy (GDHT) versus no specific fluid management (control). The figure shows the pooled relative risk (RR). The squares and horizontal lines represent point estimates and corresponding 95% confidence intervals (CI) of the individual studies.


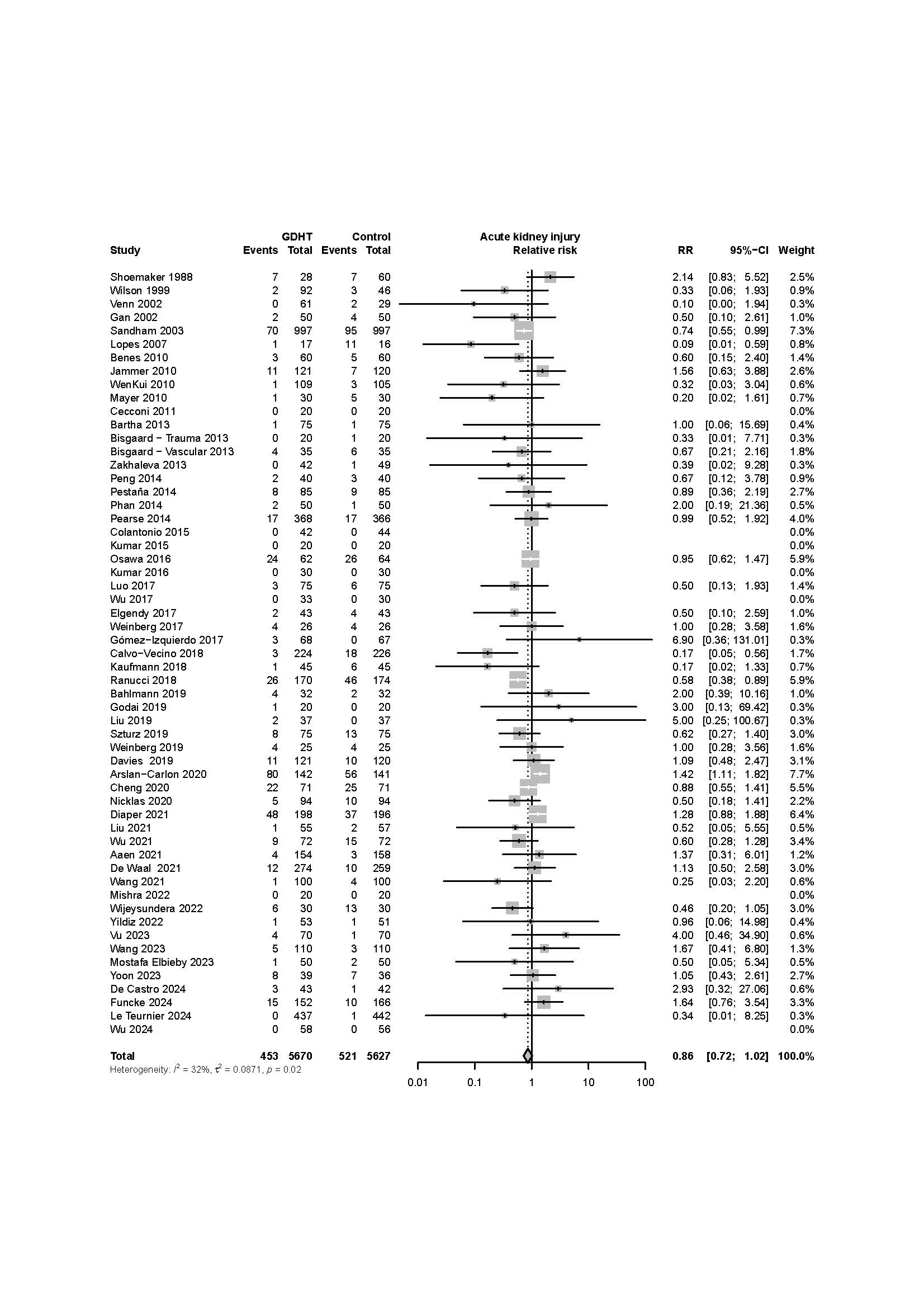


## 5F. Paralytic ileus

Relative risk of the incidence of paralytic ileus in studies with goal-directed haemodynamic therapy (GDHT) versus no specific fluid management (control). The figure shows the pooled relative risk (RR). The squares and horizontal lines represent point estimates and corresponding 95% confidence intervals (CI) of the individual studies.


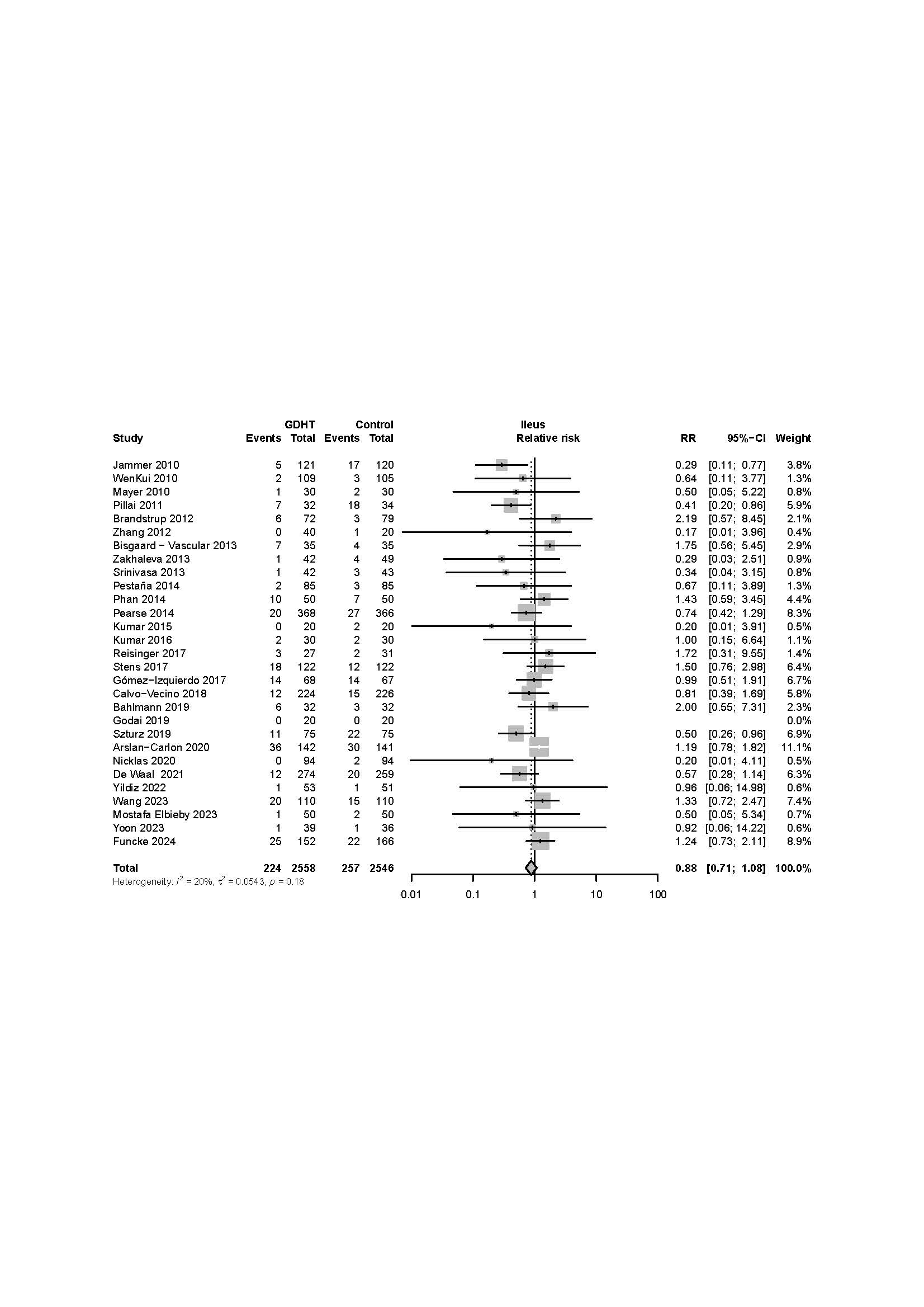


## 5G. Reoperation

Relative risk (RR) of the incidence of reoperation in studies with goal-directed haemodynamic therapy (GDHT) versus no specific fluid management (control). The figure shows the pooled RR. The squares and horizontal lines represent point estimates and corresponding 95% confidence intervals (CI) of the individual studies.


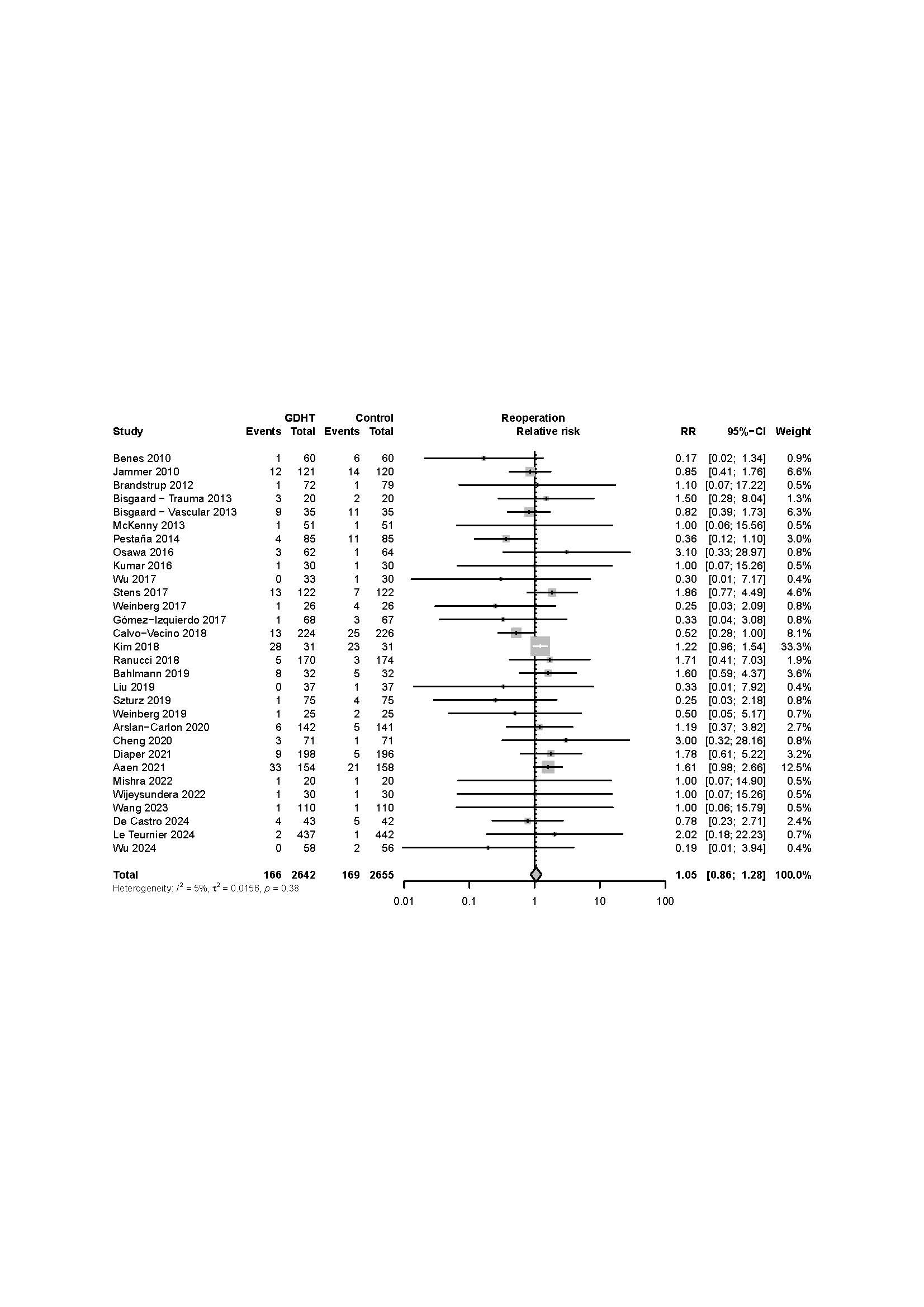


## 5H. Number of patients with ≥1 complication

Relative risk (RR) of the incidence of patients with one or more complications in studies with goal-directed haemodynamic therapy (GDHT) versus no specific fluid management (control). The figure shows the pooled RR. The squares and horizontal lines represent point estimates and corresponding 95% confidence intervals (CI) of the individual studies.


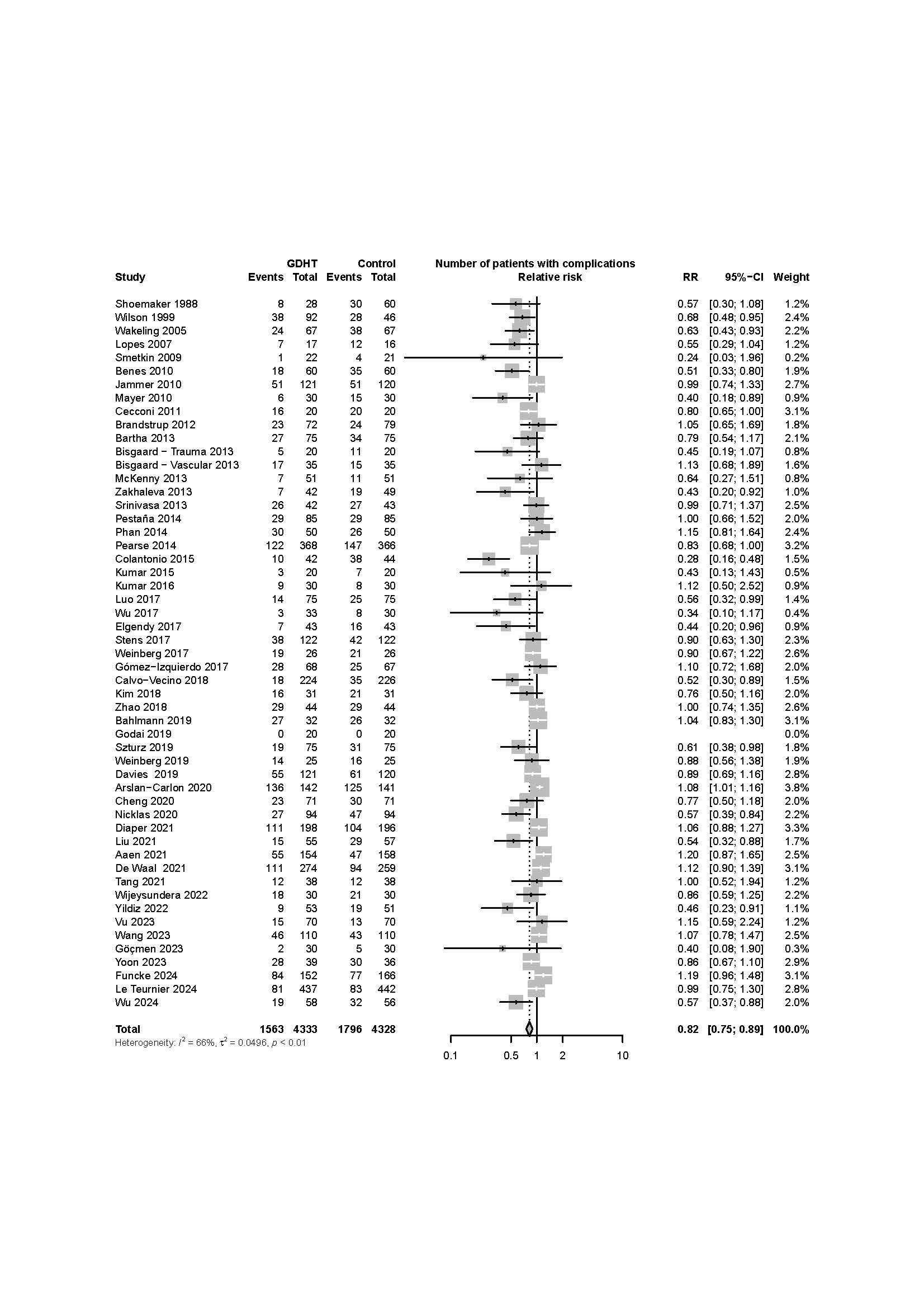


# **Appendix 6. Subgroup analysis for the risk of bias: low versus some concerns versus high risk of bias**

Relative risk (RR) of studies grouped for low, some concerns, or high risk of bias. The figure shows the pooled RR. The squares and horizontal lines represent point estimates and corresponding 95% confidence intervals (CI) of the individual studies.


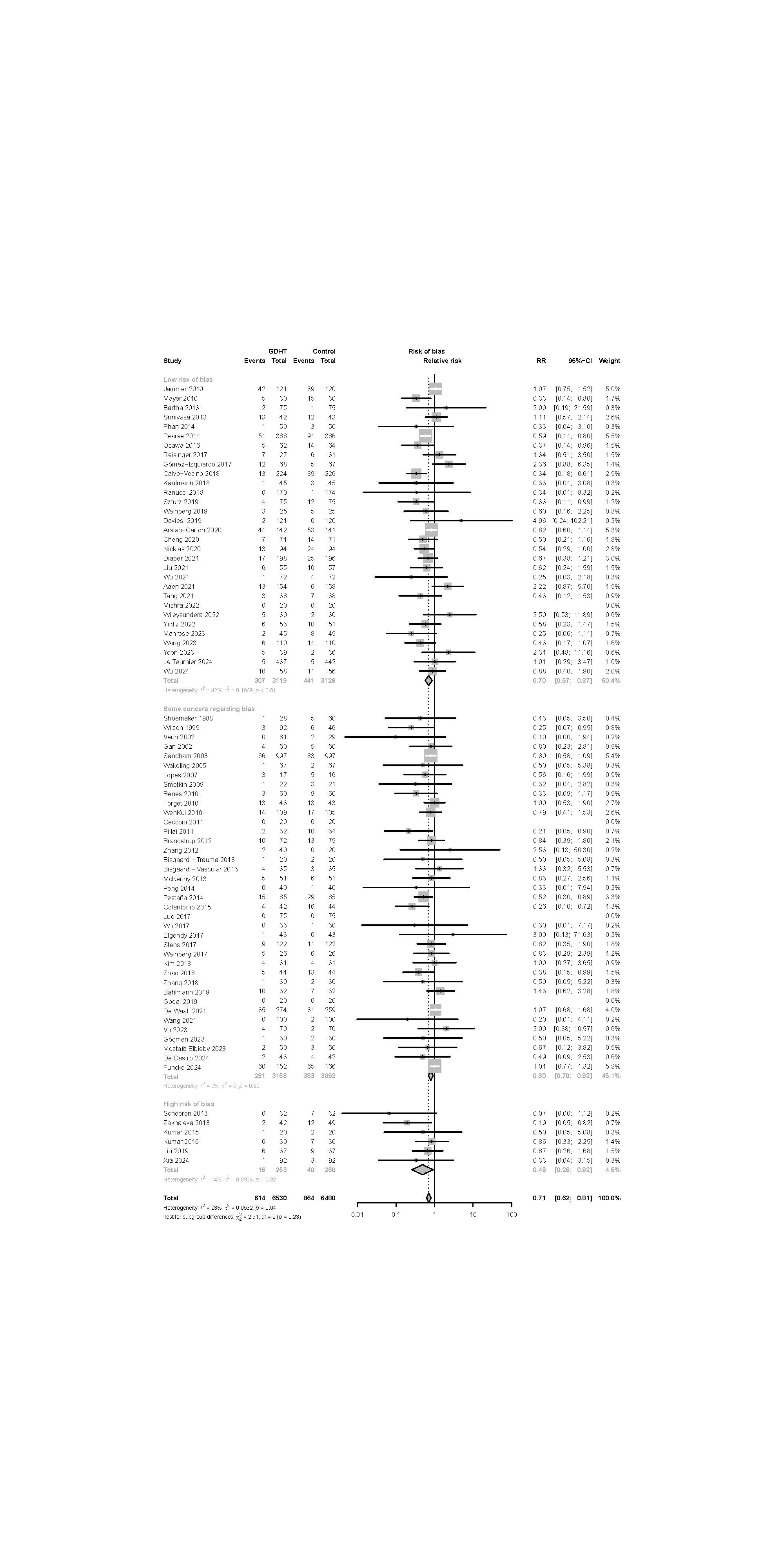


# **Appendix 7. Subgroup analyses for the adherence to the 5 T's of Saugel and colleagues**

## 7A. Target population

Relative risk (RR) of studies grouped for a high-risk patient population (at least 50% of the study population has an American Society of Anaesthesiologists (ASA) classification score higher than 2) or a low-risk patient population, and comparing studies with goal-directed haemodynamic therapy (GDHT) versus no specific fluid management (control). The figure shows the pooled RR. The squares and horizontal lines represent point estimates and corresponding 95% confidence intervals (CI) of the individual studies.


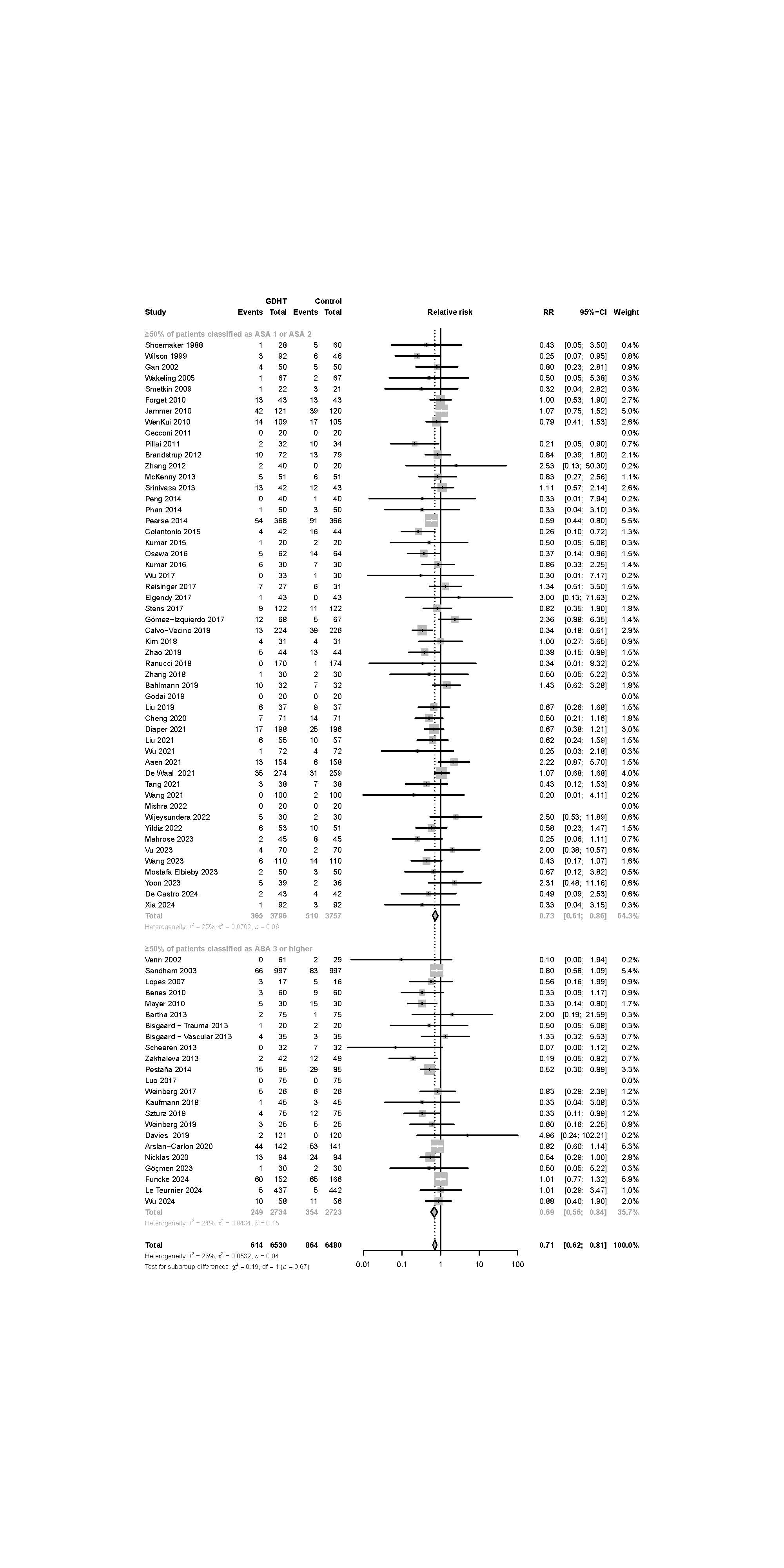


## 7B. Timing of intervention

Relative risk (RR) of studies grouped for those starting with goal-directed haemodynamic therapy (GDHT) before induction of anaesthesia versus studies starting with GDHT during or after induction of anaesthesia. The figure shows the pooled RR. The squares and horizontal lines represent point estimates and corresponding 95% confidence intervals (CI) of the individual studies.


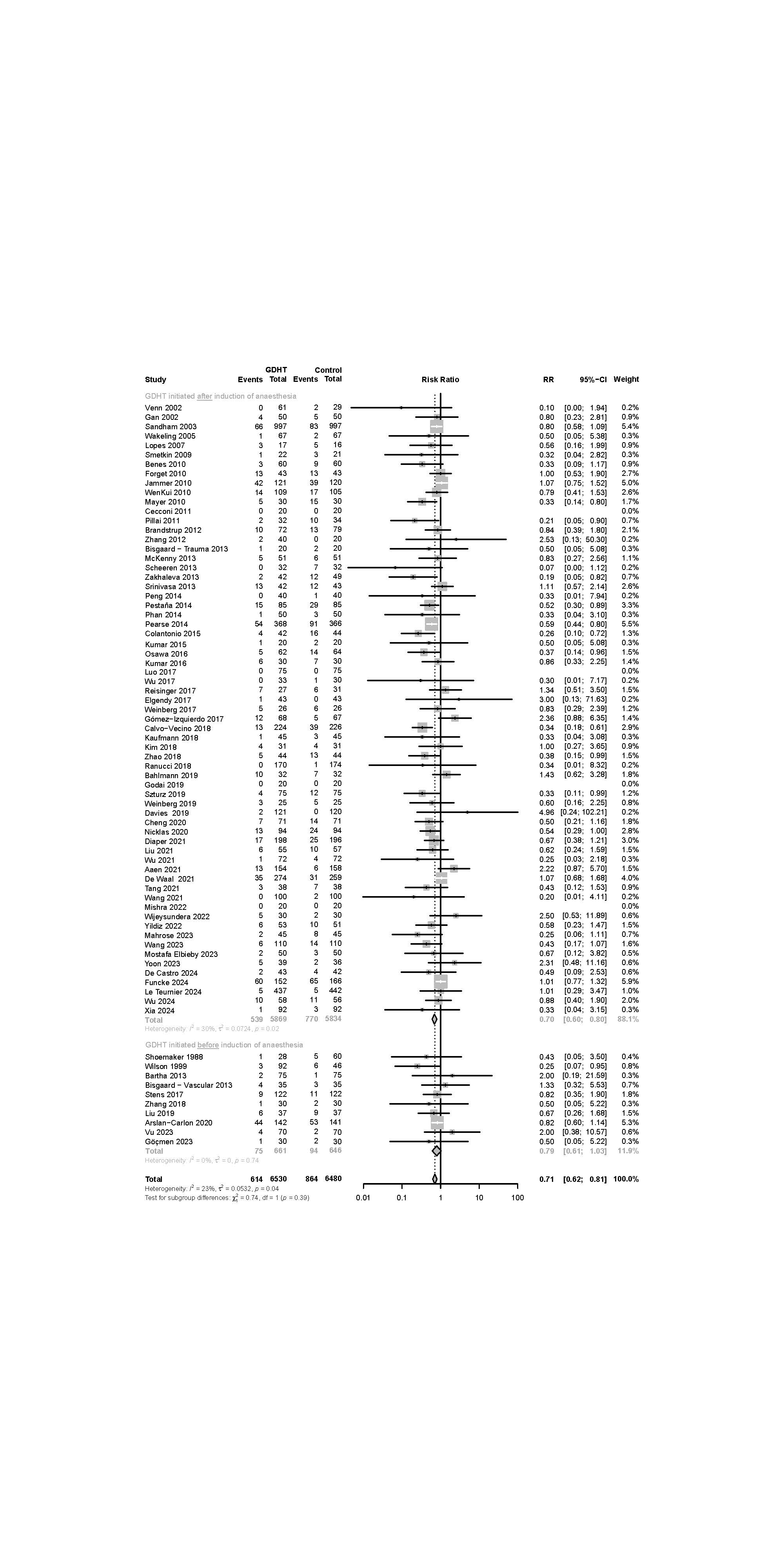


## 7C. Type of intervention: fluids and inotropes

Relative risk of studies grouped for those including fluids and inotropes in their goal-directed haemodynamic therapy (GDHT) algorithm versus studies with fluids and no use of in their GDHT algorithm. The figure shows the pooled RR. The squares and horizontal lines represent point estimates and corresponding 95% confidence intervals (CI) of the individual studies.


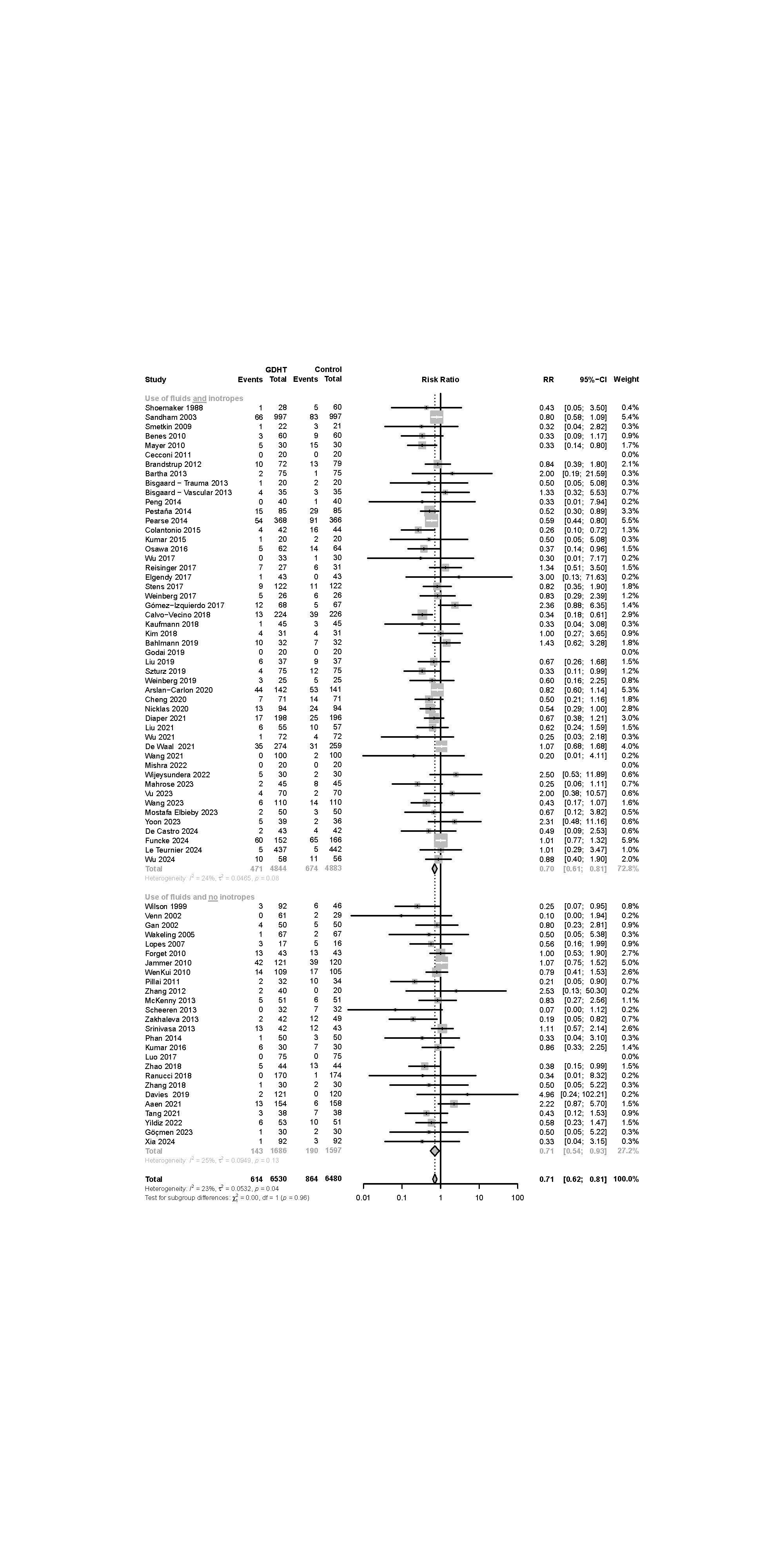


## 7D. Type of intervention: fluids and vasopressors

Relative risk (RR) of studies grouped for those including fluids and vasopressors in their goal-directed haemodynamic therapy (GDHT) algorithm versus studies with fluids and no use of in their GDHT algorithm. The figure shows the pooled RR. The squares and horizontal lines represent point estimates and corresponding 95% confidence intervals (CI) of the individual studies.


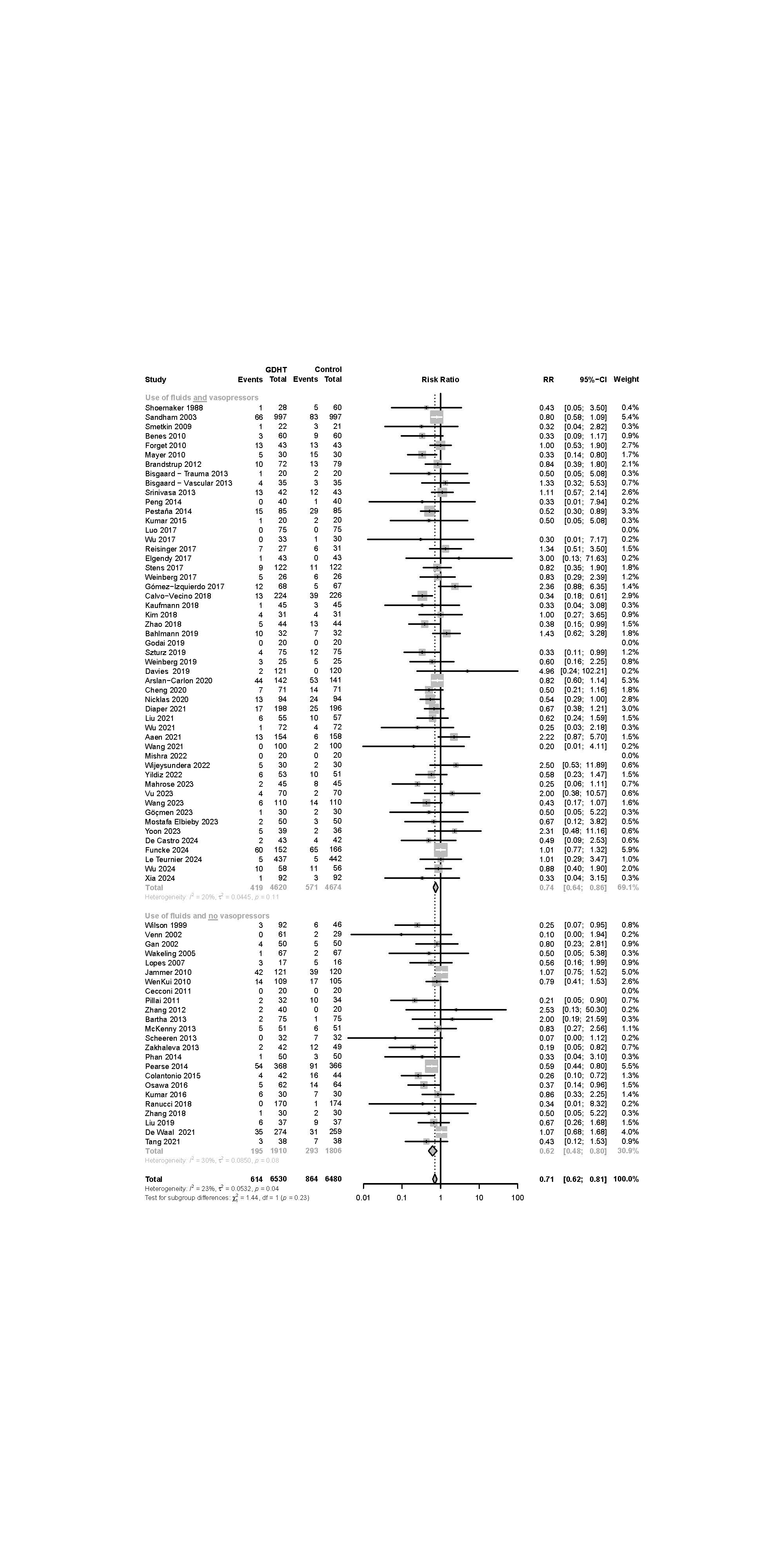


## 7E. Type of intervention: fluids, inotropes, and vasopressors

Relative risk (RR) of studies grouped for those including fluids, inotropes, and vasopressors in their goal-directed haemodynamic therapy (GDHT) algorithm versus studies not including all three types of interventions in their GDHT algorithm. The figure shows the pooled RR. The squares and horizontal lines represent point estimates and corresponding 95% confidence intervals (CI) of the individual studies.


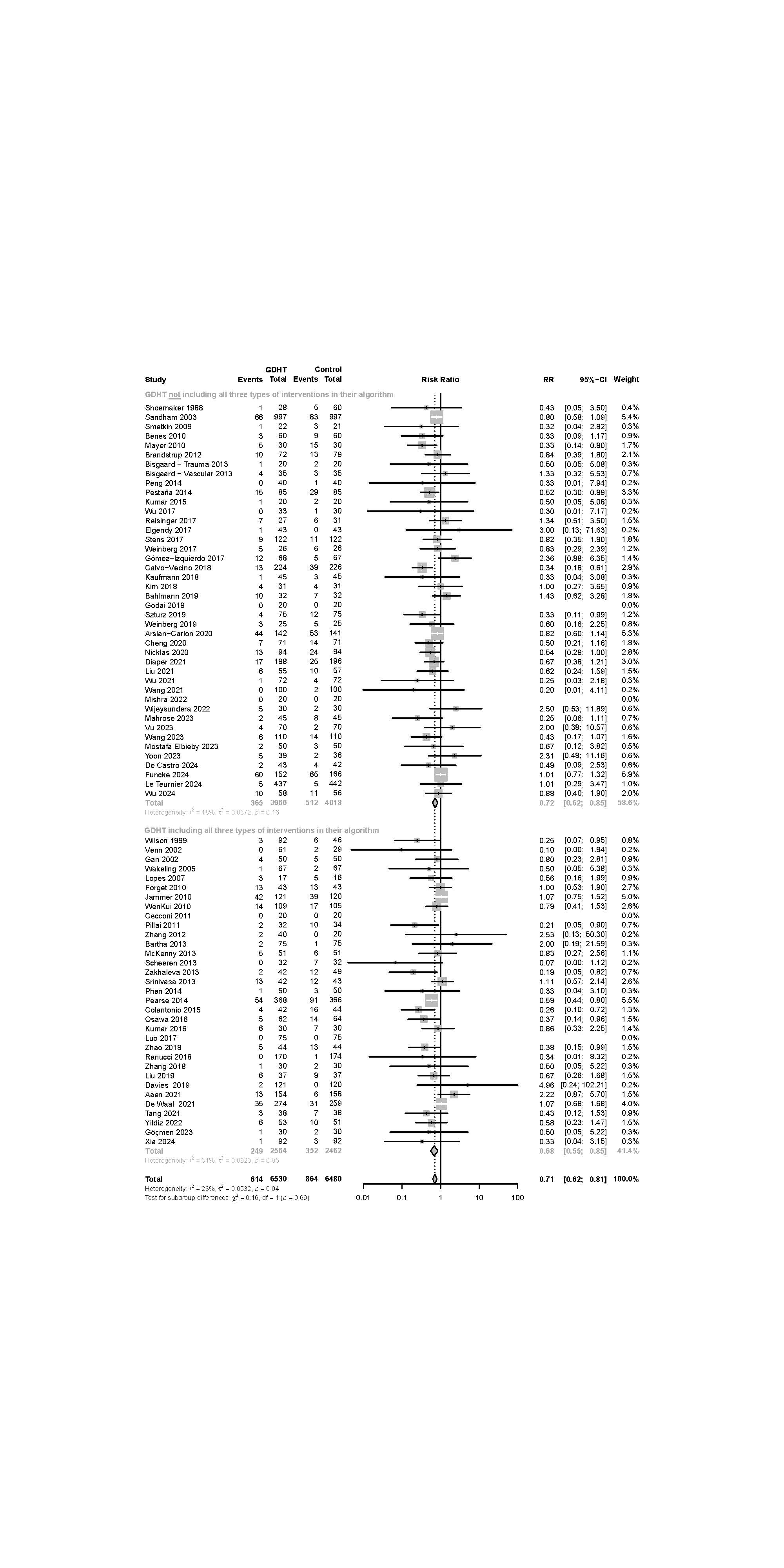


## 7F. Target variable

Relative risk of studies grouped for those including a target variable, reflecting blood flow (stroke volume, stroke volume index, cardiac output, cardiac index) in their goal-directed haemodynamic therapy (GDHT) algorithm versus studies not including a target variable in their GDHT algorithm. The figure shows the pooled RR. The squares and horizontal lines represent point estimates and corresponding 95% confidence intervals (CI) of the individual studies.


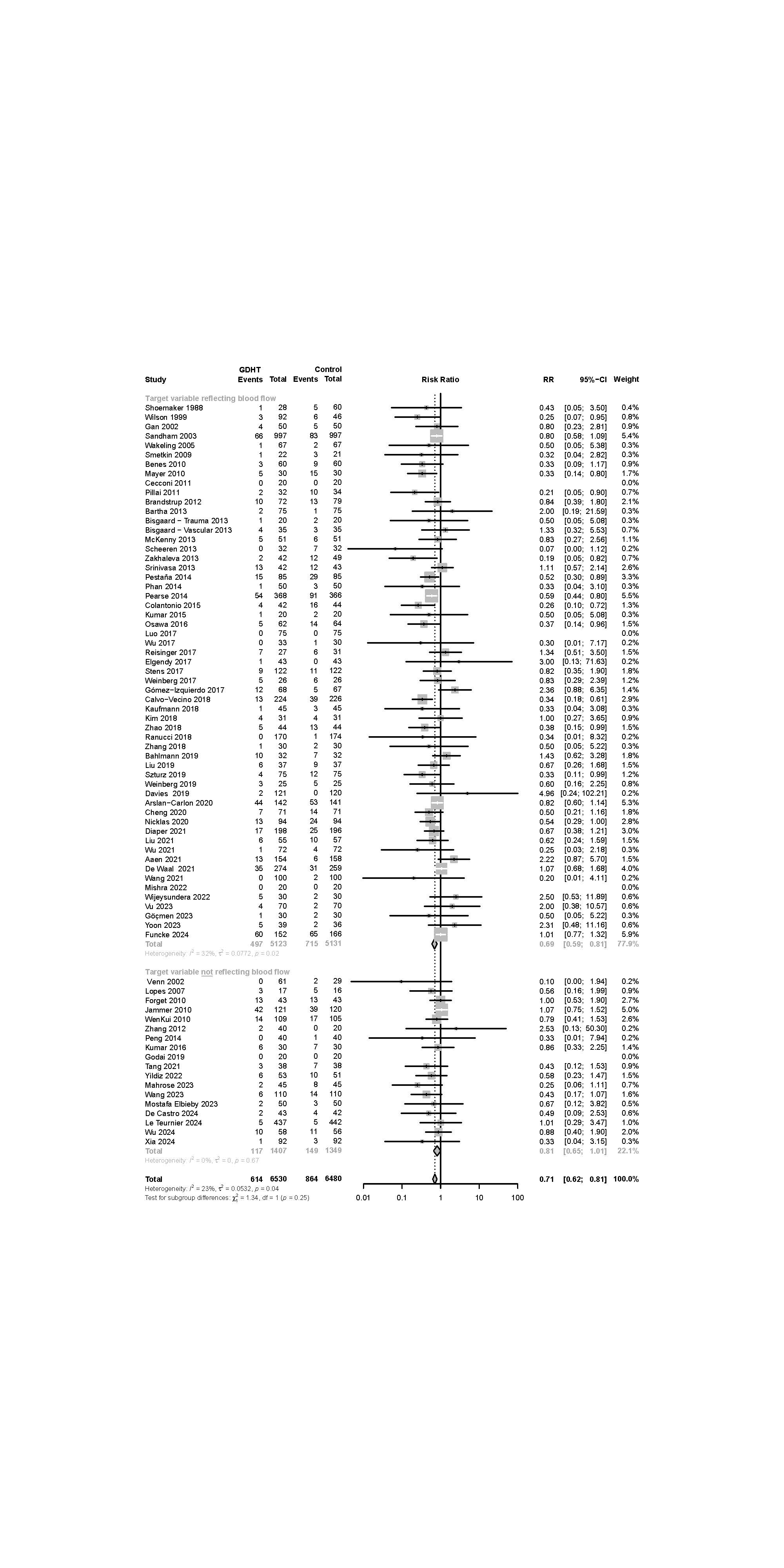


## 7G. Target value

Relative risk of studies grouped for those including a personalised target value in their goal-directed haemodynamic therapy (GDHT) algorithm versus studies not including a personalised target value in their GDHT algorithm. The figure shows the pooled RR. The squares and horizontal lines represent point estimates and corresponding 95% confidence intervals (CI) of the individual studies.


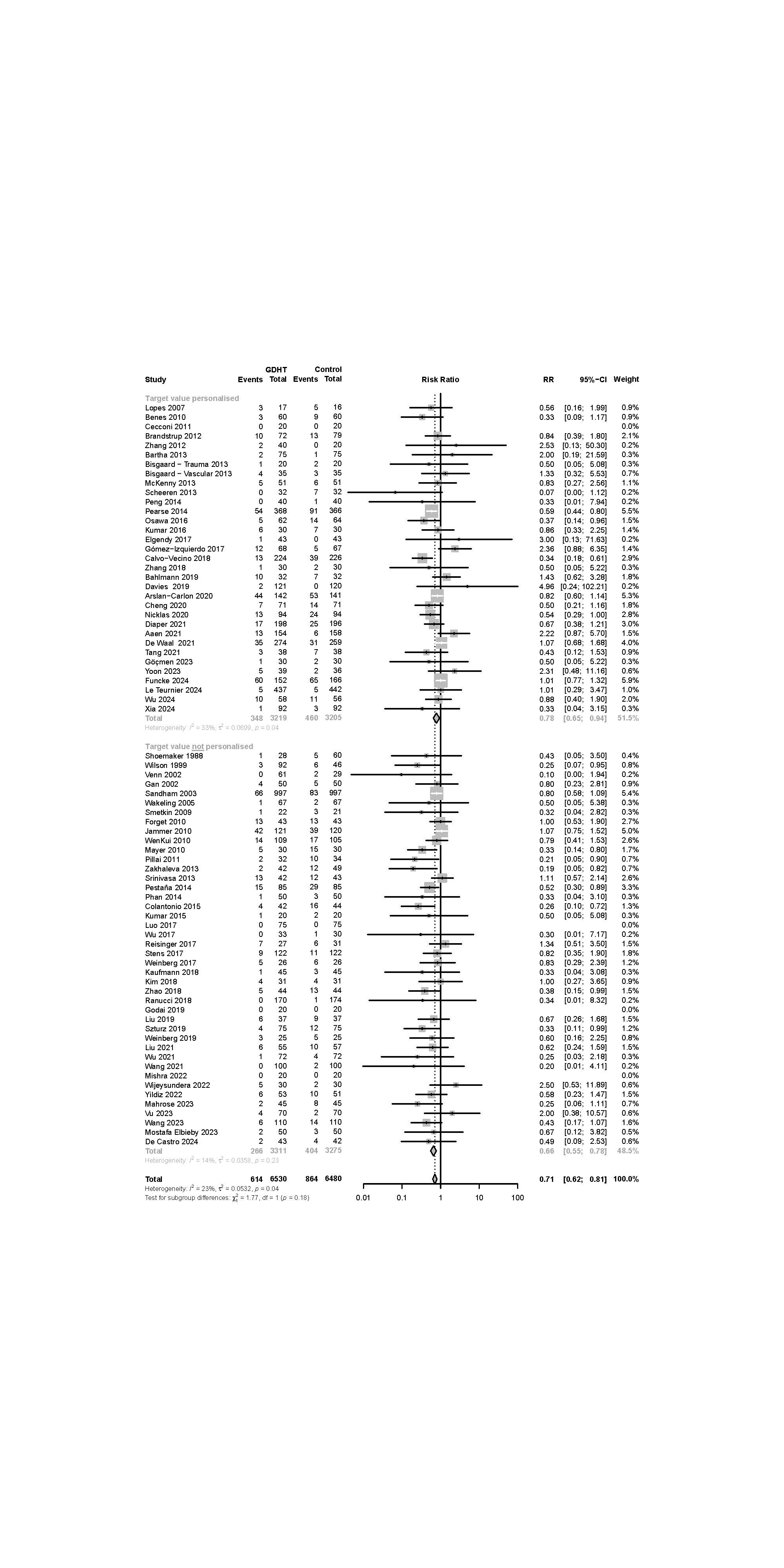


## **7H. Sum of 5T’s of Saugel and colleagues**

The 5T’s of Saugel and colleagues represent target population, timing of intervention, type of intervention (including all three types of intervention; fluids, inotropes and vasopressors), target variable, and personalised target variables. The relative risk (RR) of five distinct groups is associated with a different sum of the adherence to the 5T’s. The figure shows the pooled RR. The squares and horizontal lines represent point estimates and corresponding 95% confidence intervals (CI) of the individual studies.


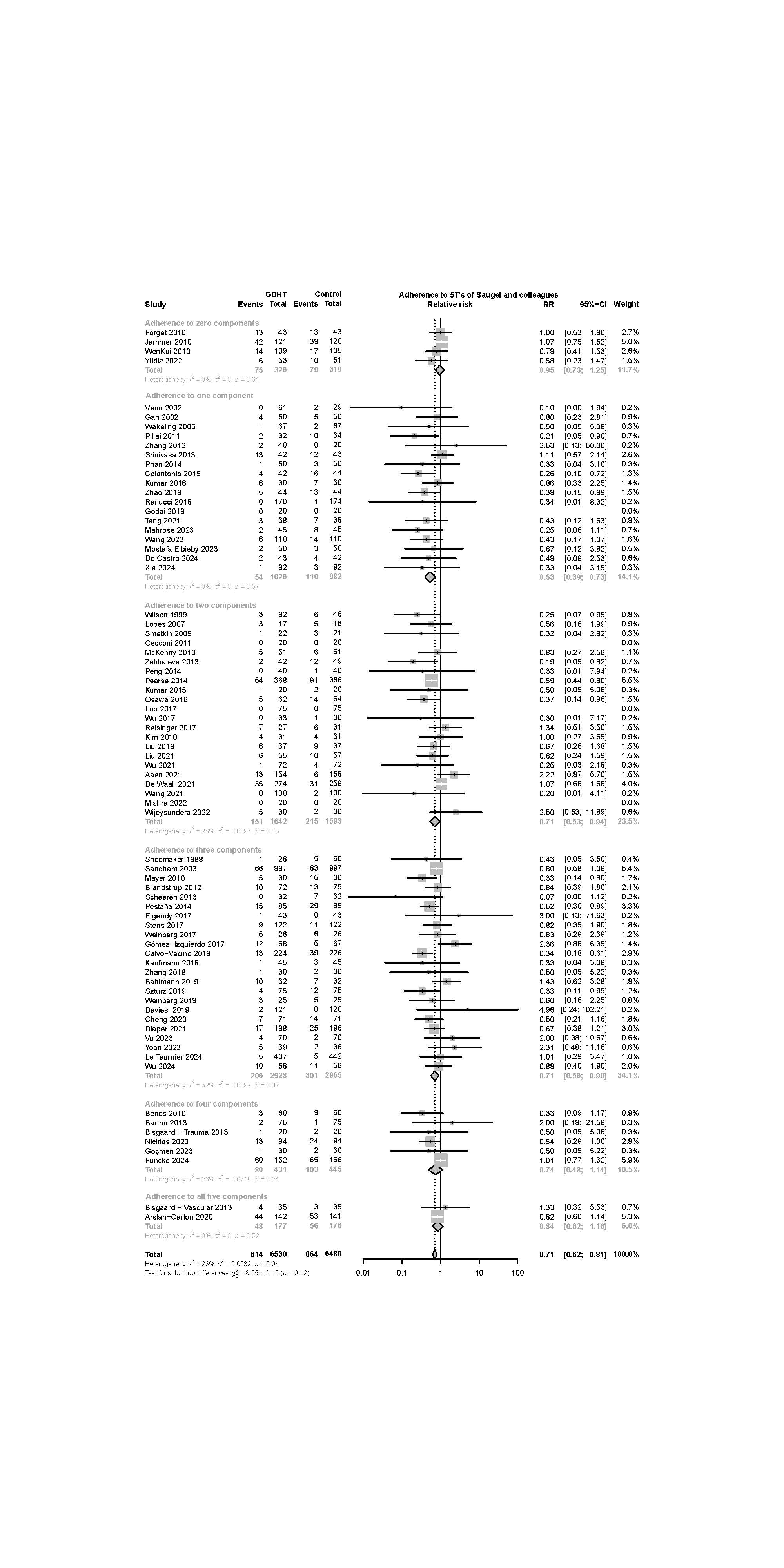


## 7I. Meta-regression bubble plot of the sum of all the 5Ts **of Saugel and colleagues**

The 5T’s of Saugel and colleagues represent target population, timing of intervention, type of intervention, target variable, and personalised target variables. Meta-regressions showed that adherence to the 5 T’s was not a significant effect size predictor with a regression coefficient of 0.02 (95% CI -0.08 to 0.12), which means that for every ‘additional T’, the effect size (relative risk) is expected to increase by 0.02. The solid dark line represents the regression coefficient. The solid light line represents the regression line for the relationship between the adherence to the 5T’s and the Relative Risk.


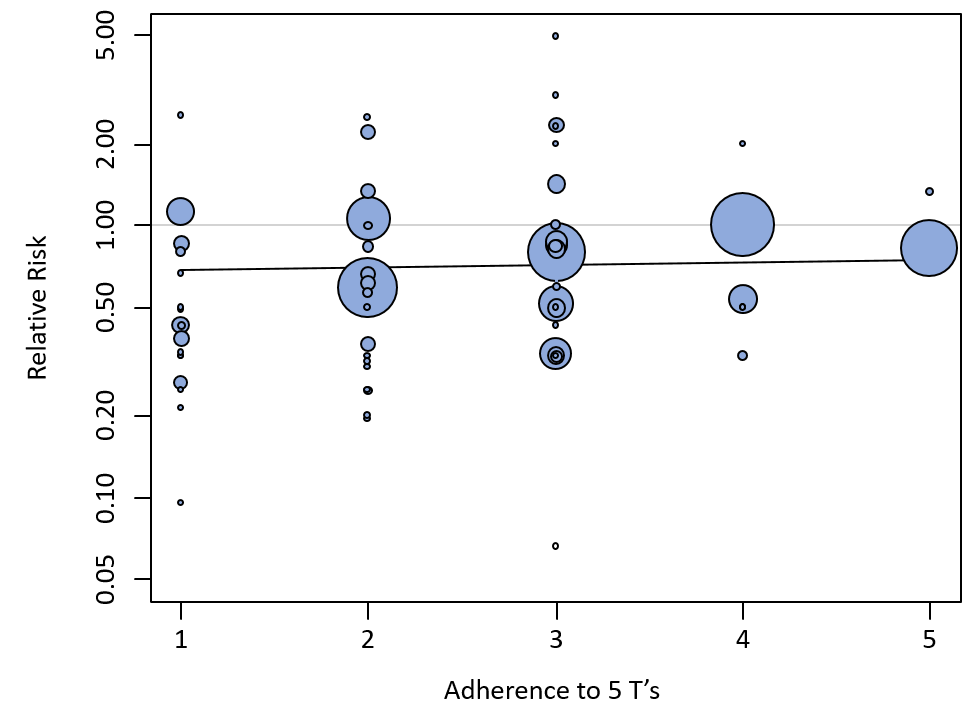


# **Appendix 8. Subgroup analysis for the type of surgery**

Relative risk (RR) of studies including cardiothoracic, gastrointestinal, or other surgeries. The figure shows the pooled RR. The squares and horizontal lines represent point estimates and corresponding 95% confidence intervals (CI) of the individual studies.


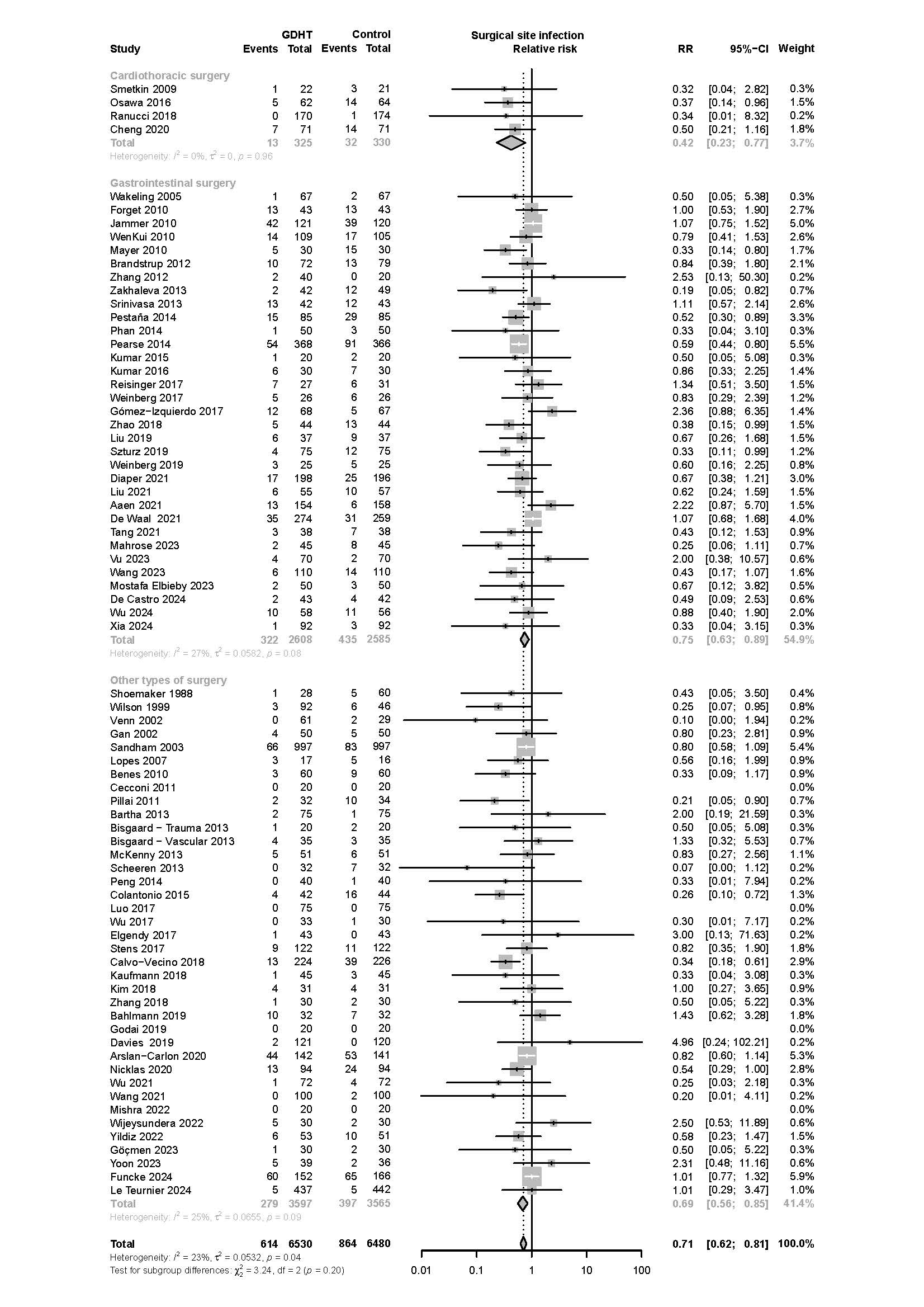


# **Appendix 9. Subgroup analysis for the level of wound contamination**

Relative risk (RR) of studies grouped for those including clean surgery, clean-contaminated and contaminated surgery, or mixed contaminated surgery. The figure shows the pooled RR. The squares and horizontal lines represent point estimates and corresponding 95% confidence intervals (CI) of the individual studies.

**
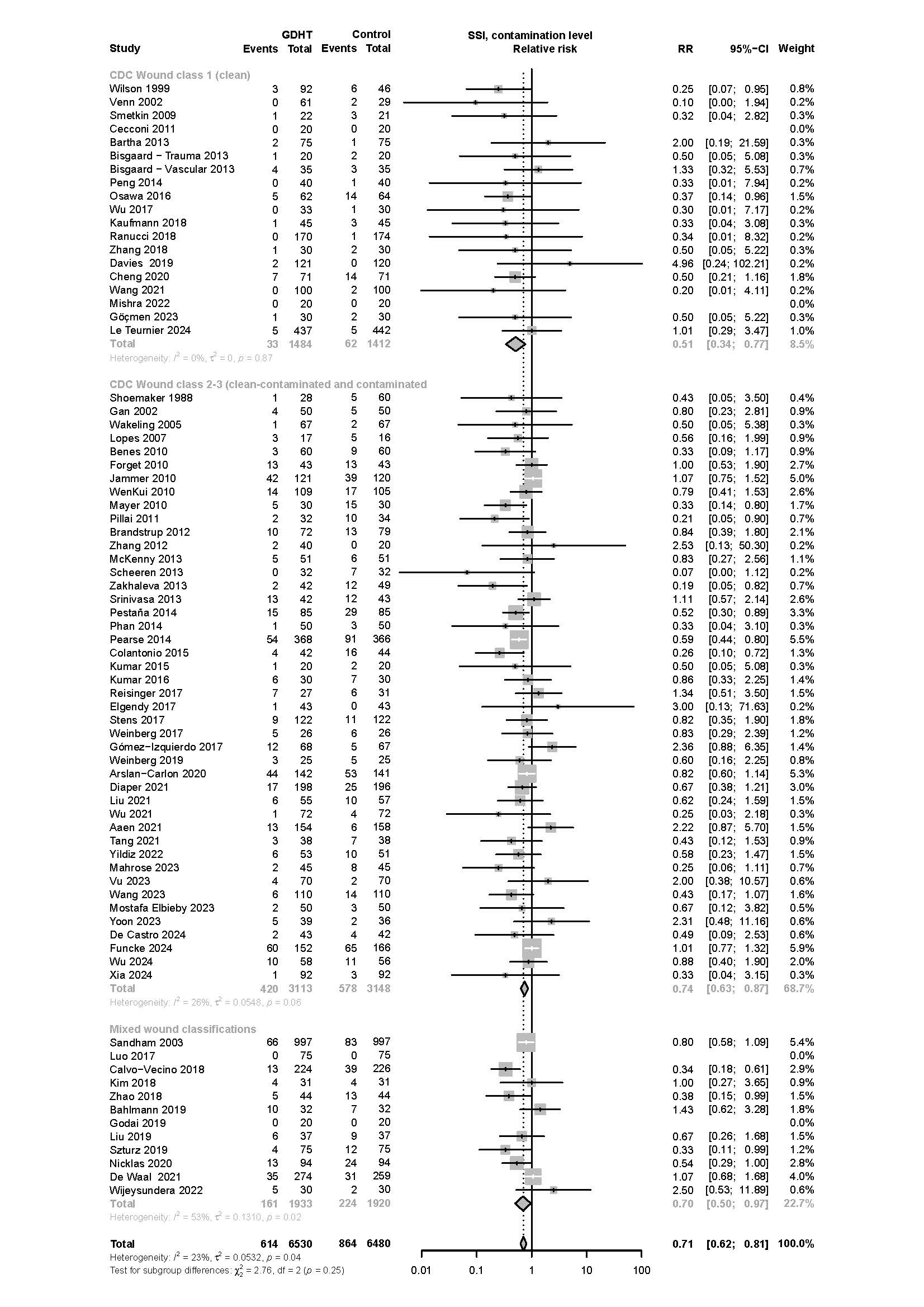
**

# **Appendix 10. Trial sequential analysis for the primary outcome surgical site infection**

**10A. Trial sequential analysis of all randomised controlled trials**

Trial sequential analysis was based on a relative risk reduction of 25%, surgical site infection risk in the control group of 13.3%, a type I error of 5% and a type II error of 20%. The red horizontal lines represents the Z-score thresholds of -1.96 and 1.96 for significance. The red vertical line represents diversity-adjusted required information size (DARIS) of 4706 patients. The cumulative Z-curve crosses the benefit boundary before the DARIS, favouring goal-directed haemodynamic therapy (GDHT).


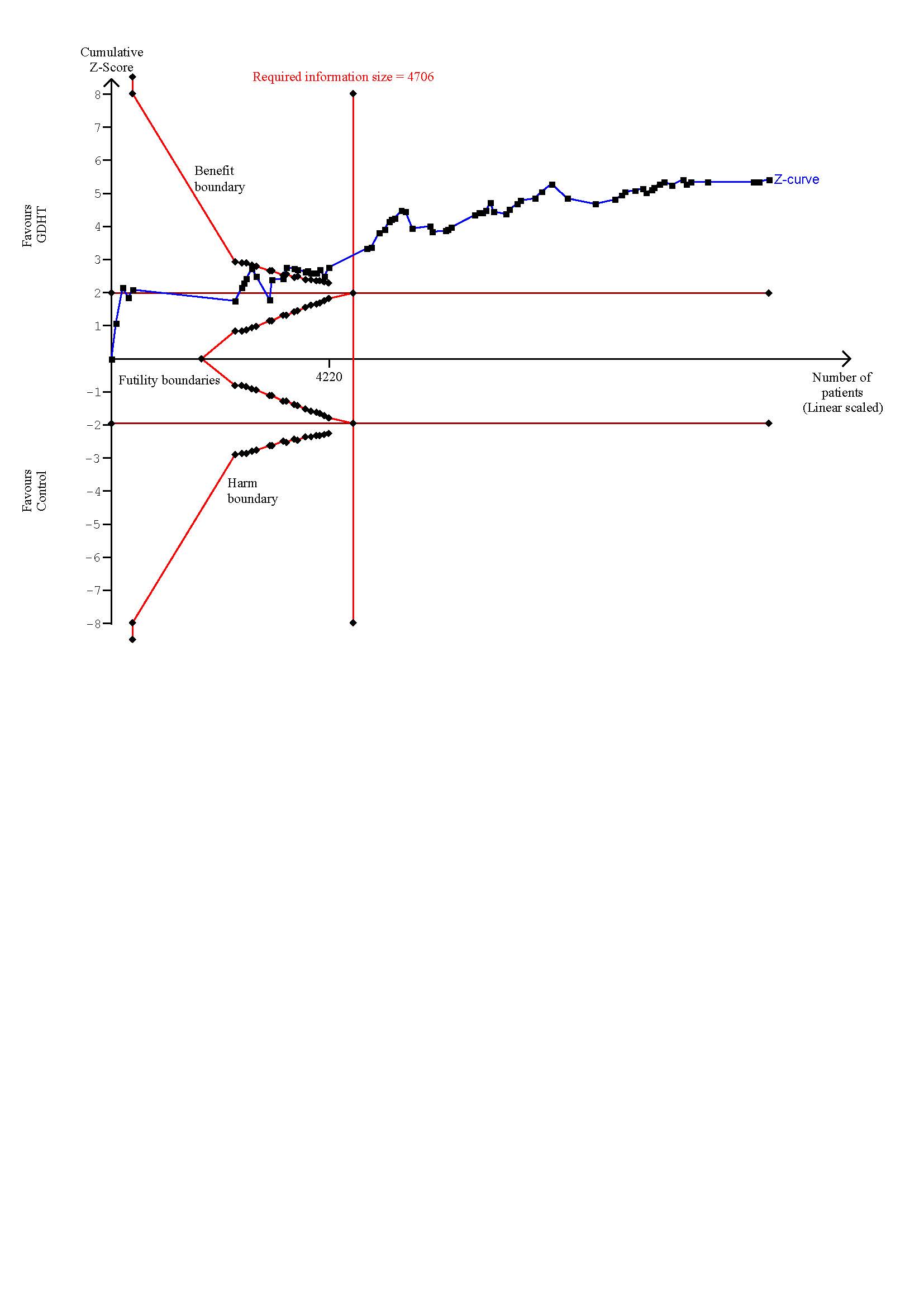


**10B. Trial sequential analysis of only randomised controlled trials studies with low risk of bias**

Trial sequential analysis was based on a relative risk reduction of 25%, surgical site infection risk in the control group of 14.1%, a type I error of 5% and a type II error of 20%. The red horizontal lines represents the Z-score thresholds of -1.96 and 1.96 for significance. The red vertical line represents diversity-adjusted required information size (DARIS) of 4644 patients. The cumulative Z-curve crosses the benefit boundary before the DARIS, favouring goal-directed haemodynamic therapy (GDHT).


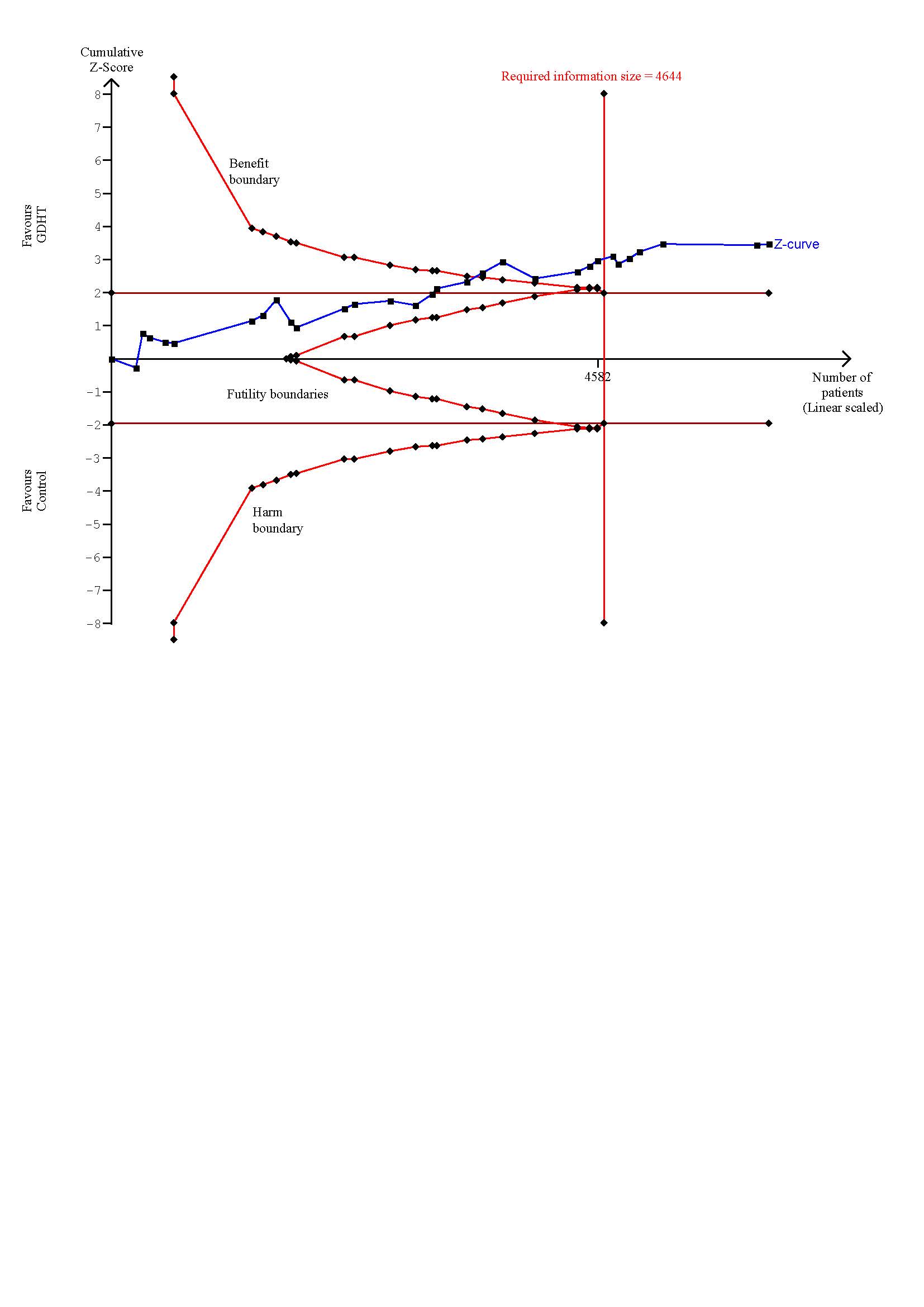


# **Appendix 11. Comparison-adjusted funnel plot**

The comparison-adjusted funnel plot shows the effect estimate of a study (relative risks) versus its precision (standard error) for surgical site infection (SSI). The funnel plot shows symmetry, indicating no differences between small and large studies regarding the effect of the treatment (small-study effect). Comparison-adjusted funnel plot asymmetry can be caused by publication bias. Since we find no asymmetry (no small-study effect), publication bias is less likely.


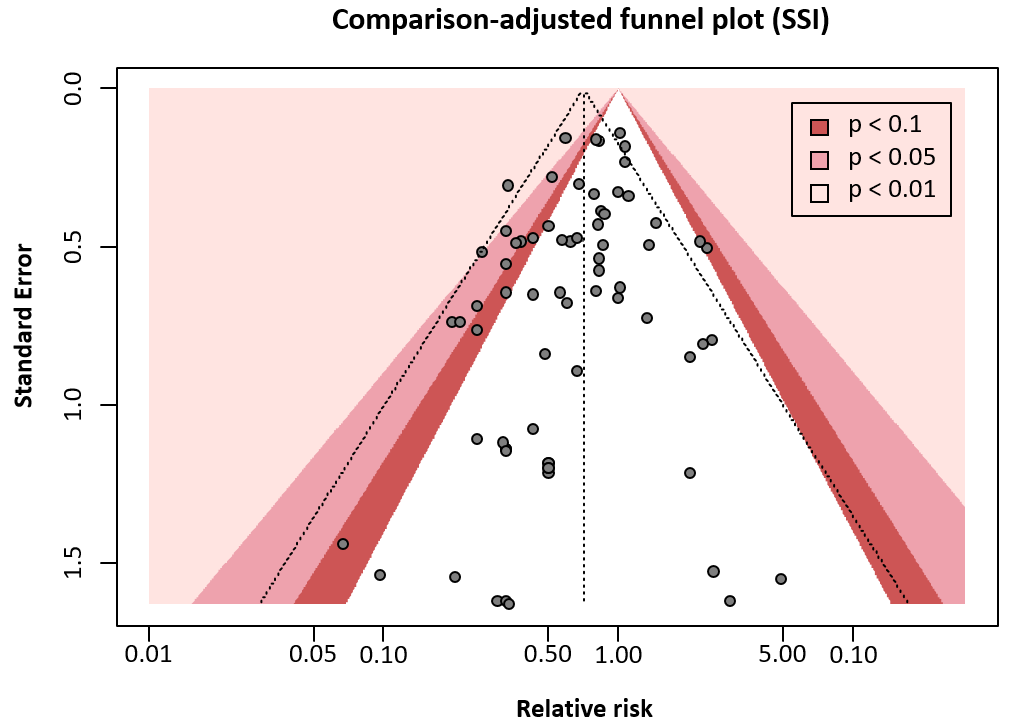

Supplement: Supplementary Figures and Tables [file mmc1.docx]
